# The GenTree Platform: growth traits and tree-level environmental data in 12 European forest tree species

**DOI:** 10.1093/gigascience/giab010

**Published:** 2021-03-18

**Authors:** Lars Opgenoorth, Benjamin Dauphin, Raquel Benavides, Katrin Heer, Paraskevi Alizoti, Elisabet Martínez-Sancho, Ricardo Alía, Olivier Ambrosio, Albet Audrey, Francisco Auñón, Camilla Avanzi, Evangelia Avramidou, Francesca Bagnoli, Evangelos Barbas, Cristina C Bastias, Catherine Bastien, Eduardo Ballesteros, Giorgia Beffa, Frédéric Bernier, Henri Bignalet, Guillaume Bodineau, Damien Bouic, Sabine Brodbeck, William Brunetto, Jurata Buchovska, Melanie Buy, Ana M Cabanillas-Saldaña, Bárbara Carvalho, Nicolas Cheval, José M Climent, Marianne Correard, Eva Cremer, Darius Danusevičius, Fernando Del Caño, Jean-Luc Denou, Nicolas di Gerardi, Bernard Dokhelar, Alexis Ducousso, Anne Eskild Nilsen, Anna-Maria Farsakoglou, Patrick Fonti, Ioannis Ganopoulos, José M García del Barrio, Olivier Gilg, Santiago C González-Martínez, René Graf, Alan Gray, Delphine Grivet, Felix Gugerli, Christoph Hartleitner, Enja Hollenbach, Agathe Hurel, Bernard Issehut, Florence Jean, Veronique Jorge, Arnaud Jouineau, Jan-Philipp Kappner, Katri Kärkkäinen, Robert Kesälahti, Florian Knutzen, Sonja T Kujala, Timo A Kumpula, Mariaceleste Labriola, Celine Lalanne, Johannes Lambertz, Martin Lascoux, Vincent Lejeune, Gregoire Le-Provost, Joseph Levillain, Mirko Liesebach, David López-Quiroga, Benjamin Meier, Ermioni Malliarou, Jérémy Marchon, Nicolas Mariotte, Antonio Mas, Silvia Matesanz, Helge Meischner, Célia Michotey, Pascal Milesi, Sandro Morganti, Daniel Nievergelt, Eduardo Notivol, Geir Ostreng, Birte Pakull, Annika Perry, Andrea Piotti, Christophe Plomion, Nicolas Poinot, Mehdi Pringarbe, Luc Puzos, Tanja Pyhäjärvi, Annie Raffin, José A Ramírez-Valiente, Christian Rellstab, Dourthe Remi, Sebastian Richter, Juan J Robledo-Arnuncio, Sergio San Segundo, Outi Savolainen, Silvio Schueler, Volker Schneck, Ivan Scotti, Vladimir Semerikov, Lenka Slámová, Jørn Henrik Sønstebø, Ilaria Spanu, Jean Thevenet, Mari Mette Tollefsrud, Norbert Turion, Giovanni Giuseppe Vendramin, Marc Villar, Georg von Arx, Johan Westin, Bruno Fady, Tor Myking, Fernando Valladares, Filippos A Aravanopoulos, Stephen Cavers

**Affiliations:** Philipps University Marburg, Faculty of Biology, Plant Ecology and Geobotany, Karl-von-Frisch Strasse 8, 35043, Marburg, Germany; Swiss Federal Research Institute WSL, Zürcherstrasse 111, 8903, Birmensdorf, Switzerland; Swiss Federal Research Institute WSL, Zürcherstrasse 111, 8903, Birmensdorf, Switzerland; LINCGlobal, Department of Biogeography and Global Change, Museo Nacional de Ciencias Naturales, CSIC, Serrano 115 dpdo, 28006, Madrid, Spain; Philipps University Marburg, Faculty of Biology, Plant Ecology and Geobotany, Karl-von-Frisch Strasse 8, 35043, Marburg, Germany; Aristotle University of Thessaloniki, School of Forestry and Natural Environment, Laboratory of Forest Genetics and Tree Improvement, 54124, Thessaloniki, Greece; Swiss Federal Research Institute WSL, Zürcherstrasse 111, 8903, Birmensdorf, Switzerland; Instituto Nacional de Investigación y Tecnología Agraria y Alimentaria - Centro de Investigación Forestal (INIA-CIFOR), Ctra. de la Coruña km 7.5, 28040, Madrid, Spain; Institut National de Recherche en Agriculture, Alimentation et Environment (INRAE), Domaine Saint Paul, Site Agroparc, 84914, Avignon, France; Institut National de Recherche en Agriculture, Alimentation et Environment (INRAE), UEFP, 33610, Cestas, France; Instituto Nacional de Investigación y Tecnología Agraria y Alimentaria - Centro de Investigación Forestal (INIA-CIFOR), Ctra. de la Coruña km 7.5, 28040, Madrid, Spain; Institute of Biosciences and BioResources, National Research Council (CNR), via Madonna del Piano 10, 50019, Sesto, Fiorentino, Italy; Aristotle University of Thessaloniki, School of Forestry and Natural Environment, Laboratory of Forest Genetics and Tree Improvement, 54124, Thessaloniki, Greece; Institute of Biosciences and BioResources, National Research Council (CNR), via Madonna del Piano 10, 50019, Sesto, Fiorentino, Italy; Aristotle University of Thessaloniki, School of Forestry and Natural Environment, Laboratory of Forest Genetics and Tree Improvement, 54124, Thessaloniki, Greece; Centre d'Ecologie Fonctionnelle et Evolutive (CEFE), CNRS, UMR 5175, 34090, Montpellier, France; Institut National de Recherche en Agriculture, Alimentation et Environment (INRAE), Dept ECOFA, 45075, Orléans, France; Instituto Nacional de Investigación y Tecnología Agraria y Alimentaria - Centro de Investigación Forestal (INIA-CIFOR), Ctra. de la Coruña km 7.5, 28040, Madrid, Spain; Swiss Federal Research Institute WSL, Zürcherstrasse 111, 8903, Birmensdorf, Switzerland; Institut National de Recherche en Agriculture, Alimentation et Environment (INRAE), UEFP, 33610, Cestas, France; Institut National de Recherche en Agriculture, Alimentation et Environment (INRAE), UEFP, 33610, Cestas, France; Institut National de Recherche en Agriculture, Alimentation et Environment (INRAE), GBFOR, 45075, Orléans, France; Institut National de Recherche en Agriculture, Alimentation et Environment (INRAE), UEFP, 33610, Cestas, France; Swiss Federal Research Institute WSL, Zürcherstrasse 111, 8903, Birmensdorf, Switzerland; Institut National de Recherche en Agriculture, Alimentation et Environment (INRAE), Domaine Saint Paul, Site Agroparc, 84914, Avignon, France; Vytautas Magnus University, Studentu Street 11, 53361, Akademija, Lithuania; Institut National de Recherche en Agriculture, Alimentation et Environment (INRAE), Domaine Saint Paul, Site Agroparc, 84914, Avignon, France; Departamento de Agricultura, Ganadería y Medio Ambiente, Gobierno de Aragón, P. Mª Agustín 36, 50071, Zaragoza, Spain; LINCGlobal, Department of Biogeography and Global Change, Museo Nacional de Ciencias Naturales, CSIC, Serrano 115 dpdo, 28006, Madrid, Spain; Institut National de Recherche en Agriculture, Alimentation et Environment (INRAE), UEFP, 33610, Cestas, France; Instituto Nacional de Investigación y Tecnología Agraria y Alimentaria - Centro de Investigación Forestal (INIA-CIFOR), Ctra. de la Coruña km 7.5, 28040, Madrid, Spain; Institut National de Recherche en Agriculture, Alimentation et Environment (INRAE), Domaine Saint Paul, Site Agroparc, 84914, Avignon, France; Bavarian Institute for Forest Genetics, Forstamtsplatz 1, 83317, Teisendorf, Germany; Vytautas Magnus University, Studentu Street 11, 53361, Akademija, Lithuania; Instituto Nacional de Investigación y Tecnología Agraria y Alimentaria - Centro de Investigación Forestal (INIA-CIFOR), Ctra. de la Coruña km 7.5, 28040, Madrid, Spain; Institut National de Recherche en Agriculture, Alimentation et Environment (INRAE), UEFP, 33610, Cestas, France; Swiss Federal Research Institute WSL, Zürcherstrasse 111, 8903, Birmensdorf, Switzerland; Institut National de Recherche en Agriculture, Alimentation et Environment (INRAE), UEFP, 33610, Cestas, France; INRAE, Univsité de Bordeaux, BIOGECO, 33770, Cestas, France; Division of Forestry and Forest Resources, Norwegian Institute of Bioeconomy Research (NIBIO), P.O. Box 115, 1431, Ås, Norway; Aristotle University of Thessaloniki, School of Forestry and Natural Environment, Laboratory of Forest Genetics and Tree Improvement, 54124, Thessaloniki, Greece; Swiss Federal Research Institute WSL, Zürcherstrasse 111, 8903, Birmensdorf, Switzerland; Institute of Plant Breeding and Genetic Resources, Hellenic Agricultural Organization DEMETER (ex NAGREF), 57001, Thermi, Greece; Instituto Nacional de Investigación y Tecnología Agraria y Alimentaria - Centro de Investigación Forestal (INIA-CIFOR), Ctra. de la Coruña km 7.5, 28040, Madrid, Spain; Institut National de Recherche en Agriculture, Alimentation et Environment (INRAE), Domaine Saint Paul, Site Agroparc, 84914, Avignon, France; Institut National de Recherche en Agriculture, Alimentation et Environment (INRAE), UEFP, 33610, Cestas, France; Swiss Federal Research Institute WSL, Zürcherstrasse 111, 8903, Birmensdorf, Switzerland; UK Centre for Ecology and Hydrology, Bush Estate Penicuik, EH26 0QB, Edinburgh, UK; Instituto Nacional de Investigación y Tecnología Agraria y Alimentaria - Centro de Investigación Forestal (INIA-CIFOR), Ctra. de la Coruña km 7.5, 28040, Madrid, Spain; Swiss Federal Research Institute WSL, Zürcherstrasse 111, 8903, Birmensdorf, Switzerland; LIECO GmbH & Co KG; Philipps University Marburg, Faculty of Biology, Plant Ecology and Geobotany, Karl-von-Frisch Strasse 8, 35043, Marburg, Germany; Institut National de Recherche en Agriculture, Alimentation et Environment (INRAE), UEFP, 33610, Cestas, France; Institut National de Recherche en Agriculture, Alimentation et Environment (INRAE), UEFP, 33610, Cestas, France; Institut National de Recherche en Agriculture, Alimentation et Environment (INRAE), Domaine Saint Paul, Site Agroparc, 84914, Avignon, France; Institut National de Recherche en Agriculture, Alimentation et Environment (INRAE), ONF, BIOFORA, 45075, Orléans, France; Institut National de Recherche en Agriculture, Alimentation et Environment (INRAE), Domaine Saint Paul, Site Agroparc, 84914, Avignon, France; Philipps University Marburg, Faculty of Biology, Plant Ecology and Geobotany, Karl-von-Frisch Strasse 8, 35043, Marburg, Germany; Natural Resources Institute Finland, Paavo Havaksentie 3, 90014, University of Oulu, Finland; University of Oulu, Pentti Kaiteran katu 1, 90014, University of Oulu, Finland; Bavarian Institute for Forest Genetics, Forstamtsplatz 1, 83317, Teisendorf, Germany; Natural Resources Institute Finland, Paavo Havaksentie 3, 90014, University of Oulu, Finland; University of Oulu, Pentti Kaiteran katu 1, 90014, University of Oulu, Finland; Institute of Biosciences and BioResources, National Research Council (CNR), via Madonna del Piano 10, 50019, Sesto, Fiorentino, Italy; INRAE, Univsité de Bordeaux, BIOGECO, 33770, Cestas, France; Philipps University Marburg, Faculty of Biology, Plant Ecology and Geobotany, Karl-von-Frisch Strasse 8, 35043, Marburg, Germany; Department of Ecology & Genetics, EBC, Uppsala University, Norbyvägen 18D, 75236, Uppsala, Sweden; Institut National de Recherche en Agriculture, Alimentation et Environment (INRAE), GBFOR, 45075, Orléans, France; INRAE, Univsité de Bordeaux, BIOGECO, 33770, Cestas, France; Université de Lorraine, AgroParisTech, INRAE, SILVA, 54000, Nancy, France; Thünen Institute of Forest Genetics, Sieker Landstr. 2, 22927, Grosshansdorf, Germany; LINCGlobal, Department of Biogeography and Global Change, Museo Nacional de Ciencias Naturales, CSIC, Serrano 115 dpdo, 28006, Madrid, Spain; Swiss Federal Research Institute WSL, Zürcherstrasse 111, 8903, Birmensdorf, Switzerland; Aristotle University of Thessaloniki, School of Forestry and Natural Environment, Laboratory of Forest Genetics and Tree Improvement, 54124, Thessaloniki, Greece; Swiss Federal Research Institute WSL, Zürcherstrasse 111, 8903, Birmensdorf, Switzerland; Institut National de Recherche en Agriculture, Alimentation et Environment (INRAE), Domaine Saint Paul, Site Agroparc, 84914, Avignon, France; LINCGlobal, Department of Biogeography and Global Change, Museo Nacional de Ciencias Naturales, CSIC, Serrano 115 dpdo, 28006, Madrid, Spain; Área de Biodiversidad y Conservación, Universidad Rey Juan Carlos, Calle Tulipán s/n, 28933, Móstoles, Spain; Philipps University Marburg, Faculty of Biology, Plant Ecology and Geobotany, Karl-von-Frisch Strasse 8, 35043, Marburg, Germany; Institut National de Recherche en Agriculture, Alimentation et Environment (INRAE), URGI, Versailles, France; Department of Ecology & Genetics, EBC, Science for Life Laboratory, Uppsala University, 75236, Uppsala, Sweden; Swiss Federal Research Institute WSL, Zürcherstrasse 111, 8903, Birmensdorf, Switzerland; Swiss Federal Research Institute WSL, Zürcherstrasse 111, 8903, Birmensdorf, Switzerland; Centro de Investigación y Tecnología Agroalimentaria de Aragón - Unidad de Recursos Forestales (CITA), Avda. Montañana 930, 50059, Zaragoza, Spain; Division of Forestry and Forest Resources, Norwegian Institute of Bioeconomy Research (NIBIO), P.O. Box 115, 1431, Ås, Norway; Thünen Institute of Forest Genetics, Sieker Landstr. 2, 22927, Grosshansdorf, Germany; UK Centre for Ecology and Hydrology, Bush Estate Penicuik, EH26 0QB, Edinburgh, UK; Institute of Biosciences and BioResources, National Research Council (CNR), via Madonna del Piano 10, 50019, Sesto, Fiorentino, Italy; INRAE, Univsité de Bordeaux, BIOGECO, 33770, Cestas, France; Institut National de Recherche en Agriculture, Alimentation et Environment (INRAE), UEFP, 33610, Cestas, France; Institut National de Recherche en Agriculture, Alimentation et Environment (INRAE), Domaine Saint Paul, Site Agroparc, 84914, Avignon, France; Institut National de Recherche en Agriculture, Alimentation et Environment (INRAE), UEFP, 33610, Cestas, France; University of Oulu, Pentti Kaiteran katu 1, 90014, University of Oulu, Finland; Institut National de Recherche en Agriculture, Alimentation et Environment (INRAE), UEFP, 33610, Cestas, France; Instituto Nacional de Investigación y Tecnología Agraria y Alimentaria - Centro de Investigación Forestal (INIA-CIFOR), Ctra. de la Coruña km 7.5, 28040, Madrid, Spain; Swiss Federal Research Institute WSL, Zürcherstrasse 111, 8903, Birmensdorf, Switzerland; Institut National de Recherche en Agriculture, Alimentation et Environment (INRAE), UEFP, 33610, Cestas, France; Philipps University Marburg, Faculty of Biology, Plant Ecology and Geobotany, Karl-von-Frisch Strasse 8, 35043, Marburg, Germany; Instituto Nacional de Investigación y Tecnología Agraria y Alimentaria - Centro de Investigación Forestal (INIA-CIFOR), Ctra. de la Coruña km 7.5, 28040, Madrid, Spain; Instituto Nacional de Investigación y Tecnología Agraria y Alimentaria - Centro de Investigación Forestal (INIA-CIFOR), Ctra. de la Coruña km 7.5, 28040, Madrid, Spain; University of Oulu, Pentti Kaiteran katu 1, 90014, University of Oulu, Finland; Austrian Research Centre for Forests (BFW), Seckendorff-Gudent-Weg 8, 1131, Wien, Austria; Thünen Institute of Forest Genetics, Eberswalder Chaussee 3a, 15377, Waldsieversdorf, Germany; Institut National de Recherche en Agriculture, Alimentation et Environment (INRAE), Domaine Saint Paul, Site Agroparc, 84914, Avignon, France; Institute of Plant and Animal Ecology, Ural branch of RAS, 8 Marta St. 202, 620144, Ekaterinburg, Russia; Swiss Federal Research Institute WSL, Zürcherstrasse 111, 8903, Birmensdorf, Switzerland; Division of Forestry and Forest Resources, Norwegian Institute of Bioeconomy Research (NIBIO), P.O. Box 115, 1431, Ås, Norway; Institute of Biosciences and BioResources, National Research Council (CNR), via Madonna del Piano 10, 50019, Sesto, Fiorentino, Italy; Institut National de Recherche en Agriculture, Alimentation et Environment (INRAE), Domaine Saint Paul, Site Agroparc, 84914, Avignon, France; Division of Forestry and Forest Resources, Norwegian Institute of Bioeconomy Research (NIBIO), P.O. Box 115, 1431, Ås, Norway; Institut National de Recherche en Agriculture, Alimentation et Environment (INRAE), Domaine Saint Paul, Site Agroparc, 84914, Avignon, France; Institute of Biosciences and BioResources, National Research Council (CNR), via Madonna del Piano 10, 50019, Sesto, Fiorentino, Italy; Institut National de Recherche en Agriculture, Alimentation et Environment (INRAE), ONF, BIOFORA, 45075, Orléans, France; Swiss Federal Research Institute WSL, Zürcherstrasse 111, 8903, Birmensdorf, Switzerland; Skogforsk, Tomterna 1, 91821, Sävar, Sweden; Institut National de Recherche en Agriculture, Alimentation et Environment (INRAE), Domaine Saint Paul, Site Agroparc, 84914, Avignon, France; Division of Forestry and Forest Resources, Norwegian Institute of Bioeconomy Research (NIBIO), P.O. Box 115, 1431, Ås, Norway; LINCGlobal, Department of Biogeography and Global Change, Museo Nacional de Ciencias Naturales, CSIC, Serrano 115 dpdo, 28006, Madrid, Spain; Aristotle University of Thessaloniki, School of Forestry and Natural Environment, Laboratory of Forest Genetics and Tree Improvement, 54124, Thessaloniki, Greece; UK Centre for Ecology and Hydrology, Bush Estate Penicuik, EH26 0QB, Edinburgh, UK

**Keywords:** regeneration, DBH, height, crown size, bark thickness, fruit number, stem straightness, branch angle, forking index, soil depth

## Abstract

**Background:**

Progress in the field of evolutionary forest ecology has been hampered by the huge challenge of phenotyping trees across their ranges in their natural environments, and the limitation in high-resolution environmental information.

**Findings:**

The GenTree Platform contains phenotypic and environmental data from 4,959 trees from 12 ecologically and economically important European forest tree species: *Abies alba* Mill. (silver fir), *Betula pendula* Roth. (silver birch), *Fagus sylvatica* L. (European beech), *Picea abies* (L.) H. Karst (Norway spruce), *Pinus cembra* L. (Swiss stone pine), *Pinus halepensis* Mill. (Aleppo pine), *Pinus nigra* Arnold (European black pine), *Pinus pinaster* Aiton (maritime pine), *Pinus sylvestris* L. (Scots pine), *Populus nigra* L. (European black poplar), *Taxus baccata* L. (English yew), and *Quercus petraea* (Matt.) Liebl. (sessile oak). Phenotypic (height, diameter at breast height, crown size, bark thickness, biomass, straightness, forking, branch angle, fructification), regeneration, environmental *in situ* measurements (soil depth, vegetation cover, competition indices), and environmental modeling data extracted by using bilinear interpolation accounting for surrounding conditions of each tree (precipitation, temperature, insolation, drought indices) were obtained from trees in 194 sites covering the species’ geographic ranges and reflecting local environmental gradients.

**Conclusion:**

The GenTree Platform is a new resource for investigating ecological and evolutionary processes in forest trees. The coherent phenotyping and environmental characterization across 12 species in their European ranges allow for a wide range of analyses from forest ecologists, conservationists, and macro-ecologists. Also, the data here presented can be linked to the GenTree Dendroecological collection, the GenTree Leaf Trait collection, and the GenTree Genomic collection presented elsewhere, which together build the largest evolutionary forest ecology data collection available.

## Context

The impacts of climate change and land use change on forests are already severe, as observed, e.g., following the extreme summer drought of 2018 that triggered a massive increase in mortality in Central European forests [1]. Furthermore, changes are expected to be acute in the future, altering distribution ranges and ecosystem functioning, as well as the interactions among species [2]. Forecasts indicate that near-surface temperature will shift poleward at mean rates of 80–430 m yr^−1^ for temperate forests during the 21st century [3]. This translates into northward shifts of trees’ bioclimatic envelopes of 300–800 km within 1 century [3]. More importantly, the frequency and intensity of drought events, heat waves, forest fires, and pest outbreaks [4] are expected to increase.

In the light of these changes, species and forest ecosystem resilience will depend on the extent and structure of phenotypic plasticity, genetic variation, and adaptive potential, as well as dispersal ability. From the results of extensive networks of field experiments (provenance trials), it has long been shown that tree species are locally adapted at multiple spatial scales. In Europe, where most tree populations have established following post-glacial recolonization, such patterns of local adaptation must have developed rapidly and despite long generation time and extensive gene flow [5], a process enabled by high levels of within-population plasticity, genetic and epigenetic variation, and large population sizes [6]. Recent work has shown that genetic variation for stress response may be strongly structured along environmental gradients, such as water availability [7], temperature [8], or photoperiod [9]. However, the spatial patterns of current adaptation in particular phenotypic traits are only partly informative regarding the potential for future adaptation under a changing climate. To advance our understanding of the adaptive potential of trees, it is crucial to evaluate multiple traits in parallel to be able to model their putative response to new environmental conditions.

Recently, substantial effort has been made to identify specific genes and gene combinations that have undergone selection, by associating mutations at candidate loci with phenotypes related to stress events [10,11] or with environmental variables [12]. This latter example by Yeaman and co-workers [12] is one of the first association studies in forest tree species on a large genomic scale and the first to investigate convergent local adaptation in distantly related tree species. However, progress in this field has been hampered by limited genomic resources, the lack of small-scale, individual tree-level environmental information [13], and the huge challenge of phenotyping trees in their natural environments [14,15].

The GenTree Platform aims to address these challenges by providing individual-level, high-resolution phenotypic and environmental data for a set of up to 20 sampling sites for each of 12 ecologically and economically important forest tree species across Europe. For a subset of 7 species (*B. pendula, F. sylvatica, P. abies, P. pinaster, P. sylvestris, P. nigra*, and *Q. petraea*), the sampling of sites was carried out in pairs, i.e., contained 2 stands that were close enough to be connected by gene flow but situated in contrasting environments.

The sampling design described here was used for collecting phenotypic traits and ecological data. Also, tree ring and wood density measurements for the same trees were assessed [16], and datasets on leaf traits, including specific leaf area and isotopic content [17], as well as high-density single-nucleotide polymorphism data for each tree, were established, that will be published in GeneBank. All data and metadata information are gathered in the GnpIS repository [33], which makes updates possible [18].

We investigated the extent to which other datasets comparable to the data presented here exist by screening our 12 species in the TRY Plant Trait Data Base, the International Tree-Ring Data Bank, and the Biomass And Allometry Database for woody plants (BAAD). While this is a systematic approach, it leaves out a large number of tree species and therefore we cannot claim to have a comprehensive overview of the existing data. However, all 3 databases are large collections that include at least some of the tree measurements that we present. Even though these are tremendous resources, the major difference is that owing to their nature as collecting points of numerous independent datasets, there is no coherent sampling scheme in these collections as such, meaning that the number of trees per site, the method of tree selection, measured phenotypes, and provided environmental information vary greatly and therefore do not allow for coherent comparative analyses such as those of the GenTree Platform. For example, BAAD reports diameter at breast height (DBH) data for only 4 of the species presented here, namely, *B. pendula* with 3 populations, *F. sylvatica* with 2 populations, *P. abies* with 4 populations, and *P. sylvestris* with 10 populations. In the larger TRY database, all of our species are represented, but the variability of sampling schemes is much more heterogeneous concerning traits, number of populations per species, and metadata. For example, DBH measurements are being reported 232 times from a total of 12 *B. pendula* populations. Of these, almost all of the 170 measurements are from 1 population while from many other populations only 1 or up to 5 measurements are reported. Also, the measurements stem from 5 different original studies and thus have very different levels of additional information. We conclude that the core value of our reported data lies in the coherent sampling design, as well as the large number of sampled populations and individuals per species.

## Methods

A machine readable summary of the GenTree data is provided in Table 1. All recorded parameters are listed in Table 2.

**Table 1:** Machine readable data summary

Measurements	Vegetation cover, rock cover, soil depth, competition index, regeneration, diameter at breast height, height, crown size, bark thickness, number of fruits, stem straightness, branch angle, forking index
Technology types	Bark gauges, calculations, caliper, clinometer, GPS device, increment corer, laser distance measurement, telescopic measuring pole
Factor Types	Tree species
Sample characteristic organism	*Abies alba, Betula pendula, Fagus sylvatica, Picea abies, Pinus cembra, Pinus halepensis, Pinus nigra, Pinus pinaster, Pinus sylvestris, Populus nigra, Taxus baccata, Quercus petraea*
Sample characteristic location	Europe

**Table 2:** Variables names, explanations, and specifications measured for all 4,959 trees and all 194 GenTree sites

Variable name	Variable explanation	Specification
GenTree Platform metadata		
m01.spec	Species abbreviations	*Abies Alba* (AA), *Betula pendula* (BP), *Fagus sylvatica* (FS), *Picea abies* (PA), *Pinus cembra* (PC), *Pinus halepensis* (PH), *Pinus nigra* (PN), *Populus nigra* (PO), *Pinus pinaster* (PP), *Pinus sylvestris* (PS), *Quercus petraea* (QP), *Taxus baccata* (TB)
m02.country	Country abbreviations	Isocode 6133–2; Austria (AT), Switzerland (CH), Germany (DE), Spain (ES), Finland (FI), France (FR), Great Britain (GB), Greece (GR), Italy (IT), Lithuania (LT), Norway (NO), Sweden (SE)
m03.site.num	Site numbers	Running numbers of sites per species 01–24
m04.site.id	Complete site-ID per species	Merger of m01–m03
m05.tree.num	Tree numbers	Running numbers within sites 01–25
m06.tree.id	Complete tree ID	Merger of m01–m03, m05
m07.trial.name	Site name	
m08.lat	Latitude	Decimal degrees, WGS84
m09.lon	Longitude	Decimal degrees, WGS84
GenTree Platform phenotypes		
p01.height	Height	Tree height, m
p02.dbh	DBH	Diameter at breast height, cm
p03.bark	Bark thickness mean	Mean value of bark thickness, cm
p04.trunk	Trunk straightness/flexuosity	5: Absolutely straight; 4: fairly straight (in 1 direction slightly crooked); 3: slight to moderate bend in different directions; 2: moderate or strong bends; 1: no straight stem
p05.branch	Branch angle	1: <23° (steep); 2: 23–45°; 3: 45–67°; 4: 67–90° (plain); 5: >90°
p06.fork	Forking index	1: Fork at the lower third of tree height; 2: fork at middle third; 3: fork at upper third; 4: no fork—multiplied by 10 and then divided by the number of stems
p07.canopy.1	Canopy projection REP 1	Crown diameter projection, m
p08.canopy.2	Canopy projection REP 2	Crown diameter projection, m
p09.crown.ellipse	Crown ellipse	Area of an ellipse (d_i_/2)*(d_j_/2)*π, m^2^
p10.crown.round	Crown size	As some only have 1 diameter, round areas with the mean diameter [(d_i_+d_j_)/2]^2^*π, m^2^
p11.regeneration	Natural regeneration	1: Absent; 2: scattered; 3: groups; 4: abundant
p12.fruit.mean	Fruit/cone number	Number of fruits
p13.basal.area		
GenTree Platform *in situ* environmental measurements		
e01.plant.cover	Total plant cover	1: None; 2: little (5–20%); 3: low (20–40%); 4: medium (40–60%); 5: high (60–80%); 6: very high (80–95%); 7: full cover (>95%)
e02.comp.index.a	Competition index A	CI assessed following Canham et al. [23], and multi-stems as the sum
e03.comp.index.b	Competition index B	CI assessed following Canham et al. [23], and multi-stems assessing the sum of basal areas and then the DBH
e04.comp.index.c	Competition index C	CI assessed following Lorimer [24], and multi-stems as the sum
e05.comp.index.d	Competition index D	CI assessed following Lorimer [24], multi-stems assessing the sum of basal areas and then the DBH
e06.status		Dominant, co-dominant
e07.elevation	Elevation of the tree	Meters above sea level
e08.slope	Slope at the tree level	Slope in degrees
e09.aspect	Aspect at the tree level	0–360°
e10.soil.depth	Mean soil depth	Mean of 3 measures (measurement to a maximum depth of 60 cm)
e11.stone.content	Mean stone content	Mean of 3 measures: 1: none; 2: little (5–20%); 3: low (20–40%); 4: medium (40–60%); 5: high (60–80%); 6: very high (80–95%); 7: full cover (>95%)
e12.rock.cover	Total rock cover	1: None; 2: little (5–20%); 3: low (20–40%); 4: medium (40–60%); 5: high (60–80%); 6: very high (80–95%); 7: full cover (>95%)

### Sampling strategy

To optimize the sampling design for genome scans and association studies, we followed the recent theoretical work by Lotterhos and Whitlock [19,20], which indicates that a paired sampling design has more power to detect the genomic signatures of local adaptation. Using this framework, populations from across the natural range of a species are sampled in pairs, with the 2 sites in each pair situated geographically close enough to be genetically similar at neutral genes owing to a common evolutionary history and ongoing gene flow, but in distinct selective niches such that the local fitness optimum differs between the 2 sites. This sampling confers more power to detect evidence of selection in the genome through either association with environmental or phenotypic variables or the detection of outliers (e.g., for genetic differentiation, *F*_ST_) [19, 20]. Trees are very amenable to a pairwise approach because they are known to be locally adapted, often at fine spatial scales [21,22] and irrespective of gene flow distances [6]. This strategy was followed for the aforementioned subset of 7 species for which genomic resources were available (i.e., full or draft genome).

**Figure 1:**
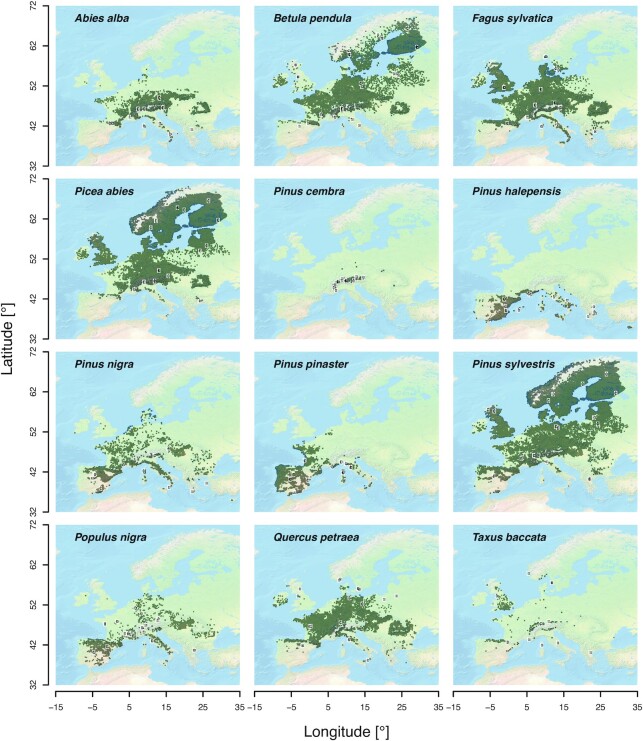
Sampling sites (black dots) and distributions of the 12 selected tree species (dark green shading) for *in situ* phenotype measurements. Distribution maps are based on a comprehensive high-resolution tree occurrence dataset from the European Union [30].

**Figure 2:**
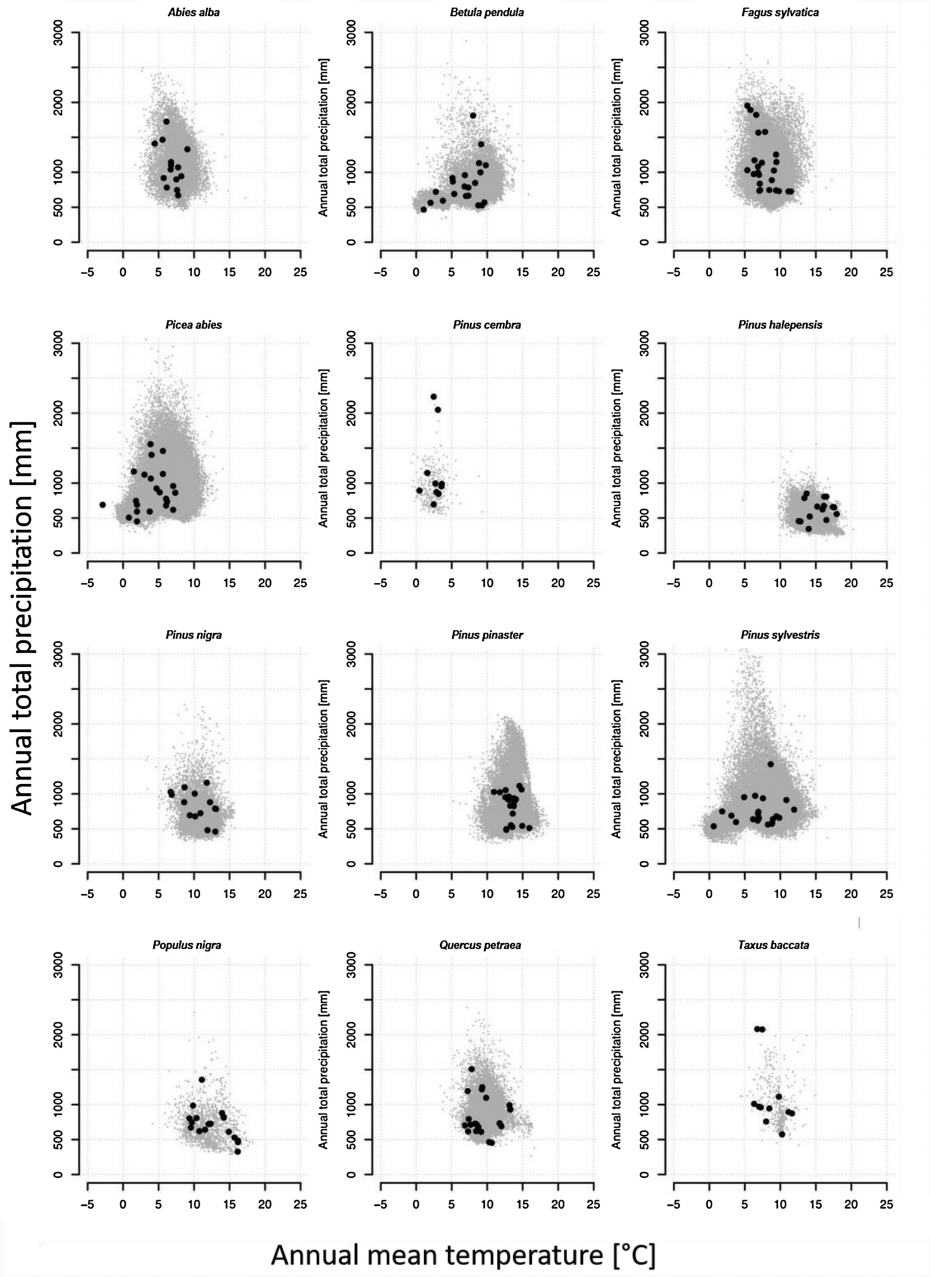
Climate-space diagrams for the 12 selected European tree species with annual mean temperature on the x-axis and annual total precipitation on the y-axis. Grey points represent species occurrences based on a comprehensive high-resolution tree occurrence dataset for Europe [30] and black dots indicate the GenTree sites.

Such local niche contrasts are neither easy to identify nor readily available when environments are homogenous. Therefore, a second principle of the sampling design was to cover a large part of each species’ natural geographic range (Fig. 1) and environmental space (Fig. 2) to capture selective niche variation. Finally, sites with a history of intensive management or any other intense and obvious anthropogenic or natural disturbances were avoided. This strategy was followed for all 12 species.

### Selection of trees on sites

A minimum of 25 trees was sampled per site to capture the natural phenotypic and genetic variability. Trees had to be mature but not senescent, dominant or codominant, and had to show no signs of significant damage due to pests and diseases or generally low vigor. Sampled trees were ≥30 m apart and, where possible, were chosen along several parallel linear transects across each site, typically resulting in 2–4 transects per sampling site to keep the overall sampling area <3 ha.

### Site and tree metadata

Sites were labeled by a 2-letter country code (ISO 3166–1 alpha-2) followed by a 2-letter species code and a 2-digit site number (Table 2). Individual tree labels added another 2-digit tree number. Every tree was permanently labeled so that future studies can resample subsets or the entire GenTree collection to gain time-series data of individual traits or to add new phenotypes to the analyses. Be aware that permission of the respective landowners must be obtained before sampling. Handheld GPS devices were used to record the position of each tree. The precision of GPS measurements in forests is notoriously challenging: regular commercial devices achieve an accuracy of ∼8–15 m with good satellite coverage. Given that trees were selected with a minimum distance of 30 m this accuracy was sufficient for the correct positioning of trees relative to each other. An overall population position was defined by taking the mean value across all the individual tree measurements. Coordinates were in decimal degrees with 4 decimal units to reflect the general measurement accuracy (∼11.1 m) and were stored in the WGS 84 reference system. GPS devices were also used to record the tree's elevation, either directly or through *post hoc* positioning in digital elevation models. The local aspect at the site of the tree was measured by a compass in 5° steps in the direction of the steepest slope.

The metadata for each site consists of an ID code (see above), sampling date, location (GPS coordinates, see above), and elevation in meters above sea level. Each stand was also characterized as being monospecific or mixed (in the latter case the most common co-occurring species was noted), stand structure was noted as single or multiple layered, and the age distribution as even or uneven (categorical variables).

### Competition index at tree level

Competition indices (CIs) were calculated following Canham et al. [23] and Lorimer [24]. Specifically, the first index following Lorimer [24] was calculated as $\,\,\mathrm{NCI}\,\, = \mathop \sum \nolimits_{i = 1}^5 \left(\mathrm{DB}{\mathrm{H}_j}/\mathrm{DB}{\mathrm{H}_i}\right)/\mathrm{dis}{\mathrm{t}_i}$ that follows the same notation as above, and where DBH is the DBH of the subject trees *j* and *i*.

Second, the distance-dependent competition index (NCI) following Canham et al. [23] was computed as $\mathrm{NCI} = \mathop \sum \nolimits_{i = 1}^5 ({\mathrm{DB}{\mathrm{H}_i}}/{\mathrm{dis}{\mathrm{t}_i}})$, where DBH*_i_* is the DBH of competitor tree *i* and dist*_i_* is the distance between the subject tree and competitor tree *i*. This index assumes that the net effects of neighboring trees vary as a direct function of the size of the neighbors and as an inverse function of the distance. For this purpose, the distance to the 5 nearest neighbors of each target tree was measured and their respective DBH was measured.

Moreover, it was noted whether competitor trees were conspecific to the target tree or not. Each multi-stemmed tree was considered as a single competitor where each stem of >15 cm DBH was measured and added to the sum of means.

### Environmental characteristics within subplots around each tree

Surrounding each target tree, slope, vegetation cover (without tree cover), and stone content were assessed in a 10 m × 10 m plot. The slope was assessed using a clinometer. Vegetation and rock cover were estimated in the classes <5%,  5–20%, 20–40%, 40–60%, 60–80%, 80–95%. Soil depth was estimated at 3 random points in the quadrat to a maximum of 60 cm with a pike and was averaged across these 3 values.

### Regeneration

In the same 10 m × 10 m plots, natural regeneration of the target species was assessed according to the following 4 classes: absent (no recruit visible), scattered (few/scattered individuals), grouped (presence of scattered groups within the plot), and abundant (regularly spread all over the plot) and is indicated in the database with values from 1 to 4. As this method cannot resolve maternity, the results indicate realized fecundity at the stand level.

### Growth traits

#### DBH

DBH in centimeters was measured at a stem height of 1.3 m either by using a caliper to measure 2 perpendicular diameters and subsequently taking the average of these 2 measurements or by measuring the circumference of the tree using a tape and computing the diameter from that value. Each measurement was performed to the nearest 0.1 cm. If a tree had >1 trunk, all of them were measured and the average was recorded.

#### Height

Height from the ground to the top of the crown in meters was measured using a hypsometer (Nikon forestry Pro Laser, Tokyo, Japan), a laser vertex (Haglof Vertex III, Langsele, Sweden), or a Laser Range Meter (Bosch GLM 50 C, Leinfelden-Echterdingen, Germany). For short trees, a telescopic measuring pole was used. Height was noted to the nearest 0.1 m. To forego errors introduced by measuring height on sloping ground, height measurements on slopes were conducted from the same elevation as the tree's base by approaching the tree sideways. Where this was not possible, a slope correction factor was used.

#### Crown size

The crown size in square meters was measured as the circular and ellipsoid plane area of the crown. For this, we measured 2 perpendicular crown diameters (canopy 1 and 2) with a measuring tape, with the first measurement being made along the longest axis of the crown, from 1 edge to the other, and by visually projecting the crown margin onto the ground to the nearest decimeter. For the ellipse area, we calculated $({d_i}/2)*({d_j}/2)*\pi $ and for the circular area (${d_i}$+${d_j}$/2)²*$\pi $.

#### Bark thickness

For measuring bark thickness in millimeters, we used bark gauges (Haglof Barktax, Langsele, Sweden) or a tape after extracting the bark with a small caliper (if bark could be detached without tree damage) or increment borers (Haglof increment borer, Langsele, Sweden) in case of strong and thick bark. Five measurements were taken for each tree at breast height and the average was calculated. For tree species with a clear dichotomy of bark thickness (e.g., old *P. nigra, T. baccata*), we included ≥2 measurements from the thinner and thicker bark areas each.

#### Number of fruits

In conifers, cones were counted by providing the average of 3 rounds of counting, made by an observer on the ground using binoculars. Only mature (brown) and closed cones were counted, i.e., those containing seeds, and not immature (green) or open cones, whose seeds had already been dispersed (open cones often stay on the branch for several years after seeds are dispersed). In broadleaves, the number of fruits was counted for 30 seconds, repeating the procedure 3 times to then average the 3 counts.

In the case of species with very small fruits that are hard to see individually and in locations with a very limited view of the canopy, each tree was assigned to 1 of 5 categories, namely, 0 (no fruits), 1 (a few fruits in a small section of the crown), 2 (a few fruits in ≥2 sections of the crown), 3 (a lot of fruits in a small section of the crown), and 4 (a lot of fruits in ≥2 sections of the crown).

#### Straightness

Straightness of the stem was classified according to 5 levels: (1) No straight stem, (2) moderate or strong bends, (3) slight to moderate bend in different directions, (4) fairly straight (in 1 direction slightly crooked), (5) absolutely straight. This was performed on the lower 15 m of the tree beginning from the ground with the crown not taken into account. In the case of forked stems, only the trunk below the deepest forking point was evaluated.

#### Branch angle

Branch angle was classified at 2 successive whorls according to a 5-scale scheme in conifers with (1) <23°,  (2) 23–45°, (3) 45–67°, (4) 67–90°, (5) >90°, and a 4-scale scheme in broadleaves omitting the >90° class. In black poplar, only the top 2 m of the crown were considered.

#### Forking index

The branching of a tree in 2 (fork) or more (ramiform) equally thick and long stems was assessed with a forking index. The index took into account 2 parameters. First a score for the relative position of the fork: (4) no forking, (3) forking in the upper third of the tree, (2) forking in the middle third of the tree, (1) forking in the lower third of the tree; and second the number of axes (stems). The score of the relative position was then multiplied by 10 and divided by the number of axes.

### Modeled environmental data extracted for GenTree sites

Topography, soil, and climate data were compiled to characterize environmental conditions in each GenTree sampling site as follows.

### Topography

We used the European digital elevation model to describe topographic conditions at 25 m spatial resolution with a vertical accuracy of approximately ±7 m (EU-DEM v. 1.1 from the Copernicus program [34]). We derived 14 variables (Table 3) based on biological hypotheses and their informative power at the local scale [25]. We calculated morphometric, hydrologic, and radiation grids for each GenTree site and visually inspected data integrity using SAGA 6.2 [26] (details in Table 3).

**Table 3:** Environmental variable names, explanations, and specifications modeled for all 4,959 trees and 194 GenTree sites

Variable		Specification	
Name	Explanation	Unit	Resolution (m)
GenTree Platform modeled environmental parameters			
Sample	Sample identification	None	None
Country	Country code	None	None
countryspecies	Country and species code	None	None
Species	Species code	None	None
Population	Population identification	None	None
latwgs84	Latitude in WGS84	Degree	25
lonwgs84	Longitude in WGS84	Degree	25
latetrs89	Latitude in ETRS89	Degree	25
lonetrs89	Longitude in ETRS89	Degree	25
t01alt	Altitude	m	25
t02slp	Slope	Degree	25
t03asp	Eastness	Degree	25
t04vcu	Profile curvature	Degree/m	25
t05hcu	Horizontal curvature	Degree/m	25
t06ddg	Downslope distance gradient	Degree	25
t07mpi	Morphometric protection index	None	25
t08tpi	Topographic position index	None	25
t09vrm	Vector ruggedness measure	None	25
t10twi	Topographic wetness index	None	25
t11svf	Sky-view factor	None	25
t12sdir	Potential direct solar radiation	kJ m^−2^	25
t13sdif	Potential diffuse solar radiation	kJ m^−2^	25
t14stot	Potential total solar radiation	kJ m^−2^	25
awc15	Available water capacity (0–30 cm)	%	250
awc140	Available water capacity (60–200 cm)	%	250
bio01	Yearly mean temperature	°C/10	1,000
bio02	Mean diurnal range	°C/10	1,000
bio03	Isothermality	°C/10	1,000
bio04	Temperature seasonality	°C/10	1,000
bio05	Max temperature of warmest month	°C/10	1,000
bio06	Min temperature of coldest month	°C/10	1,000
bio07	Temperature annual range	°C/10	1,000
bio08	Mean temperature of wettest quarter	°C/10	1,000
bio09	Mean temperature of driest quarter	°C/10	1,000
bio10	Mean temperature of warmest quarter	°C/10	1,000
bio11	Mean temperature of coldest quarter	°C/10	1,000
bio12	Yearly precipitation sum	kg m^−2^	1,000
bio13	Precipitation of wettest month	kg m^−2^	1,000
bio14	Precipitation of driest month	kg m^−2^	1,000
bio15	Precipitation seasonality	kg m^−2^	1,000
bio16	Precipitation of wettest quarter	kg m^−2^	1,000
bio17	Precipitation of driest quarter	kg m^−2^	1,000
bio18	Precipitation of warmest quarter	kg m^−2^	1,000
bio19	Precipitation of coldest quarter	kg m^−2^	1,000
Gdd	Growing degree days	°C	1,000
Gsp	Accumulated precipitation	kg m^−2^	1,000
Shc	Hydrothermic coefficient	(kg m^−2^/10)/°C	1,000
rh410	Relative humidity	%	1,000
Fcf	Frost change frequency	Number of events	1,000
Nfd	Number of frost days	Number of days	1,000
prec01	Precipitation sum in January	kg m^−2^	1,000
prec02	Precipitation sum in February	kg m^−2^	1,000
prec03	Precipitation sum in March	kg m^−2^	1,000
prec04	Precipitation sum in April	kg m^−2^	1,000
prec05	Precipitation sum in May	kg m^−2^	1,000
prec06	Precipitation sum in June	kg m^−2^	1,000
prec07	Precipitation sum in July	kg m^−2^	1,000
prec08	Precipitation sum in August	kg m^−2^	1,000
prec09	Precipitation sum in September	kg m^−2^	1,000
prec10	Precipitation sum in October	kg m^−2^	1,000
prec11	Precipitation sum in November	kg m^−2^	1,000
prec12	Precipitation sum in December	kg m^−2^	1,000
tmean01	Mean temperature in January	°C/10	1,000
tmean02	Mean temperature in February	°C/10	1,000
tmean03	Mean temperature in March	°C/10	1,000
tmean04	Mean temperature in April	°C/10	1,000
tmean05	Mean temperature in May	°C/10	1,000
tmean06	Mean temperature in June	°C/10	1,000
tmean07	Mean temperature in July	°C/10	1,000
tmean08	Mean temperature in August	°C/10	1,000
tmean09	Mean temperature in September	°C/10	1,000
tmean10	Mean temperature in October	°C/10	1,000
tmean11	Mean temperature in November	°C/10	1,000
tmean12	Mean temperature in December	°C/10	1,000
tmin01	Minimum temperature in January	°C/10	1,000
tmin02	Minimum temperature in February	°C/10	1,000
tmin03	Minimum temperature in March	°C/10	1,000
tmin04	Minimum temperature in April	°C/10	1,000
tmin05	Minimum temperature in May	°C/10	1,000
tmin06	Minimum temperature in June	°C/10	1,000
tmin07	Minimum temperature in July	°C/10	1,000
tmin08	Minimum temperature in August	°C/10	1,000
tmin09	Minimum temperature in September	°C/10	1,000
tmin10	Minimum temperature in October	°C/10	1,000
tmin11	Minimum temperature in November	°C/10	1,000
tmin12	Minimum temperature in December	°C/10	1,000
tmax01	Maximum temperature in January	°C/10	1,000
tmax02	Maximum temperature in February	°C/10	1,000
tmax03	Maximum temperature in March	°C/10	1,000
tmax04	Maximum temperature in April	°C/10	1,000
tmax05	Maximum temperature in May	°C/10	1,000
tmax06	Maximum temperature in June	°C/10	1,000
tmax07	Maximum temperature in July	°C/10	1,000
tmax08	Maximum temperature in August	°C/10	1,000
tmax09	Maximum temperature in September	°C/10	1,000
tmax10	Maximum temperature in October	°C/10	1,000
tmax11	Maximum temperature in November	°C/10	1,000
tmax12	Maximum temperature in December	°C/10	1,000

### Soil

We collected available data on water capacity at 7 soil depths using SoilGrids250m [27]. We estimated Pearson correlation coefficients, *r*, between soil layers and then averaged the 4 first superficial (0, 5, 15, and 30 cm) and the 3 deeper (60, 100, and 200 cm) layers that were highly correlated, respectively.

### Climate

We extracted climate data with a high spatial resolution (30 arcsec) using CHELSA v. 1.2 [28]. CHELSA is based on a quasi-mechanistic statistical downscaling global reanalysis and global circulation model that, in particular, reliably interpolates the amount of precipitation using an orographic rainfall and wind effect. The dataset consisted of 48 climatic, 19 bioclimatic, 4 drought- and 2 frost-related variables for the reference period 1979–2013 (Table 3  [35]). We extracted all modeled environmental values for each individually geo-referenced tree using the “extract” function of the R package raster [29]. The surrounding conditions (i.e., adjacent pixels) of each tree were incorporated by the bilinear interpolation method when extracting the data.

The local environmental contrasts varied among species and population pairs, most of which exhibited variability concerning elevation, temperature, precipitation, and water availability. Other local contrasts were based on radiation, soil water capacity, and topographic wetness index (among others). One special case is *P. nigra*, a heliophilous pioneer species found naturally in riverine areas. Given this specific habitat, local contrasts were largely bound to the distance of the individual trees from the riverbed and thus, e.g., to groundwater access or exposure to variation in the intensity and frequency of floods.

### Data validation and quality control

**Figure 3:**
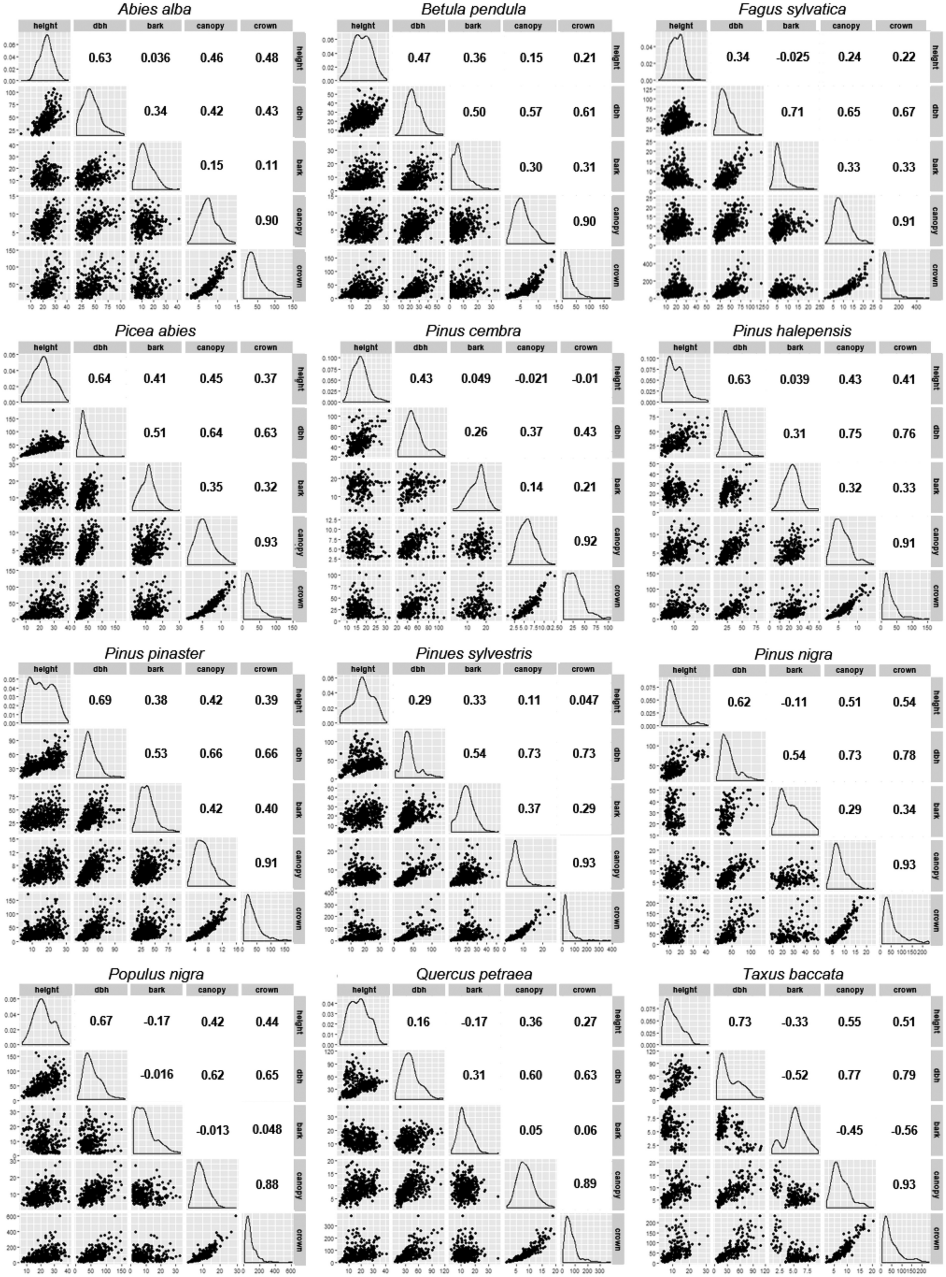
Scatterplots, distributions, and Pearson correlation coefficients, *r*, of GenTree phenotype measurements in the 12 selected European tree species.

The database has been checked for consistency at different stages by various researchers between 2018 and 2020. Raw data were submitted by all partners to the GnpIS multispecies integrative information system [36] using preformatted Microsoft Excel templates. Data files were harmonized, merged, and subsequently verified following several steps:

1. Missing data and dubious entries were checked manually by examining the original data files obtained from the partners and by cross-checking cases with field books.
2. Descriptive statistics were calculated and plotted for all variables including minima, maxima, means, and variances. Outliers were checked against original data records and corrected when necessary.
3. Covariables were plotted determining whether relationships were reasonable and following the most complete set of similar relationships (Fig. 3).

### Data Records

The data presented are structured in 4 independent csv files (GenTree_modelled_environmental_data.csv, GenTree_modelled_environmental_data_metadata.csv, GenTree_phenotypes_and_insitu_environmental_data.csv, and GenTree_phenotypes_and_insitu_environmental_data_metadata.csv) that can be merged using the site identifier (m04.site.id) or tree identifier (m06.tree.id). The same codes can be used to merge additional data, namely, from the GenTree Dendroecological Collection [16], the GenTree Leaf Trait Collection [17], and the GenTree Genomic Collection (T. Pyhäjärvi personal communication). The first file contains the modeled environmental data, the second its metadata, the third the individual phenotypic traits and the *in situ*environmental data, and the fourth the metadata of the latter.

## Data Availability

The data underlying this article are available in the GigaDB repository [31] under a CC0 license. Excel versions of the data are available from Figshare [32]. All the data are indexed in Table 3.

## Abbreviations

BAAD: Biomass And Allometry Database; CHELSA: Climatologies at High Resolution for the Earth’s Land Surface Areas; CI: competition index; DBH: diameter at breast height; GPS: Global Positioning System; ISO: International Organization for Standardization.

## Competing Interests

The authors declare that they have no competing interests.

## Funding

This publication is part of the GenTree project, which was funded by the European Union's Horizon 2020 research and innovation program under grant agreement No. 676876 (GenTree). This work was also supported by the Swiss Secretariat for Education, Research and Innovation (SERI) under contract No. 6.0032.

## Authors' Contributions

L.O., R.B., K.H., B.F., T.M., F.V., F.A.A., and S.C. coordinated sampling design. All authors contributed to the sampling design. L.O., R.B., K.H., B.F., T.M., F.V., F.A.A., and S.C. coordinated field sampling. All authors contributed to the field sampling. C.M. and M.B. compiled and assembled *in situ* measurements in the GnpIS database. B.D. extracted climatic and topographic data and derived environmental indices for all the sampling sites. R.B., L.O., B.Da., P.A., and C.M. curated data, checked quality, and prepared the datasets with metadata descriptions for sharing and potential reuse. L.O., K.H., B.Da., S.C., and B.F. wrote the manuscript. B.F. coordinated GenTree. All authors commented on an earlier version and approved the final version of the manuscript.

## Supplementary Material

giab010_GIGA-D-20-00189_Original_Submission

giab010_GIGA-D-20-00189_Revision_1

giab010_GIGA-D-20-00189_Revision_2

giab010_Response_to_Reviewer_Comments_Original_Submission

giab010_Response_to_Reviewer_Comments_Revision_1

giab010_Reviewer_1_Report_Original_Submission Greg Guerin -- 8/16/2020 Reviewed

giab010_Reviewer_2_Report_Original_Submission Felipe Bravo -- 9/6/2020 Reviewed
