# Supplementary material for: The GenTree Platform: growth traits and tree-level environmental data in 12 European forest tree species
Source: Gigascience. 2021 Mar 18;10(3):giab010. doi: 10.1093/gigascience/giab010 (PMC7970660; doi:10.1093/gigascience/giab010)
Supplement: giab010_GIGA-D-20-00189_Original_Submission [file giab010_giga-d-20-00189_original_submission.pdf]

GigaScience

The GenTree Platform: growth traits and tree-level environmental data in twelve European forest tree species

--Manuscript Draft--

**Manuscript Number:**	GIGA-D-20-00189
**Full Title:**	The GenTree Platform: growth traits and tree-level environmental data in twelve European forest tree species
**Article Type:**	Data Note
**Funding Information:**	Horizon 2020 Framework Programme (676876) — Mr. Bruno Fady<br>Staatssekretariat für Bildung, Forschung und Innovation (6.0032) — Mr. Felix Gugerli
**Abstract:**	Background<br><br>Progress in the field of evolutionary forest ecology has been hampered by the huge challenge of phenotyping trees across their ranges in their natural environments, and the limitation in high-resolution environmental information.<br><br>Findings<br><br>The GenTree Platform contains phenotypic and environmental data from 4959 trees from twelve ecologically and economically important European forest tree species: Abies alba Mill. (Silver fir), Betula pendula Roth. (silver birch), Fagus sylvatica L. (European beech), Picea abies (L.) H. Karst (Norway spruce), Pinus cembra L. (Swiss stone pine), Pinus halepensis Mill. (Aleppo pine), Pinus nigra Arnold (European black pine), Pinus pinaster Aiton (maritime pine), Pinus sylvestris L.(Scots pine), Populus nigra L.(European black poplar), Taxus baccata L.(English yew), and Quercus petraea (Matt.) Liebl.(sessile oak). Phenotypic (height, DBH, crown-size, bark-thickness, biomass, straightness, forking, branch angle, fructification), regeneration, environmental in-situ measurements (soil depth, vegetation cover, competition indices), and environmental modeling data (precipitation, temperature, insolation, drought indices) were obtained from trees in 194 sites covering the species' geographic ranges and reflecting local environmental gradients.<br><br>Conclusion<br><br>The GenTree Platform is a new resource for investigating ecological and evolutionary processes in forest trees. The coherent phenotyping and environmental characterization across 12 species in their European ranges allows for a wide range of analyses from forest ecologists, conservationists and macroecologists. In addition, the data here presented can be linked to the GenTree Dendroecological collection, the GenTree Leaf Trait collection, and the GenTree Genomic collection presented elsewhere, that together build the largest evolutionary forest ecology data collection available.
**Corresponding Author:**	Lars Opgenoorth<br>Philipps-Universität Marburg<br>Marburg, Hessen GERMANY
**Corresponding Author Secondary Information:**	
**Corresponding Author's Institution:**	Philipps-Universität Marburg
**Corresponding Author's Secondary Institution:**	
**First Author:**	Lars Opgenoorth
**First Author Secondary Information:**	

Powered by Editorial Manager® and ProduXion Manager® from Aries Systems Corporation

**Order of Authors:**	Lars Opgenoorth
	Benjamin Dauphin
	Raquel Benavides
	Katrin Heer
	Paraskevi Alizoti
	Elisabet Martínez-Sancho
	Ricardo Alía
	Olivier Ambrosio
	Albet Audrey
	Camilla Avanzi
	Evangelia Avramidou
	Francesca Bagnoli
	Evangelos Barbas
	Cristina C. Bastias
	Catherine Bastien
	Eduardo Ballesteros
	Giorgia Beffa
	Frédéric Bernier
	Henri Bignalet
	Guillaume Bodineau
	Damien Bouic
	Sabine Brodbeck
	William Brunetto
	Jurata Buchovska
	Melanie Buy
	Ana M. Cabanillas-Saldaña
	Bárbara Carvalho
	Nicolas Cheval
	José M. Climent
	Marianne Correard
	Eva Cremer
	Darius Danusevičius
	Fernando Del Caño
	Jean-Luc Denou
	Nicolas di Gerardi
	Bernard Dokhelar
	Alexis Ducousso
	Eskild Nilsen
	Anna-Maria Farsakoglou
	Patrick Fonti
	Ioannis Ganopoulos

Powered by Editorial Manager® and ProduXion Manager® from Aries Systems Corporation

	José M. García del Barrio
	Olivier Gilg
	Santiago Gonzalez-Martinez
	René Graf
	Alan Gray
	Delphine Grivet
	Felix Gugerli
	Christoph Hartleitner
	Enja Hollenbach
	Agathe Hurel
	Bernard Issehut
	Florence Jean
	Veronique Jorge
	Arnaud Jouineau
	Jan-Philipp Kappner
	Katri Kärkkäinen
	Robert Kesälahti
	Florian Knutzen
	Sonja Kujala
	Timo Kumpula
	Mariaceleste Labriola
	Celine Lalanne
	Johannes Lambertz
	Martin Lascoux
	Vincent Lejeune
	Gregoire Le-Provost
	Joseph Levillain
	Mirko Liesebach
	David López-Quiroga
	Benjamin Meier
	Ermioni Malliarou
	Jérémy Marchon
	Nicolas Mariotte
	Antonio Mas
	Silvia Matesanz
	Helge Meischner
	Célia Michotey
	Pascal Milesi
	Sandro Morganti
	Daniel Nievergelt
	Eduardo Notivol

Powered by Editorial Manager® and ProduXion Manager® from Aries Systems Corporation

	Geir Ostreng
	Birte Pakull
	Annika Perry
	Andrea Piotti
	Christophe Plomion
	Nicolas Poinot
	Mehdi Pringarbe
	Luc Puzos
	Tanja Pyhäjärvi
	Annie Raffin
	José A. Ramírez-Valiente
	Christian Rellstab
	Dourthe Remi
	Sebastian Richter
	Juan J. Robledo-Arnuncio
	Sergio San Segundo
	Outi Savolainen
	Volker Schneck
	Ivan Scotti
	Vladimir Semerikov
	Lenka Slámová
	Jørn Henrik Sønstebø
	Ilaria Spanu
	Jean Thevenet
	Mari Mette Tollefsrud
	Norbert Turion
	Giovanni Giuseppe Vendramin
	Marc Villar
	Georg Von Arx
	Johan Westin
	Bruno Fady
	Tor Myking
	Fernando Valladares
	Filippos A. Aravanopoulos
	Stephen Cavers
**Order of Authors Secondary Information:**	
**Additional Information:**	
**Question**	**Response**
Are you submitting this manuscript to a special series or article collection?	No

**Experimental design and statistics**<br><br>Full details of the experimental design and statistical methods used should be given in the Methods section, as detailed in our Minimum Standards Reporting Checklist. Information essential to interpreting the data presented should be made available in the figure legends.<br><br>Have you included all the information requested in your manuscript?	Yes
**Resources**<br><br>A description of all resources used, including antibodies, cell lines, animals and software tools, with enough information to allow them to be uniquely identified, should be included in the Methods section. Authors are strongly encouraged to cite Research Resource Identifiers (RRIDs) for antibodies, model organisms and tools, where possible.<br><br>Have you included the information requested as detailed in our Minimum Standards Reporting Checklist?	Yes
**Availability of data and materials**<br><br>All datasets and code on which the conclusions of the paper rely must be either included in your submission or deposited in publicly available repositories (where available and ethically appropriate), referencing such data using a unique identifier in the references and in the “Availability of Data and Materials” section of your manuscript.<br><br>Have you have met the above requirement as detailed in our Minimum Standards Reporting Checklist?	Yes

Click here to access/download;Manuscript;02_Publications_Opgenoorth et

The GenTree Platform: growth traits and tree-level environmental data in twelve European forest tree species

Opgenoorth, Lars[1,2], Dauphin, Benjamin[2], Benavides, Raquel[3], Heer, Katrin[1], Alizoti, Paraskevi[4], Martínez-Sancho, Elisabet[2], Alía, Ricardo[5], Ambrosio, Olivier[6], Audrey, Albet[7], Auñón, Francisco[5], Avanzi, Camilla[8], Avramidou, Evangelia[4], Bagnoli, Francesca[8], Barbas, Evangelos[4], Bastias, Cristina C.[9], Bastien, Catherine[10], Ballesteros, Eduardo[5], Beffa, Giorgia[2], Bernier, Frédéric[7], Bignalet, Henri[7], Bodineau, Guillaume[11], Bouic, Damien[7], Brodbeck, Sabine[2], Brunetto, William[6], Buchovska, Jurata[12], Buy, Melanie[6], Cabanillas-Saldaña, Ana M.[13], Carvalho, Bárbara[3], Cheval, Nicolas[7], Climent, José M.[5], Correard, Marianne[6], Cremer, Eva[14], Danusevičius, Darius[12], Del Caño, Fernando[5], Denou, Jean-Luc[7], di Gerardi, Nicolas[2], Dokhelar, Bernard[7], Ducousso, Alexis[15], Eskild Nilsen, Anne[16], Farsakoglou, Anna-Maria[4], Fonti, Patrick[2], Ganopoulos, Ioannis[17], García del Barrio, José M.[5], Gilg, Olivier[6], Gonzalez-Martinez, Santiago[7], Graf, René[2], Gray, Alan[18], Grivet, Delphine[5], Gugerli, Felix[2], Hartleitner, Christoph[19], Hollenbach, Enja[1], Hurel, Agathe[7], Issehut, Bernard[7], Jean, Florence[6], Jorge, Veronique[20], Jouineau, Arnaud[6], Kappner, Jan-Philipp[1], Kärkkäinen, Katri[21], Kesälahti, Robert[22], Knutzen, Florian[14], Kujala, Sonja T[21], Kumpula, Timo A[22], Labriola, Mariaceleste[8], Lalanne, Celine[15], Lambertz, Johannes[1], Lascoux, Martin[23], Lejeune, Vincent[11], Le-Provost, Gregoire[15], Levillain, Joseph[24], Liesebach, Mirko[25], López-Quiroga, David[3], Meier, Benjamin[2], Malliarou, Ermioni[4], Marchon, Jérémy[2], Mariotte, Nicolas[6], Mas, Antonio[3], Matesanz, Silvia[26], Meischner, Helge[1], Michotey, Célia[27], Milesi, Pascal[28], Morganti, Sandro[2], Nievergelt, Daniel[2], Notivol, Eduardo[29], Ostreng, Geir[16], Pakull, Birte[25], Perry, Annika[18], Piotti, Andrea[8], Plomion, Christophe[15], Poinot, Nicolas[7], Pringarbe, Mehdi[6], Puzos, Luc[7], Pyhäjärvi, Tanja[22], Raffin, Annie[7], Ramírez-Valiente, José A.[5], Rellstab, Christian[2], Remi, Dourthe[7], Richter, Sebastian[1], Robledo-Arnuncio, Juan J.[5], San Segundo, Sergio[5], Savolainen,Outi[22], Schneck,Volker[30], Scotti, Ivan[6], Semerikov, Vladimir[31], Slámová, Lenka[2], Sønstebø, Jørn Henrik[16], Spanu, Ilaria[8], Thevenet, Jean[6], Tollefsrud, Mari Mette[16], Turion, Norbert[6], Vendramin, Giovanni Giuseppe[8], Villar, Marc[20], Von Arx, Georg[2], Westin, Johan[32], Fady,Bruno[6], Myking, Tor[16], Valladares, Fernando[3], Aravanopoulos, Filippos A.[4], Cavers, Stephen[18]

[1]Philipps University Marburg, Faculty of Biology, Karl-von-Frisch Strasse 8, 35043 Marburg, Germany

[2]Swiss Federal Research Institute WSL, Zu̐rcherstrasse 111, 8903, Birmensdorf, Switzerland

[3]LINCGlobal, Department of Biogeography and Global Change, Museo Nacional de Ciencias Naturales, CSIC,Serrano 115 dpdo, 28006, Madrid, Spain

[4]Aristotle University of Thessaloniki, School of Forestry and Natural Environment, Laboratory of Forest Genetics and Tree Improvement, 54124 Thessaloniki, Greece

[5]Instituto Nacional de Investigación y Tecnología Agraria y Alimentaria - Centro de Investigación Forestal (INIA-CIFOR), Ctra. de la Coruña km 7.5, 28040, Madrid, Spain

[6]Institut National de Recherche en Agriculture, Alimentation et Environment (INRAE), Domaine Saint Paul, Site Agroparc, 84914, Avignon, France

[7]Institut National de Recherche en Agriculture, Alimentation et Environment (INRAE), UEFP, 33610 Cestas, France

[8]Institute of Biosciences and BioResources, National Research Council (CNR), via Madonna del Piano 10, 50019, Sesto, Fiorentino, Italy

[9]Centre d'Ecologie Fonctionnelle et Evolutive (CEFE), CNRS, UMR 5175, Montpellier, France

[10]Institut National de Recherche en Agriculture, Alimentation et Environment (INRAE), Dept ECOFA, F-45075 Orléans, France

[11]Institut National de Recherche en Agriculture, Alimentation et Environment (INRAE), GBFOR, 45075 Orléans, France

[12]Vytautas Magnus University, Studentu Street 11, 53361, Akademija, Lithuania.

[13]Departamento de Agricultura, Ganadería y Medio Ambiente, Gobierno de Aragón, P. Mª Agustín 36, 50071 Zaragoza, Spain

[14]Bavarian Institute for Forest Genetics , Forstamtsplatz 1, 83317 Teisendorf, Germany

[15]INRAE, Univ. Bordeaux, BIOGECO, F-33770 Cestas, France
[16]Division of Forestry and Forest Resources, Norwegian Institute of Bioeconomy Research (NIBIO), P.O. Box 115, 1431, Ås, Norway
[17]Institute of Plant Breeding and Genetic Resources, Hellenic Agricultural Organization DEMETER (ex NAGREF), Thermi, 57001, Greece
[18]UK Centre for Ecology and Hydrology, Bush Estate Penicuik, EH26 0QB, United Kingdom
[19]LIECO GmbH & Co KG
[20]Institut National de Recherche en Agriculture, Alimentation et Environment (INRAE), ONF, BIOFORA, 45075 Orléans, France
[21]Natural Resources Institute Finland, Paavo Havaksentie 3, 90014, University of Oulu, Finland
[22]University of Oulu, Pentti Kaiteran katu 1, 90014, University of Oulu, Finland
[23]Department of Ecology & Genetics, EBC, Uppsala University, Norbyvägen 18D, 75236, Uppsala, Sweden
[24]Université de Lorraine, AgroParisTech, INRAE, SILVA, 54000 Nancy, France
[25]Thünen Institute of Forest Genetics, Sieker Landstr. 2, Grosshansdorf, 22927, Germany
[26]Área de Biodiversidad y Conservación, Universidad Rey Juan Carlos, Calle Tulipán s/n, 28933, Móstoles, Spain
[27]Institut National de Recherche en Agriculture, Alimentation et Environment (INRAE), URGI, Versailles, France
[28]Department of Ecology & Genetics, EBC, Science for Life Laboratory, Uppsala University, Uppsala, Sweden
[29]Centro de Investigación y Tecnología Agroalimentaria de Aragón - Unidad de Recursos Forestales (CITA),Avda. Montañana 930, 50059, Zaragoza, Spain
[30]Thünen Institute of Forest Genetics, Eberswalder Chaussee 3a, Waldsieversdorf, 15377, Germany
[31]Institute of Plant and Animal Ecology, Ural branch of RAS, 8 Marta St. 202, 620144, Ekaterinburg, Russia
[32]Skogforsk, Tomterna 1, 918 21 Sävar, Sweden

opgenoorth@uni-marburg.de;lars.opgenoorth@wsl.ch
benjamin.dauphin@wsl.ch
rbenavidescalvo@gmail.com
katrin.heer@uni-marburg.de
alizotp@for.auth.gr
elisabet.martinez@wsl.ch
alia@inia.es
olivier.ambrosio@inrae.fr
audrey.albet.1@inrae.fr
aunon.francisco@inia.es
camilla.avanzi@ibbr.cnr.it
aevaggelia@yahoo.com
francesca.bagnoli@ibbr.cnr.it
vbarbas@for.auth.gr
crbasc@gmail.com
catherine.bastien@inrae.fr
eduardo.ballesteros@inia.es
giorgia.beffa@gmail.com
frederic.bernier@inrae.fr
henri.bignalet@inrae.fr
guillaume.bodineau@inrae.fr
sabine.brodbeck@wsl.ch
william.brunetto@inrae.fr
jurata.buchovska@vdu.lt

Melanie.Buy@inrae.fr
acabanillas@aragon.es
babic.barbosa@gmail.com
nicolas.cheval@inrae.fr
climent@inia.es
marianne.correard@inrae.fr
Eva.Cremer@awg.bayern.de
damien.bouic@inrae.fr
darius.danusevicius@vdu.lt
umgf@inia.es
jean-luc.denou@inrae.fr
nicolas.degirardi@gmail.com
bernard.dokhelar@inrae.fr
alexis.ducousso@inrae.fr
anne.eskild.nilsen@nibio.no
amfarsakoglou@gmail.com
patrick.fonti@wsl.ch
giannis.ganopoulos@gmail.com
jmgarcia@inia.es
olivier.gilg@inrae.fr
santiago.gonzalez-martinez@inrae.fr
rene.graf@wsl.ch
alangray@ceh.ac.uk
dgrivet@inia.es
felix.gugerli@wsl.ch
hartleitner@sfl.at
Hollenb4@students.uni-marburg.de
agathe.hurel@inrae.fr
bernard.issenhuth@inrae.fr
florence.jean@inrae.fr
veronique.jorge@inrae.fr
arnaud.jouineau@inrae.fr
kappnerj@students.uni-marburg.de
katri.karkkainen@luke.fi
robert.kesalahti@oulu.fi
FAKnutzen@gmail.com
sonja.kujala@luke.fi
timo.kumpula@oulu.fi
mclabriola@gmail.com
celine.lalanne@inrae.fr
Lambertj@students.uni-marburg.de
martin.lascoux@ebc.uu.se
vincent.lejeune@inrae.fr
gregoire.le-provost@inrae.fr
joseph.levillain@inrae.fr
mirko.liesebach@thuenen.de
david.quiroga@csic.es
meier.benjamin@outlook.com
ermionimalliarou@yahoo.gr
jeremy.marchon@romandie.ch
nicolas.mariotte@inrae.fr
antonio.mas@mncn.csic.es

silvia.matesanzgarcia@gmail.com
helgem@posteo.de
celia.michotey@inrae.fr
pascal.milesi@scilifelab.uu.se
msandro@posteo.ch
daniel.nievergelt@wsl.ch
enotivol@cita-aragon.es
geir.ostreng@nibio.no
birte.pakull@thuenen.de
annt@ceh.ac.uk
andrea.piotti@ibbr.cnr.it
christophe.plomion@inrae.fr
nicolas.poinot@inrae.fr
mehdi.pringarbe@inrae.fr
luc.puzos@inrae.fr
tanja.pyhajarvi@oulu.fi
annie.raffin@inrae.fr
josealberto.ramirezvaliente@gmail.com
christian.rellstab@wsl.ch
remi.dourthe@inrae.fr
richte3d@staff.uni-marburg.de
jjrobledo@gmail.com
sergio.sansegundo@inia.es
outi.savolainen@oulu.fi
volker.schneck@thuenen.de
ivan.scotti@inrae.fr
semerikov@ipae.uran.ru
lenka.mateju@wsl.ch
jorn.sonstebo@nmbu.no
ilaria.spanu@ibbr.cnr.it
jean.thevenet@inrae.fr
Mari.Mette.Tollefsrud@nibio.no
Norbert.Turion@inrae.fr
giovanni.vendramin@ibbr.cnr.it
marc.villar@inrae.fr
georg.vonarx@wsl.ch
johan.westin@skogforsk.se
bruno.fady@inrae.fr
tor.myking@nibio.no
valladares@ccma.csic.es
aravanop@auth.gr
scav@ceh.ac.uk

Corresponding authors: opgenoorth@uni-marburg.de, benjamin.dauphin@bluewin.ch, scav@ceh.ac.uk

Abstract

Background:
Progress in the field of evolutionary forest ecology has been hampered by the huge challenge of phenotyping trees across their ranges in their natural environments, and the limitation in high-resolution environmental information.
Findings:
The GenTree Platform contains phenotypic and environmental data from 4959 trees from twelve ecologically and economically important European forest tree species: *Abies alba* Mill. (Silver fir), *Betula pendula* Roth. (silver birch), *Fagus sylvatica* L. (European beech), *Picea abies* (L.) H. Karst (Norway spruce), *Pinus cembra* L. (Swiss stone pine), *Pinus halepensis* Mill. (Aleppo pine), *Pinus nigra* Arnold (European black pine), *Pinus pinaster* Aiton (maritime pine), *Pinus sylvestris* L.(Scots pine), *Populus nigra* L.(European black poplar), *Taxus baccata* L.(English yew), and *Quercus petraea* (Matt.) Liebl.(sessile oak). Phenotypic (height, DBH, crown-size, bark-thickness, biomass, straightness, forking, branch angle, fructification), regeneration, environmental in-situ measurements (soil depth, vegetation cover, competition indices), and environmental modeling data (precipitation, temperature, insolation, drought indices) were obtained from trees in 194 sites covering the species' geographic ranges and reflecting local environmental gradients.
Conclusion:
The GenTree Platform is a new resource for investigating ecological and evolutionary processes in forest trees. The coherent phenotyping and environmental characterization across 12 species in their European ranges allows for a wide range of analyses from forest ecologists, conservationists and macroecologists. In addition, the data here presented can be linked to the GenTree Dendroecological collection, the GenTree Leaf Trait collection, and the GenTree Genomic collection presented elsewhere, that together build the largest evolutionary forest ecology data collection available.

Measurements	vegetation cover • rock cover • soil depth • competition index • regeneration • dbh • height • crown size • bark thickness • number of fruits • stem straightness • branch angle • forking index
Technology Type(s)	bark gauges • calculations • calipar • clinometer • gps device • increment corer • laser • telescopic measuring pole
Factor Type(s)	tree species
Sample Characteristic Organism	Abies alba • Betula pendula • Fagus sylvatica • Picea abies • Pinus cembra • Pinus halepensis • Pinus nigra • Pinus pinaster • Pinus sylvestris • Populus nigra • Taxus baccata • Quercus petraea
Sample Characteristic Location	Europe

Machine-accessible metadata file describing the reported data:

Keywords

Regeneration, dbh, height, crown-size, bark-thickness, fruit-number, stem-straightness, branch-angle, forking-index, soil-depth

Context

The impacts of climate change and land-use change on forests are expected to be acute, altering distribution ranges and ecosystem functioning, as well as the interactions among species [1]. Forecasts indicate that near-surface temperature will shift poleward at mean rates of 80-430 m yr^{-1} for temperate forests during the 21st century [2]. This translates into northward shifts of trees' bioclimatic envelopes from 300 to 800 km within one century [2]. More importantly, the frequency and intensity of drought events, heat waves, forest fires and pest outbreaks [3] are expected to increase. Such events have been witnessed in recent years, for example with massive tree mortality in central Europe following the 2018 heatwave [4].

In the light of these changes, species and forest ecosystem resilience will depend on the extent and structure of genetic variation and adaptive potential. From results of extensive networks of field experiments (provenance trials), it has long been shown that tree species are locally adapted at multiple spatial scales. In Europe, where most tree populations have established following post-glacial recolonization, such patterns of local adaptation must have developed rapidly and despite long generation time [5], a process enabled by high levels of within-population plasticity, genetic and epigenetic variation, large population sizes and extensive gene flow [6]. Recent work has shown that genetic variation for stress response may be strongly structured along environmental gradients, such as water availability [7], temperature [8], or photoperiod [9]. However, the spatial patterns of current adaptation in particular traits are only partly informative regarding the potential for future adaptation under a changing climate. To advance our understanding of the adaptive potential of trees, it is crucial to evaluate multiple traits in parallel to be able to model their putative response to new environmental conditions.

Recently, substantial effort has been made to identify specific genes and gene combinations that have undergone selection, by associating mutations at candidate loci with phenotypes related to stress events[10,11] or with environmental variables [12]. This latter example by Yeaman and coworkers[12] is one of the first association studies in forest tree species on a large genomic scale and the first to investigate convergent local adaptation in distantly related tree species. However, progress in this field has been hampered by limited genomic resources, the lack of small-scale, high-resolution environmental information [13], and the huge challenge of phenotyping trees in their natural environments[14,15].

The GenTree Platform aims to address these challenges by providing individual level, high-resolution phenotypic and environmental data for a set of up to 20 sampling sites for each of twelve ecologically and economically important forest tree species across Europe. For a subset of seven species (*B. pendula*, *F. sylvatica*, *P. abies, P. pinaster*, *P. sylvestris*, *Populus nigra*, and *Q.petraea*), the sampling sites were paired, i.e. contained two stands that were close enough to be connected by gene flow but located in contrasting environments. The sampling design described here was used for collecting phenotypic trait and ecological data. In addition, tree ring and wood density measures for the same trees were assessed [16], and datasets on leaf traits, including specific leaf area (SLA) and isotopic content, and high-resolution SNP genotypes for each tree, were established. All data and metadata are kept in the GnpIS repository (https://urgi.versailles.inra.fr/ephesis/ephesis/viewer.do#dataResults/trialSetIds=27) which makes updates possible.[17]

Methods

All recorded parameters are listed in Table 1.

Sampling strategy

To optimize the sampling design for genome scans and association studies, we followed the recent theoretical work by Lotterhos and Whitlock [18,19], which indicates that paired sampling designs have more power to detect the genomic signature of local adaptation. Using this framework, populations from across the natural range of a species are sampled in pairs, with the two sites in each pair located geographically close enough to be genetically similar at neutral genes due to a common evolutionary history and ongoing gene flow, but in distinct selective niches such that the local fitness optimum differs between the two sites. This sampling confers more power to detect evidence of selection in the genome through either association with environmental or phenotypic variables or the detection of outliers (e.g. for genetic differentiation, F_{ST}) (ibid.). Trees are very amenable to a pairwise approach since they are known to be locally adapted, often at fine spatial scales [20,21] and irrespective of gene flow distances[6]. This strategy was followed for a subset of seven species (see above) for which genomic resources were available (full or draft genome).

Such local niche contrasts are neither easy to identify nor readily available when environments are very homogenous. Therefore, a second principle of the sampling design was to cover a large part of each species' natural geographic range (Fig. 1) and environmental space (Fig. 2) to increase selective niche variation. Finally, sites with a history of intensive management or any other intense and obvious anthropogenic or natural disturbances were avoided. This strategy was followed for all 12 species.

Selection of trees on sites

A minimum of 25 trees were sampled per site to capture the natural phenotypic and genetic variability. Trees had to be mature but not senescent, dominant or codominant, and had to show no signs of significant damage due to pests and diseases or generally low vigor. Sampled trees were at least 30 meters apart and, where possible, were chosen along several parallel linear transects across each site, typically resulting in 2-4 transects per sampling site to keep the overall sampling area below 3 hectares.

Site and tree metadata

Sites were labeled by a two-letter country code (ISO 3166-1 alpha-2) followed by a two-letter species code and a two-digit site number (Table 1). Individual tree labels added another two-digit tree number. Every tree was permanently labeled for potential subsequent additional phenotyping or sampling.

Handheld GPS devices were used to record the position of each tree. The precision of GPS measurements in forests is notoriously challenging: regular commercial devices achieve an accuracy of about 8-15 m with good satellite coverage. Given that trees were selected with a minimum distance of 30 m this accuracy was sufficient for the correct positioning of trees relative to each other. An overall population position was defined by taking the mean value across all the individual tree measurements. Coordinates were in decimal degrees with 4 decimal units to reflect the general measurement accuracy (~11.1 m) and were stored in the WGS 84 reference system. GPS devices were also used to record the tree's elevation, either directly or through posthoc positioning in digital elevation models. The local aspect at the site of the tree was measured by compass in five-degree steps in the direction of the steepest slope.

The Metadata for each site consists of an ID code (see above), sampling date, location (GPS coordinates, see above) and elevation in meters above sea level (m a.s.l). Each stand was also characterized as being monospecific or mixed (in the latter case the most common co-occurring species was noted), stand structure was noted as single or multiple layered, and the age distribution as even or uneven (categorical variables).

Competition index at tree level
Competition indices were calculated following Canham et al[22] and Lorimer et al[23]. Specifically, the distance-dependent competition index (NCI) following Canham et al [22] was computed as $NCI = \sum_{i=1}^{5}({dbh_i}/{dist_i})$ where dbh_i is the diameter at breast height of competitor tree *i*, and $dist_i$ is the distance between the subject tree and competitor tree *i*. This index assumes that the net effects of neighboring trees vary as a direct function of the size of the neighbors and as an inverse function of the distance. For this purpose, the distance to the five nearest neighbors of each target tree was measured and their respective diameter at breast height was measured.

The second index following Lorimer[23] was calculated as $NCI = \sum_{i=1}^{5}({dbh_i}/{dbh_i})/dist_i$ that follows the same notation as above, and where dbh_i is the dbh of the subject tree.

Moreover, it was noted whether competitor trees were conspecific to the target tree or not. Each multi-stemmed tree was considered as a single competitor where each stem larger than 15 cm was measured and added to the sum of means.

Environmental characteristics within subplots around each tree
Surrounding each target tree, slope, vegetation cover, and stone content were assessed in a 10 m x 10 m plot. The slope was assessed using a clinometer. Vegetation and rock cover were estimated in the classes <5%, 5-20%, 20-40%, 40-60%, 60-80%, 80-95%. Soil depth was estimated at three random points in the quadrat to a maximum of 60 cm with a pike and was averaged across these three values.

Regeneration
In the same 10 m x 10 m plots, natural regeneration of the target species was assessed according to the following four classes: absent (no recruit visible), scattered (few/scattered individuals), groups (presence of scattered groups within the plot), and abundant (regularly spread all over the plot) and is indicated in the data base with values from 1-4. As this method cannot resolve paternity, the results indicate realized fecundity at the stand level.

Tree phenotypes

DBH (cm)
DBH was measured at a stem height of 1.3 m using either a caliper by measuring two perpendicular diameters and subsequently taking the average or by measuring the circumference of the tree using a tape and computing the diameter from that value. Each measurement was performed to the nearest 0.1 cm. If a tree had more than one trunk, all of them were measured and the average was recorded.

Height (m)
Height from ground to the top of the crown was measured using a hypsometer (Nikon forestry Pro Laser), a laser vertex (Haglof Vertex III, Langsele, Sweden) or a Laser Range Meter (Bosch GLM 50 C, Leinfelden-Echterdingen, Germany). For short trees a telescopic measuring pole was used. Height was noted to the nearest 0.1 m.

Crown size (m²)
Crown size was measured as the circular and ellipsoid plane area of the crown. For this we measured two perpendicular crown diameters (canopy 1 and 2) by using a measurement tape, with the first measurement being made along the longest axis of the crown, from one edge to the other, and by visually projecting the crown margin onto the ground to the nearest

decimeter. For the ellipse area we calculated $\left({d_i}/{2}\right) * \left({d_j}/{2}\right) * \pi$ and for the circular area $(d_i + d_j)/2 * \pi$.

Bark thickness (mm)
For measuring bark thickness, we used bark gauges (Haglof Barktax) or a tape after extracting the bark with a small caliper (if bark could be detached without tree damage) or dendro corers (Haglof increment borer) in case of strong and thick bark. Five measurements were taken for each tree at breast height and the average was calculated. For tree species with a clear dichotomy of bark thickness (e.g. old *Populus nigra*, *T. baccata*), we included at least two measurements from the thinner and thicker bark areas each.

Number of fruits (units)
In conifers, cones were counted by providing the average of three rounds of counting, made by an observer on the ground using binoculars. Only mature (brown) and closed cones were counted, i.e., those containing seeds, and not immature (green) or open cones, whose seeds had already been dispersed (open cones often stay on the branch for several years after seeds are dispersed). In broadleaves, the number of fruits was counted for 30 seconds, repeating the procedure three times to then average the three counts.

In case of species with very small fruits that are hard to see individually and in locations with a very limited view to the canopy, each tree was assigned to one of five categories, namely 0 (no fruits), 1 (a few fruits in a small section of the crown), 2 (a few fruits in two or more sections of the crown), 3 (a lot of fruits in a small section of the crown), and 4 (a lot of fruits in two or more sections of the crown).

Straightness
Straightness of the stem was classified according to five levels: (1) No straight stem, (2) moderate or strong bends, (3) slight to moderate bend in different directions, (4) fairly straight (in one direction slightly crooked), (5) absolutely straight. This was performed on the lower 15 m of the tree beginning from the ground with the crown not taken into account. In the case of forked stems, only the trunk below the deepest forking point was evaluated.

Branch angle
Branch angle was classified at two successive whorls according to a five-scale scheme in conifers with (1) <23°, (2) 23-45°, (3) 45-67°, (4) 67°-90°, (5) > 90°, and a four-scale scheme in broadleaves omitting the >90° class. In black poplar, only the top two meters of the crown were considered.

Forking index
The branching of a tree in two (fork) or more (ramicorn) equally thick and long stems was assessed with a forking index. The index took into account two parameters. First a score for the relative position of the fork: (4) no forking, (3) forking in the upper third of the tree, (2) forking in the middle third of the tree, (1) forking in the lower third of the tree; and second the number of axes (stems). The score of the relative position was then multiplied by ten and divided by the number of axes.

Modelled environmental data extracted for GenTree sites

Topography, soil, and climate data were compiled to characterize environmental conditions in each GenTree sampling site as follows.

Topography
We used the European digital elevation model to describe topographic conditions at 25 m spatial resolution with a vertical accuracy of about ± 7 meters (EU-DEM v. 1.1 from the Copernicus program; https://land.copernicus.eu/). We derived 14 variables (Table 2) based on biological hypotheses and their informative power at the local scale [24]. We calculated morphometric, hydrologic, and radiation grids for each GenTree site and visually inspected data integrity using SAGA 6.2 (details in Table 2;[25]).

Soil
We collected available data on water capacity at seven soil depths using SoilGrids250m [26]. We estimated Pearson's correlation coefficients, *r*, between soil layers and then averaged the four first superficial (0, 5, 15, and 30 cm) and the three deeper (60, 100, and 200 cm) layers that were highly correlated, respectively.

Climate
We extracted climate data with high spatial resolution (30 arcsec) using CHELSA v. 1.2 [27]. CHELSA is based on a quasi-mechanistic statistical downscaling global reanalysis and global circulation model that, in particular, reliably interpolates the amount of precipitation using an orographic rainfall and wind effect. The dataset consisted of 48 climatic, 19 bioclimatic, four drought- and two frost-related variables for the reference period 1979-2013 (Table 2; http://chelsa-climate.org/bioclim/). We extracted all modeled environmental values for each individually geo-referenced tree using SAGA 6.2.

Data Records

The data presented are structured in four independent csv files that can be merged using the site identifier (m04.site.id) or tree identifier (m06.tree.id). The same codes can be used to merge additional data namely from the GenTree Dendroecological Collection[16], the GenTree LeafTrait Collection, and the GenTree Genomic Collection (personal communication). The first file contains the metadata descriptions, the second the individual phenotypic traits, the third the environmental data, and the fourth the modelled environmental data. These data files are available at figshare data repository (https://figshare.com/s/4d57474fd63864a6dfd8). The data are indexed in Table 2.

Table 1 Variables names, explanations, and specifications measured for all 4959 trees and all 194 GenTree sites.

**Variable name**	**Variable explanation**	**Specification**
	**GenTree Platform metadata**	
m01.spec	Species abbreviations	*Abies Alba* [AA], *Betula pendula* [BP], *Fagus sylvatica* [FS], *Picea abies* [PA], *Pinus cembra* [PC], *Pinus halepensis* [PH], *Pinus nigra* [PN], *Populus nigra* [PO], *Pinus pinaster* [PP], *Pinus sylvestris* [PS], *Quercus petraea* [QP], *Taxus baccata* [TB]
m02.country	Country abbreviations	Isocode 6133-2; Austria [AT], Switzerland [CH], Germany [DE], Spain [ES], Finland [FI], France [FR], Great Britain [GB], Greece [GR], Italy [IT], Lithuania [LT], Norway [NO], Sweden [SE]
m03.site.num	Site numbers	Running numbers of sites per species 01-24
m04.site.id	Complete site-ID per species	Merger of m01-m03
m05.tree.num	Tree numbers	Running numbers within sites 01-25
m06.tree.id	Complete tree ID	Merger of m01-m03,m05
m07.trial.name	Site name	
m08.lat	Latitude	Decimal degrees, WGS84
m09.lon	Longitude	Decimal degrees, WGS84
	**GenTree Platform phenotypes**	
p01.height	Height	m
p02.dbh	DBH	cm

p03.bark	Bark thickness mean	mean value of bark thickness, cm
p04.trunk	Trunk straightness/flexuosity	5: absolutely straight, 4: fairly straight (in one direction slightly crooked), 3: slight to moderate bend in different directions, 2: moderate or strong bends, 1: no straight stem
p05.branch	Branch angle	1: <23° (steep), 2: 23° - 45°, 3: 45º-67º, 4: 67º-90º (plain), 5: >90º
p06.fork	Forking index	1: fork at the lower third of tree height, 2: fork at middle third, 3: fork at upper third, 4: no fork – multiplied by 10 and then divided by the number of stems
p07.canopy.1	Canopy projection REP 1	Crown diameter projection in m
p08.canopy.2	Canopy projection REP 2	Crown diameter projection in m
p09.crown.ellipse	Crown ellipse	Area of an ellipse (d1/2)*(d2/2)*π in m^2
p10.crown.round	Crown size	As some only have one diameter, round areas with the mean diameter ((D1+D2/2)/2)^2*π in m^2
p11.regeneration	Natural regeneration	1: absent, 2: scattered, 3: groups, 4: abundant
p12.fruit.mean	Fruit/cone number	Fruit_notation: fructification note
p13.basal.area		
**GenTree Platform in-situ environmental measurements**		
e01.plant.cover	Total plant cover	1: none, 2: little (5-20%), 3: low (20-40%), 4: medium (40-60%), 5: high (60-80%), 6: very high(80-95%), 7: full cover (>95%)
e02.comp.index.a	Competition index A	CI assessed following Canham et al 2004, and multistems as the sum
e03.comp.index.b	Competition index B	CI assessed following Canham et al 2004, and multistems assessing the sum of basal areas and then the dbh
e04.comp.index.c	Competition index C	CI assessed following Lorimer 1983, and multistems as the sum
e05.comp.index.d	Competition index D	CI assessed following Lorimer 1983, multistems assessing the sum of basal areas and then the dbh
e06.status		Dominant, co-dominant
e07.elevation	Elevation of the tree	Meters above sea level
e08.slope	Slope at the tree level	Slope in degrees
e09.aspect	Aspect at the tree level	0-360°
e10.soil.depth	Mean soil depth	Mean of 3 measures (measurement to a max. depth of 60 cm
e11.stone.content	Mean stone content	Mean of 3 measures (1: none, 2: little (5-20%), 3: low (20-40%), 4: medium (40-60%), 5: high (60-80%), 6: very high(80-95%), 7: full cover (>95%)
e12.rock.cover	Total rock cover	1: none, 2: little (5-20%), 3: low (20-40%), 4: medium (40-60%), 5: high (60-80%), 6: very high(80-95%), 7: full cover (>95%)

Table 2 Environmental variable names, explanations, and specifications modellled for all 4959 trees and 194 GenTree sites.

**Variable name**	**Variable explanation**	**Specification**
**GenTree Platform modelled environmental parameters**		
sample	Sample identification	unit: none; resolution [m]: none
country	Country code	unit: none; resolution [m]: none
countryspecies	Country and species code	unit: none; resolution [m]: none
species	Species code	unit: none; resolution [m]: none
population	Population identification	unit: none; resolution [m]: none
latwgs84	Latitude in WGS84	unit: none; resolution [m]: 25
lonwgs84	Longitude in WGS84	unit: none; resolution [m]: 25
latetrs89	Latitude in ETRS89	unit: none; resolution [m]: 25
lonetrs89	Longitude in ETRS89	unit: none; resolution [m]: 25
t01alt	Altitude	unit: m; resolution [m]: 25
t02slp	Slope	unit: degrees; resolution [m]: 25
t03asp	Eastness	unit: degrees; resolution [m]: 25
t04vcu	Profile curvature	unit: degrees/m; resolution [m]: 25
t05hcu	Horizontal curvature	unit: degrees/m; resolution [m]: 25
t06ddg	Downslope distance gradient	unit: degrees; resolution [m]: 25
t07mpi	Morphometric protection index	unit: none; resolution [m]: 25
t08tpi	Topographic position index	unit: none; resolution [m]: 25
t09vrm	Vector ruggedness measure	unit: none; resolution [m]: 25
t10twi	Topographic wetness index	unit: none; resolution [m]: 25
t11svf	Sky-view factor	unit: none; resolution [m]: 25
t12sdir	Potential direct solar radiation	unit: kJ/m^2; resolution [m]: 25

t13sdif	Potential diffuse solar radiation	unit: kJ/m^2; resolution [m]: 25
t14stot	Potential total solar radiation	unit: kJ/m^2; resolution [m]: 25
awc15	Available water capacity (0-30cm)	unit: %; resolution [m]: 250
awc140	Available water capacity (60-200cm)	unit: %; resolution [m]: 250
bio01	Yearly mean temperature	unit: °C; resolution [m]: 1000
bio02	Mean diurnal range	unit: none; resolution [m]: 1000
bio03	Isothermality	unit: none; resolution [m]: 1000
bio04	Temperature seasonality	unit: °C; resolution [m]: 1000
bio05	Max temperature of warmest month	unit: °C; resolution [m]: 1000
bio06	Min temperature of coldest month	unit: °C; resolution [m]: 1000
bio07	Temperature annual range	unit: °C; resolution [m]: 1000
bio08	Mean temperature of wettest quarter	unit: °C; resolution [m]: 1000
bio09	Mean temperature of driest quarter	unit: °C; resolution [m]: 1000
bio10	Mean temperature of warmest quarter	unit: °C; resolution [m]: 1000
bio11	Mean temperature of coldest quarter	unit: °C; resolution [m]: 1000
bio12	Yearly precipitation sum	unit: mm/year; resolution [m]: 1000
bio13	Precipitation of wettest month	unit: mm/month; resolution [m]: 1000
bio14	Precipitation of driest month	unit: mm/month; resolution [m]: 1000
bio15	Precipitation seasonality	unit: none; resolution [m]: 1000
bio16	Precipitation of wettest quarter	unit: mm/quarter; resolution [m]: 1000
bio17	Precipitation of driest quarter	unit: mm/quarter; resolution [m]: 1000
bio18	Precipitation of warmest quarter	unit: mm/quarter; resolution [m]: 1000
bio19	Precipitation of coldest quarter	unit: mm/quarter; resolution [m]: 1000
gdd	Growing degree days	unit: °C; resolution [m]: 1000
gsp	Accumulated precipitation	unit: kg/m^2; resolution [m]: 1000
shc	Hydrothermic coefficient	unit: (kg/m^2/10)/°C; resolution [m]: 1000
rh410	Relative humidity	unit: none; resolution [m]: 1000
fcf	Frost change frequency	unit: events; resolution [m]: 1000
nfd	Number of frost days	unit: days; resolution [m]: 1000
prec01	Precipitation sum in January	unit: mm/month; resolution [m]: 1000
prec02	Precipitation sum in February	unit: mm/month; resolution [m]: 1000
prec03	Precipitation sum in March	unit: mm/month; resolution [m]: 1000
prec04	Precipitation sum in April	unit: mm/month; resolution [m]: 1000
prec05	Precipitation sum in May	unit: mm/month; resolution [m]: 1000
prec06	Precipitation sum in June	unit: mm/month; resolution [m]: 1000
prec07	Precipitation sum in July	unit: mm/month; resolution [m]: 1000
prec08	Precipitation sum in August	unit: mm/month; resolution [m]: 1000
prec09	Precipitation sum in September	unit: mm/month; resolution [m]: 1000
prec10	Precipitation sum in October	unit: mm/month; resolution [m]: 1000
prec11	Precipitation sum in November	unit: mm/month; resolution [m]: 1000
prec12	Precipitation sum in December	unit: mm/month; resolution [m]: 1000
tmean01	Mean temperature in January	unit: °C; resolution [m]: 1000
tmean02	Mean temperature in February	unit: °C; resolution [m]: 1000
tmean03	Mean temperature in March	unit: °C; resolution [m]: 1000
tmean04	Mean temperature in April	unit: °C; resolution [m]: 1000
tmean05	Mean temperature in May	unit: °C; resolution [m]: 1000
tmean06	Mean temperature in June	unit: °C; resolution [m]: 1000
tmean07	Mean temperature in July	unit: °C; resolution [m]: 1000
tmean08	Mean temperature in August	unit: °C; resolution [m]: 1000
tmean09	Mean temperature in September	unit: °C; resolution [m]: 1000
tmean10	Mean temperature in October	unit: °C; resolution [m]: 1000
tmean11	Mean temperature in November	unit: °C; resolution [m]: 1000
tmean12	Mean temperature in December	unit: °C; resolution [m]: 1000
tmin01	Minimum temperature in January	unit: °C; resolution [m]: 1000
tmin02	Minimum temperature in February	unit: °C; resolution [m]: 1000
tmin03	Minimum temperature in March	unit: °C; resolution [m]: 1000
tmin04	Minimum temperature in April	unit: °C; resolution [m]: 1000
tmin05	Minimum temperature in May	unit: °C; resolution [m]: 1000
tmin06	Minimum temperature in June	unit: °C; resolution [m]: 1000
tmin07	Minimum temperature in July	unit: °C; resolution [m]: 1000
tmin08	Minimum temperature in August	unit: °C; resolution [m]: 1000
tmin09	Minimum temperature in September	unit: °C; resolution [m]: 1000
tmin10	Minimum temperature in October	unit: °C; resolution [m]: 1000
tmin11	Minimum temperature in November	unit: °C; resolution [m]: 1000
tmin12	Minimum temperature in December	unit: °C; resolution [m]: 1000
tmax01	Maximum temperature in January	unit: °C; resolution [m]: 1000
tmax02	Maximum temperature in February	unit: °C; resolution [m]: 1000
tmax03	Maximum temperature in March	unit: °C; resolution [m]: 1000
tmax04	Maximum temperature in April	unit: °C; resolution [m]: 1000
tmax05	Maximum temperature in May	unit: °C; resolution [m]: 1000
tmax06	Maximum temperature in June	unit: °C; resolution [m]: 1000

tmax07	Maximum temperature in July	unit: °C; resolution [m]: 1000
tmax08	Maximum temperature in August	unit: °C; resolution [m]: 1000
tmax09	Maximum temperature in September	unit: °C; resolution [m]: 1000
tmax10	Maximum temperature in October	unit: °C; resolution [m]: 1000
tmax11	Maximum temperature in November	unit: °C; resolution [m]: 1000
tmax12	Maximum temperature in December	unit: °C; resolution [m]: 1000

The local environmental contrasts varied among species and population pairs, most of which exhibited variability with respect to elevation, temperature, rainfall, and water availability. Other local contrasts were based on radiation, soil water capacity, and topographic wetness index (among others). One special case is *Populus nigra*, a heliophilous pioneer species found naturally in riverine areas. Given this specific habitat, local contrasts were largely bound to the distance of the individual trees from the riverbed and thus for example to ground water access or exposure to variation in the intensity and frequency of floods.

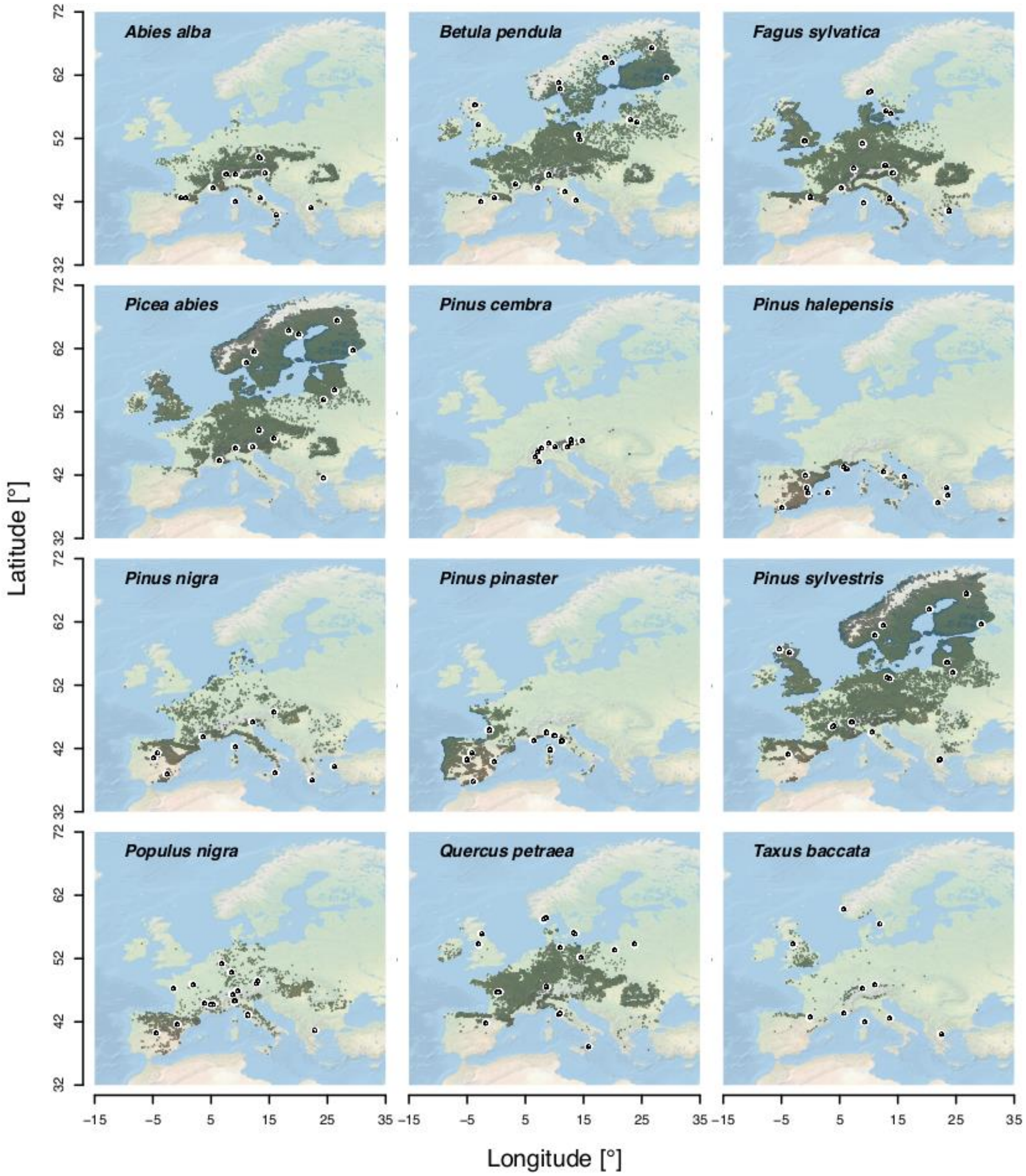

Fig. 1 Sampling sites and distributions of the twelve selected tree species for *in-situ* phenotype measurements. Distribution maps are based on a comprehensive high-resolution tree occurrence dataset from the European Union. [28]

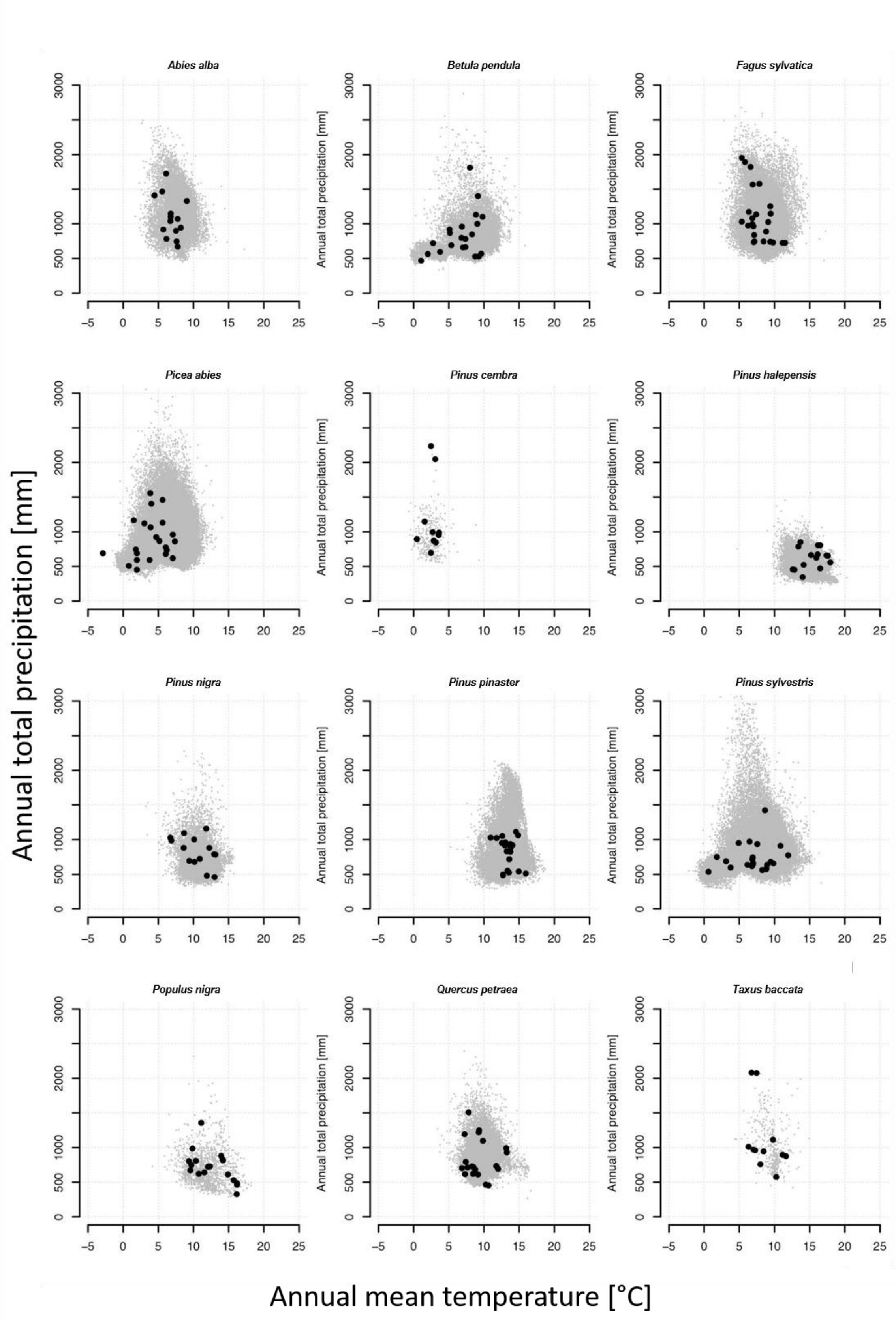

Fig. 2 Climate-space diagrams for the 12 selected species with annual mean temperature on the x-axis and annual total precipitation on the y-axis. Grey points represent species occurrences based on a comprehensive high-resolution tree occurrence dataset for Europe [28] and black dots indicate the GenTree sites.

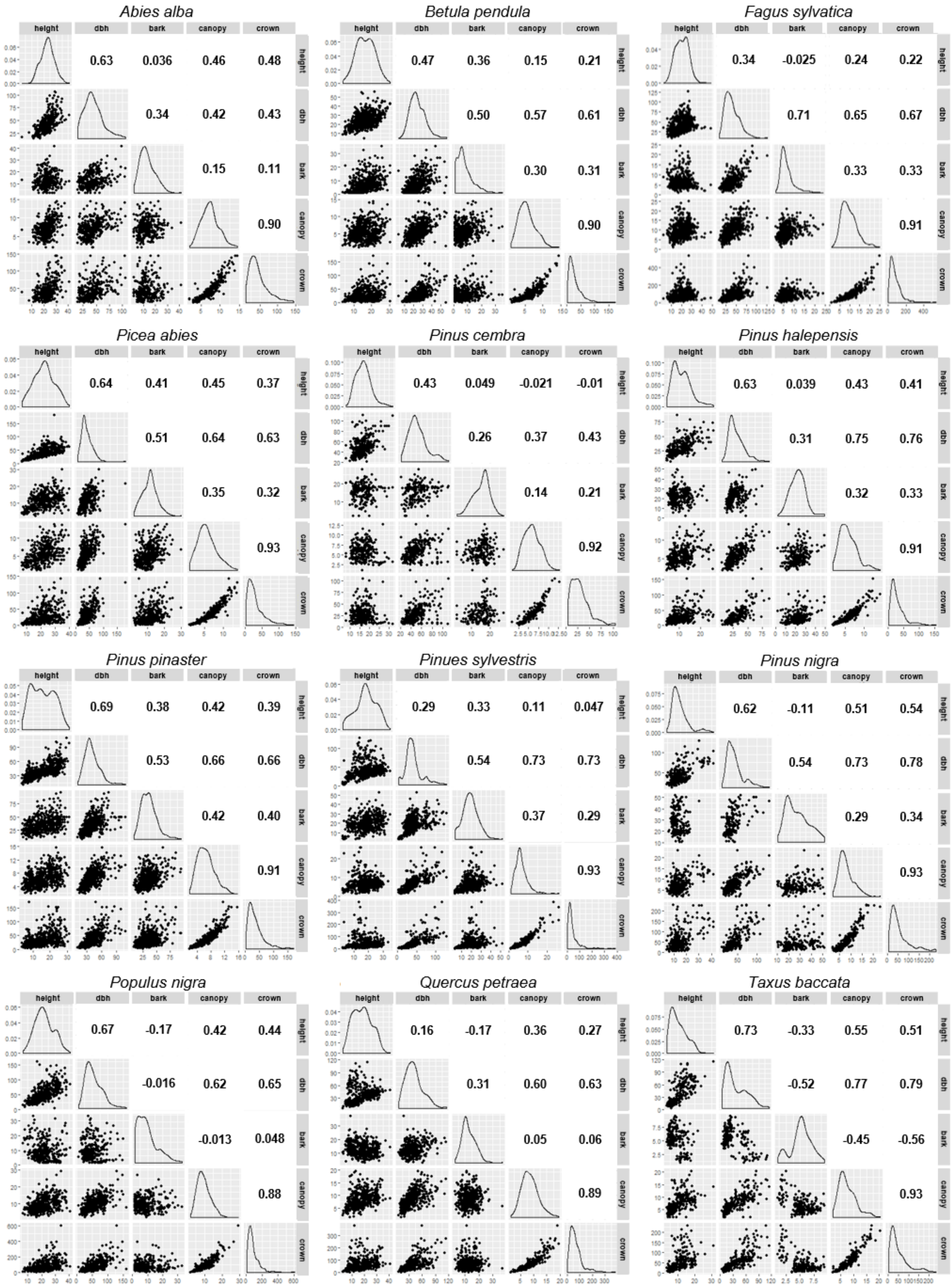

Fig. 3 Scatterplots, distributions and Pearson's correlation coefficients, *r*, of GenTree phenotypes in 12 European tree species.

Data validation and quality control

The database has been checked for consistency at different stages by various researchers between 2018-2020. Raw data were submitted by all partners to the GnpIS multispecies integrative information system (https://urgi.versailles.inra.fr/Tools/GnpIS) using preformatted Microsoft Excel templates. Data files were harmonized, merged and subsequently verified following several steps:

1. Missing data and dubious entries were checked manually by examining the original data files obtained from the partners and for identified cases compared to field books.
2. Descriptive statistics were calculated and plotted for all variables including minima, maxima, means, and variances. Outliers were checked against original data records, and corrected when necessary.
3. Covariables were plotted determining whether relationships were reasonable and following the most complete set of similar relationships (Fig. 3).

Acknowledgements¶

We would like to thank Juri Nievergelt, Anne Verstege, Marina Fonti, Kevin Kleeb, Frederick Reinig, Enrica Zalloni, Claudio de Sassi, Giacomo Poli for their field assistance. This work was performed using GnpIS, one of the facilities of the URGI platform (https://urgi.versailles.inra.fr/). We are also grateful to all the forest owners and national administrations for providing sampling permissions.

Author contributions¶

LO, RB, KH, BF, TM, FV, FAA, SC coordinated sampling design.
All authors contributed to sampling design
LO, RB, KH, BF, TM, FV, FAA, SC coordinated field sampling.¶
All authors contributed to the field sampling.
CM, MB compiled and assembled in situ measurements in the GnpIS database.¶
BD extracted climate and topographic data and derived environmental indices for all sampling sites.¶
RB, LO, BD, PAA, CM curated data, checked quality, and prepared the data sets with metadata files for sharing and potential reuse.¶
LO, KH, BD, SC, BF wrote the manuscript.¶
BF coordinated GenTree
All authors commented on an earlier version and approved the current version of the manuscript.

Competing interests¶

The authors declare no competing interests.¶

Funding

This publication is part of the GenTree project which was funded by the European Union's Horizon 2020 research and innovation program under grant agreement No. 676876 (GenTree). This work was also supported by the Swiss Secretariat for Education, Research and Innovation (SERI) under contract No. 6.0032.

References

1. Sitch, S. *et al.* Evaluation of the terrestrial carbon cycle, future plant geography and climate-carbon cycle feedbacks using five Dynamic Global Vegetation Models (DGVMs). *Global Change Biology* **14,** 2015–2039; 10.1111/j.1365-2486.2008.01626.x (2008).
2. Loarie, S. R. *et al.* The velocity of climate change. *Nature* **462,** 1052–1055; 10.1038/nature08649 (2009).
3. Pachauri, R. K. & Mayer, L. (eds.). *Climate change 2014. Synthesis report* (Intergovernmental Panel on Climate Change, Geneva, Switzerland, 2015).
4. Buras, A., Rammig, A. & Zang, C. Quantifying impacts of the drought 2018 on European ecosystems in comparison to 2003. *arxiv* (2018).
5. Savolainen, O., Pyhäjärvi, T. & Knürr, T. Gene Flow and Local Adaptation in Trees. *Annu. Rev. Ecol. Evol. Syst.* **38,** 595–619; 10.1146/annurev.ecolsys.38.091206.095646 (2007).
6. Kremer, A. *et al.* Long-distance gene flow and adaptation of forest trees to rapid climate change. *Ecology Letters* **15,** 378–392; 10.1111/j.1461-0248.2012.01746.x (2012).
7. Eveno, E. *et al.* Contrasting patterns of selection at Pinus pinaster Ait. Drought stress candidate genes as revealed by genetic differentiation analyses. *Molecular Biology and Evolution* **25,** 417–437; 10.1093/molbev/msm272 (2008).
8. Holliday, J. A., Ritland, K. & Aitken, S. N. Widespread, ecologically relevant genetic markers developed from association mapping of climate-related traits in Sitka spruce (Picea sitchensis). *New Phytologist* **188,** 501–514; 10.1111/j.1469-8137.2010.03380.x (2010).
9. Chen, J. *et al.* Clinal variation at phenology-related genes in spruce: parallel evolution in FTL2 and Gigantea? *Genetics* **197,** 1025–1038; 10.1534/genetics.114.163063 (2014).
10. González-Martínez, S. C., Ersoz, E., Brown, G. R., Wheeler, N. C. & Neale, D. B. DNA sequence variation and selection of tag single-nucleotide polymorphisms at candidate genes for drought-stress response in Pinus taeda L. *Genetics* **172,** 1915–1926; 10.1534/genetics.105.047126 (2006).
11. González-Martínez, S. C., Krutovsky, K. V. & Neale, D. B. Forest-tree population genomics and adaptive evolution. *New Phytologist* **170,** 227–238; 10.1111/j.1469-8137.2006.01686.x (2006).
12. Yeaman, S. *et al.* Convergent local adaptation to climate in distantly related conifers. *Science (New York, N.Y.)* **353,** 1431–1433; 10.1126/science.aaf7812 (2016).
13. Scotti, I., González-Martínez, S. C., Budde, K. B. & Lalagüe, H. Fifty years of genetic studies: what to make of the large amounts of variation found within populations? *Annals of Forest Science* **73,** 69–75; 10.1007/s13595-015-0471-z (2016).
14. Alexandre, H. *et al. In situ estimation of genetic variation of functional and ecological traits in Quercus petraea and Q.robur* (2018).
15. Dungey, H. S. *et al.* Phenotyping Whole Forests Will Help to Track Genetic Performance. *Trends in Plant Science* **23,** 854–864; 10.1016/j.tplants.2018.08.005 (2018).
16. Martínez-Sancho, E. *et al.* The GenTree Dendroecological Collection, tree-ring and wood density data from seven tree species across Europe. *Scientific Data* **7,** 1; 10.1038/s41597-019-0340-y (2020).
17. Steinbach, D. *et al.* GnpIS: an information system to integrate genetic and genomic data from plants and fungi. *Database : the journal of biological databases and curation* **2013,** bat058; 10.1093/database/bat058 (2013).

18. Lotterhos, K. E. & Whitlock, M. C. Evaluation of demographic history and neutral parameterization on the performance of FST outlier tests. *Molecular Ecology* **23,** 2178–2192; 10.1111/mec.12725 (2014).
19. Lotterhos, K. E. & Whitlock, M. C. The relative power of genome scans to detect local adaptation depends on sampling design and statistical method. *Molecular Ecology* **24,** 1031–1046; 10.1111/mec.13100 (2015).
20. Eckert, A. J. *et al.* Back to nature: ecological genomics of loblolly pine (Pinus taeda, Pinaceae). *Molecular Ecology* **19,** 3789–3805; 10.1111/j.1365-294X.2010.04698.x (2010).
21. Lind, B. M. *et al.* Water availability drives signatures of local adaptation in whitebark pine (Pinus albicaulis Engelm.) across fine spatial scales of the Lake Tahoe Basin, USA. *Molecular Ecology* **26,** 3168–3185; 10.1111/mec.14106 (2017).
22. Canham, C. D., LePage, P. T. & Coates, K. D. A neighborhood analysis of canopy tree competition: effects of shading versus crowding. *Can. J. For. Res.* **34,** 778–787; 10.1139/x03-232 (2004).
23. Lorimer, C. G. Tests of age-independent competition indices for individual trees in natural hardwood stands. *Forest Ecology and Management* **6,** 343–360 (1983).
24. Leempoel, K. *et al.* Very high-resolution digital elevation models: are multi-scale derived variables ecologically relevant? *Methods Ecol Evol* **6,** 1373–1383; 10.1111/2041-210X.12427 (2015).
25. Conrad, O. *et al.* System for Automated Geoscientific Analyses (SAGA) v. 2.1.4. *Geosci. Model Dev. Discuss.* **8,** 2271–2312; 10.5194/gmdd-8-2271-2015 (2015).
26. Hengl, T. *et al.* Mapping Soil Properties of Africa at 250 m Resolution: Random Forests Significantly Improve Current Predictions. *PLoS ONE* **10,** e0125814; 10.1371/journal.pone.0125814 (2015).
27. Karger, D. N. *et al.* Climatologies at high resolution for the earth's land surface areas. *Scientific Data* **4,** 170122; 10.1038/sdata.2017.122 (2017).
28. Mauri, A., Strona, G. & San-Miguel-Ayanz, J. EU-Forest, a high-resolution tree occurrence dataset for Europe. *Scientific Data* **4,** 160123; 10.1038/sdata.2016.123 (2017).
29. Barret Schloerke, Jason Crowley, Di Cook, Francois Briatte, Moritz Marbach, Edwin Thoen, Amos Elberg and Joseph Larmarange. *GGally: Extension to 'ggplot2'* (2020).

Personal Cover Click here to access/download;Personal Cover;Dear Editor.docx

Dear Editor,

On behalf of my co-authors, I am pleased to submit the data notes manuscript entitled *The GenTree Platform: growth traits and tree-level environmental data in twelve European forest tree species.* The data are accessible in figshare through this link: (https://figshare.com/s/4d57474fd63864a6dfd8). The data here presented include growth traits (height, dbh, bark thickness, crown size, trunk straightness, branch angles, forking indices) in 4,569 trees from twelve of the most ecologically and economically important tree species in Europe, sampled across their geographic range from a total of 194 sites. Furthermore, the data include tree level abiotic and biotic environmental parameters from in-situ assessment (plant cover, competition indices, elevation, slope, aspect, soil depth, stone content, rock cover), as well as downscaled descriptors based on climate and topography.

As we have used a coherent sampling design across species, our data offer unique opportunities to investigate tree growth on a continental scale that is difficult to achieve with existing databases such as the Try trait database, given that sampling approaches vary in such databases. Also, our data provide opportunities to be linked to remote sensing e.g. for ground truthing of biomass assessments. Furthermore, the data presented are the backbone for additional focused datasets as our data provide environmental context for those as well as crucial growth parameters:

- 'The GenTree Leaf Collection: inter- and intraspecific variation of leaf traits in seven forest tree species across Europe' (Benavides et al., submitted elsewhere),
- 'The GenTree Dendroecological Collection, tree-ring and wood density data from seven tree species across Europe", (Martinez-Sancho et al. 2020, SD 7 (1), 1-7), and
- "The GenTree Genomic Collection" which contains sequence data of ~800 candidate genes sequenced across seven of the 12 species and will be published elsewhere.

The data presented here and of the associated GenTree collections benefit from a unique, consistent sampling of natural populations in multiple species, designed within the GenTree project (funded by the European Union, H2020, www.gentree.eu). The sampling design aimed to capture both intra- and interspecific trait variability and full details are presented in the manuscript. The tree-level environmental information presented here can be directly used with the other datasets based on common site and individual tree identifiers.

Tree growth traits are included in many fields of study; thus, many scientists could be suggested as referees. I propose reviewers with well-known expertise on evolutionary and functional forest ecology:

- Sally N Aitken (The University of British Columbia, Canada, sally-aitken@ubc.ca),
- Sam Yeaman (University of Calgary, Canada, samuel.yeaman@ucalgary.ca),
- Andreas Rigling (Eidgenössische Forschungsanstalt WSL, Switzerland, andreas.rignling@wsl.ch),
- Victoria L. Sork (UCLA, USA, vlsork@ucla.edu),
- Katie Lotterhos (NUCS, k.lotterhos@northeastern.edu), and
- David Coomes (Cambridge Uni, dac18@cam.ac.uk).

I confirm that this manuscript has not been published elsewhere and it is not under consideration by any other journal, and that all the authors agree with the submission to GigaScience.

Thank you for considering our manuscript. I look forward to hearing from you.

Sincerely,

Lars Opgenoorth
