# Supplementary material for: The GenTree Platform: growth traits and tree-level environmental data in 12 European forest tree species
Source: Gigascience. 2021 Mar 18;10(3):giab010. doi: 10.1093/gigascience/giab010 (PMC7970660; doi:10.1093/gigascience/giab010)
Supplement: giab010_GIGA-D-20-00189_Revision_1 [file giab010_giga-d-20-00189_revision_1.pdf]

GigaScience

The GenTree Platform: growth traits and tree-level environmental data in twelve European forest tree species

--Manuscript Draft--

**Manuscript Number:**	GIGA-D-20-00189R1
**Full Title:**	The GenTree Platform: growth traits and tree-level environmental data in twelve European forest tree species
**Article Type:**	Data Note
**Funding Information:**	Horizon 2020 Framework Programme (676876) — Mr. Bruno Fady<br>Staatssekretariat für Bildung, Forschung und Innovation (6.0032) — Mr. Felix Gugerli
**Abstract:**	Background:<br>Progress in the field of evolutionary forest ecology has been hampered by the huge challenge of phenotyping trees across their ranges in their natural environments, and the limitation in high-resolution environmental information.<br>Findings:<br>The GenTree Platform contains phenotypic and environmental data from 4,959 trees from twelve ecologically and economically important European forest tree species: Abies alba Mill. (silver fir), Betula pendula Roth. (silver birch), Fagus sylvatica L. (European beech), Picea abies (L.) H. Karst (Norway spruce), Pinus cembra L. (Swiss stone pine), Pinus halepensis Mill. (Aleppo pine), Pinus nigra Arnold (European black pine), Pinus pinaster Aiton (maritime pine), Pinus sylvestris L. (Scots pine), Populus nigra L. (European black poplar), Taxus baccata L. (English yew), and Quercus petraea (Matt.) Liebl. (sessile oak). Phenotypic (height, diameter at breast height, crown-size, bark-thickness, biomass, straightness, forking, branch angle, fructification), regeneration, environmental in-situ measurements (soil depth, vegetation cover, competition indices), and environmental modeling data extracted by using bilinear interpolation accounting for surrounding conditions of each tree (precipitation, temperature, insolation, drought indices) were obtained from trees in 194 sites covering the species' geographic ranges and reflecting local environmental gradients.<br>Conclusion:<br>The GenTree Platform is a new resource for investigating ecological and evolutionary processes in forest trees. The coherent phenotyping and environmental characterization across 12 species in their European ranges allows for a wide range of analyses from forest ecologists, conservationists and macro-ecologists. In addition, the data here presented can be linked to the GenTree Dendroecological collection, the GenTree Leaf Trait collection, and the GenTree Genomic collection presented elsewhere, that together build the largest evolutionary forest ecology data collection available.
**Corresponding Author:**	Lars Opgenoorth<br>Philipps-Universität Marburg<br>Marburg, Hessen GERMANY
**Corresponding Author Secondary Information:**	
**Corresponding Author's Institution:**	Philipps-Universität Marburg
**Corresponding Author's Secondary Institution:**	
**First Author:**	Lars Opgenoorth
**First Author Secondary Information:**	
**Order of Authors:**	Lars Opgenoorth<br>Benjamin Dauphin<br>Raquel Benavides

Powered by Editorial Manager® and ProduXion Manager® from Aries Systems Corporation

	Katrin Heer
	Paraskevi Alizoti
	Elisabet Martínez-Sancho
	Ricardo Alía
	Olivier Ambrosio
	Albet Audrey
	Camilla Avanzi
	Evangelia Avramidou
	Francesca Bagnoli
	Evangelos Barbas
	Cristina C. Bastias
	Catherine Bastien
	Eduardo Ballesteros
	Giorgia Beffa
	Frédéric Bernier
	Henri Bignalet
	Guillaume Bodineau
	Damien Bouic
	Sabine Brodbeck
	William Brunetto
	Jurata Buchovska
	Melanie Buy
	Ana M. Cabanillas-Saldaña
	Bárbara Carvalho
	Nicolas Cheval
	José M. Climent
	Marianne Correard
	Eva Cremer
	Darius Danusevičius
	Fernando Del Caño
	Jean-Luc Denou
	Nicolas di Gerardi
	Bernard Dokhelar
	Alexis Ducousso
	Eskild Nilsen
	Anna-Maria Farsakoglou
	Patrick Fonti
	Ioannis Ganopoulos
	José M. García del Barrio
	Olivier Gilg
	Santiago C. González-Martínez

Powered by Editorial Manager® and ProduXion Manager® from Aries Systems Corporation

	René Graf
	Alan Gray
	Delphine Grivet
	Felix Gugerli
	Christoph Hartleitner
	Enja Hollenbach
	Agathe Hurel
	Bernard Issehut
	Florence Jean
	Veronique Jorge
	Arnaud Jouineau
	Jan-Philipp Kappner
	Katri Kärkkäinen
	Robert Kesälahti
	Florian Knutzen
	Sonja Kujala
	Timo Kumpula
	Mariaceleste Labriola
	Celine Lalanne
	Johannes Lambertz
	Martin Lascoux
	Vincent Lejeune
	Gregoire Le-Provost
	Joseph Levillain
	Mirko Liesebach
	David López-Quiroga
	Benjamin Meier
	Ermioni Malliarou
	Jérémy Marchon
	Nicolas Mariotte
	Antonio Mas
	Silvia Matesanz
	Helge Meischner
	Célia Michotey
	Pascal Milesi
	Sandro Morganti
	Daniel Nievergelt
	Eduardo Notivol
	Geir Ostreng
	Birte Pakull
	Annika Perry

Powered by Editorial Manager® and ProduXion Manager® from Aries Systems Corporation

	Andrea Piotti
	Christophe Plomion
	Nicolas Poinot
	Mehdi Pringarbe
	Luc Puzos
	Tanja Pyhäjärvi
	Annie Raffin
	José A. Ramírez-Valiente
	Christian Rellstab
	Dourthe Remi
	Sebastian Richter
	Juan J. Robledo-Arnuncio
	Sergio San Segundo
	Outi Savolainen
	Volker Schneck
	Ivan Scotti
	Vladimir Semerikov
	Lenka Slámová
	Jørn Henrik Sønstebø
	Ilaria Spanu
	Jean Thevenet
	Mari Mette Tollefsrud
	Norbert Turion
	Giovanni Giuseppe Vendramin
	Marc Villar
	Georg Von Arx
	Johan Westin
	Bruno Fady
	Tor Myking
	Fernando Valladares
	Filippos A. Aravanopoulos
	Stephen Cavers
**Order of Authors Secondary Information:**	
**Response to Reviewers:**	General comments from the Editor<br>The reviewers' reports are below. Please pay particular attention to reviewer 1's points regarding sampling methodology and data structure (e.g. will it be possible to include time series / repeated measurements?).<br>Please include a point-by-point within the 'Response to Reviewers' box in the submission system. Please ensure you describe additional experiments that were carried out and include a detailed rebuttal of any criticisms or requested revisions that you disagreed with. Please also ensure that your revised manuscript conforms to the journal style, which can be found in the Instructions for Authors on the journal homepage. If the data and code has been modified in the revision process please be

sure to update the public versions of this too.

Reviewer reports:
Reviewer #1
This Data Note introduces the 'GenTree Platform' - consisting of phenotypic (coarse tree-level traits) and environmental data at individual tree level from ~5000 trees of 12 important European species. At each tree, a set of traits are scored and data taken on soil depth, vegetation and other site data at 194 sites (covering ranges of species and environmental gradients). Modelled climate and soil data (i.e., from layers) have been extracted from external datasets and presented as well. Populations (25 trees) were sampled at up to 20 sites per species.
The dataset forms a new fragment of a larger database 'GenTree' with leaf trait, genomic and dendroecological nodes. It is not clear why each of these modules should be treated individually when they appear to deal with the same trees but perhaps that is for historical and practical reasons.
The dataset appears to be well formulated, significant in size and collection/collaboration effort, and is a useful and novel addition to functional ecology data available, in that it is presented in an easily accessible way and provides data not easily otherwise available. The gentree dataset as a whole appears to be significant in its standardised methods and depth of data at tree level on many facets of genetics, traits and environment.
MAJOR COMMENTS:
1.I don't think there is any reference to repeat measurements other than mention of tagging for 'potential subsequent additional ... sampling'? Does this dataset represent a single measurement - presumably it does? Is it intended that these tagged trees and sites be re-measured? If so, with what protocol and frequency (i.e. what needs to be repeated and what doesn't) and how will that data be integrated or presented with these?
The reviewer is right, that there is no resampling scheme in place. Instead, the permanent marking of trees is meant to offer the chance either to individual studies aimed just at a subset of sites/trees or to a later comprehensive project to resample trees and thus make use of time-series data. For example, there is a new EU project called FORGENIUS (http://www.euforgen.org/about-us/news/news-detail/forgenius-a-new-project-to-revolutionise-our-understanding-of-forest-diversity/), that will start January 1st and will make use of a subset of the sites/trees by revisiting them and obtaining additional phenotypic measurements. That said, we have now stated this point explicitly in the text starting in line 338:
“Every tree was permanently labeled so that future studies can resample subsets or the entire GenTree collection for gaining time-series data of individual traits or to add new phenotypes to the analyses. Be aware, that permission of the respective landowners must be obtained prior to sampling.”
2.The data are arranged by country/species/population/individual as far as I can tell - how are measurements in a time series (whether it yet exists or not) identified - should there be a visit number, date or ID field as well? I don't get the feeling that future data addition and re-sampling has been incorporated specifically in the protocols or data formatting. This needs to be addressed in the manuscript and the date/year or sampling should appear in the data in any case (a visit or measurement series number field may also be useful as data are added temporally).
We agree with the reviewer and have thus added columns with the respective visiting dates in the data frame.
3.The introductory parts suggest that the dataset addresses limitations in high-resolution environmental information associated with trees. Certainly, the site-based soil and topography measurements address this, but the additional soil grids and CHELSA climate data do not increase environmental information, only extract and present it alongside the site data in a convenient analysis-ready format. Please revise the text to be clear that this aspect of the dataset is a compilation/merge of data, not the creation of new high-resolution environmental data.
It is correct that only our on-site measurements are really highly resolved environmental data in the given context and that some of the modeled data resolves mainly on site-scale. However, some of the modelled data is downscaled to tree-level which is an added value, a step that does increase the environmental information on top of the noted convenience. However, we agree that we should be more specific here as to not raise wrong expectations in the introduction. The text in line 264 ff now reads:

Powered by Editorial Manager® and ProduXion Manager® from Aries Systems Corporation

	“However, progress in this field has been hampered by limited genomic resources, the lack of small-scale, individual tree-level-resolution environmental information 13…. In addition to onsite environmental measurements, we provide modelled and downscaled data for our sites and for individual tree level respectively.”<br>4.Line numbers would have been convenient for reference.<br>We are sorry! We have included them now.<br>MINOR COMMENTS:<br>5.Abstract: Please correct inconsistent capitalisation of common names.<br>I assume you are referring to the spelling of Silver fir – which should be silver fir. Thank you for pointing it out. Just in case you are referring to the fact that some of the names are in capitals and others not – this is intentional – as the correct spelling varies – e.g. when a country name is involved as in Swiss stone pine – then Swiss needs to be capitalized.<br>6.CONTEXT - I think the context should give brief 'context' of the data type presented in relation to other datasets. There are clear differences compared to species and plot level measurements of tree traits and cover etc. but it could be useful for a potential user to get a feel for this here given there are now a number of databases. What other available datasets include comparable individual tree data? There will be a number for dbh at least but the value of the gentree dataset seems to lie in the depth of other information collected and compiled for each tree, whereas other forest datasets focus more on good distribution/population data for stands in height, dbh, mortality etc. of many trees - so please make this point of difference clear here (individual versus stand level data) if that is the correct focus. What is different about the dataset and how does it fit with others?<br>We appreciate the need to give some background on other available datasets/databases. We have added this paragraph starting in line 283:<br>“We investigated the extent to which other datasets comparable to the data presented here exist by screening our twelve species in the TRY Plant Trait Data Base, the ITDRB, and the biomass and allometry data base for woody plants (BAAD). While this is a systematic approach, it leaves out a large number of tree species and therefore we cannot claim to have a comprehensive overview of the existing data. However, all three databases are large collections that include at least some of the tree measurements we present. Even though these are tremendous resources, the major difference is that based on their nature as collecting points of numerous independent datasets, there is no coherent sampling scheme in these collections as such, meaning that the number of trees per site, the way of tree selection, measured phenotypes, and provided environmental information vary greatly and therefore do not allow for coherent comparative analyses such as those of the GenTree Platform. For example, BAAD reports DBH data for only four of the species presented here, namely Betula pendula with three populations, Fagus sylvatica with two populations, Picea abies with four populations, and Pinus sylvestris with ten populations. In the larger TRY database, all of our species are represented, but the variability of sampling schemes is much more heterogenous in relation to traits, number of populations per species, and metadata. For example, DBH measurements are being reported 232 times from a total of 12 Betula pendula populations. Of these the vast majority of the 170 measurements are from one population while from many other populations only one or up to five measurements are reported. Also, the measurements stem from five different original studies and thus having very different levels of additional information. We conclude that the core value of our reported data lies in the coherent sampling design and yet large number of sampled populations and individuals per species.”<br>7.First sentence reads as though climate and land use changes will only have a future and not a present-day impact.<br>Changed. It now states starting in line 235:<br>“The impacts of climate change and land-use change on forests are already severe, as observed, for example, following the extreme summer drought of 2018 that triggered a massive increase in mortality in Central European forests1. Furthermore, changes are expected to be acute in the future,”<br>8.re:'In the light of these changes, species and forest ecosystem resilience will depend on the extent and structure of genetic variation and adaptive potential.' What about physiological resilience/acclimation at individual level as well as dispersal?<br>Changed. The sentence now states starting in line 244:<br>“In the light of these changes, species and forest ecosystem resilience will depend on the extent and structure of phenotypic plasticity, genetic variation and adaptive potential, as well as dispersal ability.”

9.re:'extensive gene flow' Could extensive gene flow not work against local adaptation?
It does for sure. The term "extensive gene flow" was misplaced in the flow of the sentence. It now reads starting in line 248:
"In Europe, where most tree populations have established following post-glacial recolonization, such patterns of local adaptation must have developed rapidly and despite long generation time and extensive gene flow , a process enabled by high levels of within-population plasticity, genetic and epigenetic variation, and large population sizes ."
Thank you for pointing it out!
10.METHODS - 'Growth' traits are only mentioned in the title - can you define or refer to this phrase here? It is only called 'tree phenotypes' here. Growth could equally be structural or some other word - from the title I was expecting traits relating to growth rate or mode.
We exchanged the term tree phenotype with growth traits in line 389, as essentially all our tree level phenotypes can be regarded as growth traits.
11.Vegetation cover is estimated visually in coarse % categories. Is a proportion of that estimate is made up by the subject tree, or does that cover estimate exclude the tree itself? Does it include canopy/shrub/ground layers?
It only included ground vegetation – not the tree cover itself. We have now made this clear by adding "without tree cover" in the following sentence in line 377:
"Surrounding each target tree, slope, vegetation cover (without tree cover), and stone content were assessed in a 10 m x 10 m plot."
12.re:'show no signs of significant damage due to pests and diseases or generally low vigor' what is the justification for this? Can't stress/pest damage be a part of the trees' natural state in a given environment? e.g., how would this affect future repeat sampling of these permanently marked trees if, as you say, climate and land-use changes are expected to significantly impact tree regeneration, growth and health? Or how would one go about sampling additional sites with your method if there were no healthy trees remaining?
You are of course right, that stress/pest damage are part of the trees natural states and could even be extremely interesting in association studies. However, given that each of such states would be additional "treatments" – our number of repeats on the single tree level would just not be sufficient to adequately analyze this. For this reason, we decided to choose only "normal" "healthy" trees.
13.Regeneration: why are seedlings not counted in the plot but scored categorically? Is it to save time?
Yes. Unfortunately – given that the field campaigns were very time and resource consuming we had to make some tough choices. This was one of them.
14.Climate: while CHELSA is a suitable dataset it isn't especially high resolution by today's standards - it is highly likely, for example, that gridded values extracted at the location of individual trees within a population/site will come from the same grid cell on a climate layer. Obviously, truly individual data would be in the realm of microclimatic measurement, which is not in the scope of this dataset (perhaps a consideration for future repeat sampling?) but is expected based on the abstract and introduction.
Needs some re-wording to be clear that this is an extraction/compilation exercise rather than data generation per se, i.e., you are making use of high-resolution products that are now available, and packing that data with your own.
We agree with the comments by the reviewer that CHELSA by itself is not capturing microclimatic conditions and indeed, as indicated in another reply, there is already at least one follow-up project that will utilize some of the sites and will install beside other climate sensors. That said, our extraction method using bilinear interpolation accounts for the surrounding conditions of each tree. We have complemented the original text in the abstract in line 216:
"…and environmental modeling data extracted by using bilinear interpolation accounting for surrounding conditions of each tree (precipitation, temperature, insolation, drought indices) were obtained from trees in 194 sites covering the species' geographic ranges and reflecting local environmental gradients."
Also we included this information in the Material and Methods section starting in line 470:
"We extracted all modeled environmental values for each individually geo-referenced tree using the extract function of the R package RASTER (Hijmans and van Etten 2016). The surrounding conditions (i.e. adjacent pixels) of each individual tree were incorporated by the bilinear interpolation method when extracting the data."

15.Figure 1. Perhaps self-evident, but there is no key - could you at least refer to sites (points) and distributions (dark green) or something in the caption? A useful and clear figure.
Thank you for pointing this out. We have rephrased the figure capture starting in line 501:
“Sampling sites (black dots) and distributions of the twelve selected tree species (dark green shading) for in-situ phenotype measurements. Distribution maps are based on a comprehensive high-resolution tree occurrence dataset from the European Union.”
16.Figures 2&3 are very useful information for re-use, thank you.
Thank you!
17.Data: There isn't an obvious reason why these data can't be presented in a single spreadsheet/file instead of three (metadata, site data and extracted environmental data) given they are in xls format (i.e., tabs for metadata) and the data tables both have fields per individual tree. Wouldn't that make it easier to access/store and use? But perhaps data modules are treated separately because there are many more available from other gentree nodes?
Indeed, our rationale is the inter-usability with other GenTree data and products, which is why we would like to retain the structure as is.
18.Unless I have missed it, appears not to have a field referring to survey number/ID/date etc, which means the structure will have to be changed as soon as repeat measures are included. Date/year should be a basic field in any case so that data can be matched to climate seasons/trends over time. The time of sampling appears to be excluded.
See comment above, we have now included this information.
19.Do you mention the open access CC0 licence anywhere in the paper (it appears in the linked figshare dataset)? Users will want to know if it is freely available as some such datasets require permission from the data owners.
Thank you for this note. The sentence now reads in line 482:
“These data files are available at figshare data repository open access under a CC0 license. (https://figshare.com/s/4d57474fd63864a6dfd8).”
Reviewer #2:
1) The manuscript present a relevant dataset for forest scientists. The dataset is broad and valuable. The manuscript is clear and it is well structured and written. Data are stored in figshare where the authors stated that 4 different files are included. However only three files are in the figshare site (https://figshare.com/s/4d57474fd63864a6dfd8) this point should clarify in the manuscript. Additionally , although it is indicated that 4 csv files are available in the repository (https://figshare.com/s/4d57474fd63864a6dfd8) only 3 files are availables and only one it is in csv (the otaher two are xlsx files) I suggest to complete the 4 files and use csv format for all of them.
The reviewer is right in relation to figshare. This was a confusion on our side, since for GigaScience we had to provide all data in csv files to their ftp server, we switched numbers. On the ftp server the env data is in 4 csv files. The way we present the data on figshare is only three files and it is the correct number of files in fact. We thus would like to keep them as they are there with the inclusion of sampling date though.

MINOR COMMENTS
In Methods (competition index at tree level)
1) I would suggest to reorder the author recognition by writing Lorimet et al and Canham et al (and not the reverse as is in the manuscript) because Lorimer et al paper is previous to the Lorimer et al one.
We agree and have changed the order in lines 360 following.
2) when authors note that 'each stem larger than 15 cm..' must be noted that this value is for dbh
Done. We have included the term DBH in line 375.
In Methods (regeneration
1) When talking about 'paternity', should maternity be written instead?
We agree and have changed it accordingly in line 387.
In Methods (tree phenotypes)
1) at the end of DBH (cm) indicate that average is used only when authors dealed with multistem trees.
There are two mentions of average in the DBH section. The first refers to the method when using a caliper. There two perpendicular measurements were made and then the average of the two was taken. We have now included this specification there in line 392:

	“DBH was measured at a stem height of 1.3 m using either a caliper by measuring two perpendicular diameters and subsequently taking the average of these two measurements or by measuring the circumference of the tree using a tape and computing the diameter from that value.”<br>In the second sentence, the average was recorded “if a tree had more than one trunk”. We think that this usage of average should be clear.<br>2) In the Heigh section, a clarification about slope correction method used when heigh measurements is needed<br>We have added the following text in line 400:<br>“To forego errors introduced by measuring height on sloping ground, height measurements on slopes were conducted from the same elevation as the tree’s base by approaching the tree sideways. Where this was not possible, a slope correction factor was used.”<br><br>3) References - Please check this section some of the referencies are not complete (ie, # 4, 16, 29,...)<br>We have checked the references and corrected them where necessary. That said, we believe that the specifically mentioned reference #4 was cited correctly according the journals requirements.
**Additional Information:**	
**Question**	**Response**
Are you submitting this manuscript to a special series or article collection?	No
**Experimental design and statistics**<br><br>Full details of the experimental design and statistical methods used should be given in the Methods section, as detailed in our Minimum Standards Reporting Checklist. Information essential to interpreting the data presented should be made available in the figure legends.<br><br>Have you included all the information requested in your manuscript?	Yes
**Resources**<br><br>A description of all resources used, including antibodies, cell lines, animals and software tools, with enough information to allow them to be uniquely identified, should be included in the Methods section. Authors are strongly encouraged to cite Research Resource Identifiers (RRIDs) for antibodies, model organisms and tools, where possible.<br><br>Have you included the information	Yes

requested as detailed in our Minimum Standards Reporting Checklist?	
**Availability of data and materials**<br><br>All datasets and code on which the conclusions of the paper rely must be either included in your submission or deposited in publicly available repositories (where available and ethically appropriate), referencing such data using a unique identifier in the references and in the “Availability of Data and Materials” section of your manuscript.<br><br>Have you have met the above requirement as detailed in our Minimum Standards Reporting Checklist?	Yes

Powered by Editorial Manager® and ProduXion Manager® from Aries Systems Corporation

Manuscript

Click here to access/download;Manuscript;Opgenoorth et al 2020_resubmission.docx

The GenTree Platform: growth traits and tree-level environmental data in twelve European forest tree species

Opgenoorth, Lars[1,2], Dauphin, Benjamin[2], Benavides, Raquel[3], Heer, Katrin[1], Alizoti, Paraskevi[4], Martínez-Sancho, Elisabet[2], Alía, Ricardo[5], Ambrosio, Olivier[6], Audrey, Albet[7], Auñón, Francisco[5], Avanzi, Camilla[8], Avramidou, Evangelia[4], Bagnoli, Francesca[8], Barbas, Evangelos[4], Bastias, Cristina C.[9], Bastien, Catherine[10], Ballesteros, Eduardo[5], Beffa, Giorgia[2], Bernier, Frédéric[7], Bignalet, Henri[7], Bodineau, Guillaume[11], Bouic, Damien[7], Brodbeck, Sabine[2], Brunetto, William[6], Buchovska, Jurata[12], Buy, Melanie[6], Cabanillas-Saldaña, Ana M.[13], Carvalho, Bárbara[3], Cheval, Nicolas[7], Climent, José M.[5], Correard, Marianne[6], Cremer, Eva[14], Danusevičius, Darius[12], Del Caño, Fernando[5], Denou, Jean-Luc[7], di Gerardi, Nicolas[2], Dokhelar, Bernard[7], Ducousso, Alexis[15], Eskild Nilsen, Anne[16], Farsakoglou, Anna-Maria[4], Fonti, Patrick[2], Ganopoulos, Ioannis[17], García del Barrio, José M.[5], Gilg, Olivier[6], González-Martínez, Santiago C.[7], Graf, René[2], Gray, Alan[18], Grivet, Delphine[5], Gugerli, Felix[2], Hartleitner, Christoph[19], Hollenbach, Enja[1], Hurel, Agathe[7], Issehut, Bernard[7], Jean, Florence[6], Jorge, Veronique[20], Jouineau, Arnaud[6], Kappner, Jan-Philipp[1], Kärkkäinen, Katri[21], Kesälahti, Robert[22], Knutzen, Florian[14], Kujala, Sonja T[21], Kumpula, Timo A[22], Labriola, Mariaceleste[8], Lalanne, Celine[15], Lambertz, Johannes[1], Lascoux, Martin[23], Lejeune, Vincent[11], Le-Provost, Gregoire[15], Levillain, Joseph[24], Liesebach, Mirko[25], López-Quiroga, David[3], Meier, Benjamin[2], Malliarou, Ermioni[4], Marchon, Jérémy[2], Mariotte, Nicolas[6], Mas, Antonio[3], Matesanz, Silvia[26], Meischner, Helge[1], Michotey, Célia[27], Milesi, Pascal[28], Morganti, Sandro[2], Nievergelt, Daniel[2], Notivol, Eduardo[29], Ostreng, Geir[16], Pakull, Birte[25], Perry, Annika[18], Piotti, Andrea[8], Plomion, Christophe[15], Poinot, Nicolas[7], Pringarbe, Mehdi[6], Puzos, Luc[7], Pyhäjärvi, Tanja[22], Raffin, Annie[7], Ramírez-Valiente, José A.[5], Rellstab, Christian[2], Remi, Dourthe[7], Richter, Sebastian[1], Robledo-Arnuncio, Juan J.[5], San Segundo, Sergio[5], Savolainen,Outi[22], Schneck,Volker[30], Scotti, Ivan[6], Semerikov, Vladimir[31], Slámová, Lenka[2], Sønstebø, Jørn Henrik[16], Spanu, Ilaria[8], Thevenet, Jean[6], Tollefsrud, Mari Mette[16], Turion, Norbert[6], Vendramin, Giovanni Giuseppe[8], Villar, Marc[20], von Arx, Georg[2], Westin, Johan[32], Fady,Bruno[6], Myking, Tor[16], Valladares, Fernando[3], Aravanopoulos, Filippos A.[4], Cavers, Stephen[18]

[1]Philipps University Marburg, Faculty of Biology, Karl-von-Frisch Strasse 8, 35043 Marburg, Germany

[2]Swiss Federal Research Institute WSL, Zürcherstrasse 111, 8903, Birmensdorf, Switzerland

[3]LINCGlobal, Department of Biogeography and Global Change, Museo Nacional de Ciencias Naturales, CSIC, Serrano 115 dpdo, 28006, Madrid, Spain

[4]Aristotle University of Thessaloniki, School of Forestry and Natural Environment, Laboratory of Forest Genetics and Tree Improvement, 54124 Thessaloniki, Greece

[5]Instituto Nacional de Investigación y Tecnología Agraria y Alimentaria - Centro de Investigación Forestal (INIA-CIFOR), Ctra. de la Coruña km 7.5, 28040, Madrid, Spain

[6]Institut National de Recherche en Agriculture, Alimentation et Environment (INRAE), Domaine Saint Paul, Site Agroparc, 84914, Avignon, France

[7]Institut National de Recherche en Agriculture, Alimentation et Environment (INRAE), UEFP, 33610 Cestas, France

[8]Institute of Biosciences and BioResources, National Research Council (CNR), via Madonna del Piano 10, 50019, Sesto, Fiorentino, Italy

[9]Centre d'Ecologie Fonctionnelle et Evolutive (CEFE), CNRS, UMR 5175, Montpellier, France

[10]Institut National de Recherche en Agriculture, Alimentation et Environment (INRAE), Dept ECOFA, F-45075 Orléans, France

[11]Institut National de Recherche en Agriculture, Alimentation et Environment (INRAE), GBFOR, 45075 Orléans, France

[12]Vytautas Magnus University, Studentu Street 11, 53361, Akademija, Lithuania.

[13]Departamento de Agricultura, Ganadería y Medio Ambiente, Gobierno de Aragón, P. Mª Agustín 36, 50071 Zaragoza, Spain

[14]Bavarian Institute for Forest Genetics , Forstamtsplatz 1, 83317 Teisendorf, Germany

[15]INRAE, Univ. Bordeaux, BIOGECO, F-33770 Cestas, France
[16]Division of Forestry and Forest Resources, Norwegian Institute of Bioeconomy Research (NIBIO), P.O. Box 115, 1431, Ås, Norway
[17]Institute of Plant Breeding and Genetic Resources, Hellenic Agricultural Organization DEMETER (ex NAGREF), Thermi, 57001, Greece
[18]UK Centre for Ecology and Hydrology, Bush Estate Penicuik, EH26 0QB, United Kingdom
[19]LIECO GmbH & Co KG
[20]Institut National de Recherche en Agriculture, Alimentation et Environment (INRAE), ONF, BIOFORA, 45075 Orléans, France
[21]Natural Resources Institute Finland, Paavo Havaksentie 3, 90014, University of Oulu, Finland
[22]University of Oulu, Pentti Kaiteran katu 1, 90014, University of Oulu, Finland
[23]Department of Ecology & Genetics, EBC, Uppsala University, Norbyvägen 18D, 75236, Uppsala, Sweden
[24]Université de Lorraine, AgroParisTech, INRAE, SILVA, 54000 Nancy, France
[25]Thünen Institute of Forest Genetics, Sieker Landstr. 2, Grosshansdorf, 22927, Germany
[26]Área de Biodiversidad y Conservación, Universidad Rey Juan Carlos, Calle Tulipán s/n, 28933, Móstoles, Spain
[27]Institut National de Recherche en Agriculture, Alimentation et Environment (INRAE), URGI, Versailles, France
[28]Department of Ecology & Genetics, EBC, Science for Life Laboratory, Uppsala University, Uppsala, Sweden
[29]Centro de Investigación y Tecnología Agroalimentaria de Aragón - Unidad de Recursos Forestales (CITA),Avda. Montañana 930, 50059, Zaragoza, Spain
[30]Thünen Institute of Forest Genetics, Eberswalder Chaussee 3a, Waldsieversdorf, 15377, Germany
[31]Institute of Plant and Animal Ecology, Ural branch of RAS, 8 Marta St. 202, 620144, Ekaterinburg, Russia
[32]Skogforsk, Tomterna 1, 918 21 Sävar, Sweden

opgenoorth@uni-marburg.de; lars.opgenoorth@wsl.ch
benjamin.dauphin@wsl.ch
rbenavidescalvo@gmail.com
katrin.heer@uni-marburg.de
alizotp@for.auth.gr
elisabet.martinez@wsl.ch
alia@inia.es
olivier.ambrosio@inrae.fr
audrey.albet.1@inrae.fr
aunon.francisco@inia.es
camilla.avanzi@ibbr.cnr.it
aevaggelia@yahoo.com
francesca.bagnoli@ibbr.cnr.it
vbarbas@for.auth.gr
crbasc@gmail.com
catherine.bastien@inrae.fr
eduardo.ballesteros@inia.es
giorgia.beffa@gmail.com
frederic.bernier@inrae.fr
henri.bignalet@inrae.fr
guillaume.bodineau@inrae.fr
damien.bouic@inrae.fr
sabine.brodbeck@wsl.ch
william.brunetto@inrae.fr

103	jurata.buchovska@vdu.lt
104	Melanie.Buy@inrae.fr
105	acabanillas@aragon.es
106	babic.barbosa@gmail.com
107	nicolas.cheval@inrae.fr
108	climent@inia.es
109	marianne.correard@inrae.fr
110	Eva.Cremer@awg.bayern.de
111	darius.danusevicius@vdu.lt
112	umgf@inia.es
113	jean-luc.denou@inrae.fr
114	nicolas.degirardi@gmail.com
115	bernard.dokhelar@inrae.fr
116	alexis.ducousso@inrae.fr
117	anne.eskild.nilsen@nibio.no
118	amfarsakoglou@gmail.com
119	patrick.fonti@wsl.ch
120	giannis.ganopoulos@gmail.com
121	jmgarcia@inia.es
122	olivier.gilg@inrae.fr
123	santiago.gonzalez-martinez@inrae.fr
124	rene.graf@wsl.ch
125	alangray@ceh.ac.uk
126	dgrivet@inia.es
127	felix.gugerli@wsl.ch
128	hartleitner@sfl.at
129	Hollenb4@students.uni-marburg.de
130	agathe.hurel@inrae.fr
131	bernard.issenhuth@inrae.fr
132	florence.jean@inrae.fr
133	veronique.jorge@inrae.fr
134	arnaud.jouineau@inrae.fr
135	kappnerj@students.uni-marburg.de
136	katri.karkkainen@luke.fi
137	robert.kesalahti@oulu.fi
138	FAKnutzen@gmail.com
139	sonja.kujala@luke.fi
140	timo.kumpula@oulu.fi
141	mclabriola@gmail.com
142	celine.lalanne@inrae.fr
143	Lambertj@students.uni-marburg.de
144	martin.lascoux@ebc.uu.se
145	vincent.lejeune@inrae.fr
146	gregoire.le-provost@inrae.fr
147	joseph.levillain@inrae.fr
148	mirko.liesebach@thuenen.de
149	david.quiroga@csic.es
150	meier.benjamin@outlook.com
151	ermionimalliarou@yahoo.gr
152	jeremy.marchon@romandie.ch
153	nicolas.mariotte@inrae.fr
154	antonio.mas@mncn.csic.es

silvia.matesanzgarcia@gmail.com
helgem@posteo.de
celia.michotey@inrae.fr
pascal.milesi@scilifelab.uu.se
msandro@posteo.ch
daniel.nievergelt@wsl.ch
enotivol@cita-aragon.es
geir.ostreng@nibio.no
birte.pakull@thuenen.de
annt@ceh.ac.uk
andrea.piotti@ibbr.cnr.it
christophe.plomion@inrae.fr
nicolas.poinot@inrae.fr
mehdi.pringarbe@inrae.fr
luc.puzos@inrae.fr
tanja.pyhajarvi@oulu.fi
annie.raffin@inrae.fr
josealberto.ramirezvaliente@gmail.com
christian.rellstab@wsl.ch
remi.dourthe@inrae.fr
richte3d@staff.uni-marburg.de
jjrobledo@gmail.com
sergio.sansegundo@inia.es
outi.savolainen@oulu.fi
volker.schneck@thuenen.de
ivan.scotti@inrae.fr
semerikov@ipae.uran.ru
lenka.mateju@wsl.ch
jorn.sonstebo@nmbu.no
ilaria.spanu@ibbr.cnr.it
jean.thevenet@inrae.fr
Mari.Mette.Tollefsrud@nibio.no
Norbert.Turion@inrae.fr
giovanni.vendramin@ibbr.cnr.it
marc.villar@inrae.fr
georg.vonarx@wsl.ch
johan.westin@skogforsk.se
bruno.fady@inrae.fr
tor.myking@nibio.no
valladares@ccma.csic.es
aravanop@auth.gr
scav@ceh.ac.uk

Corresponding authors: opgenoorth@uni-marburg.de, benjamin.dauphin@wsl.ch, scav@ceh.ac.uk

Abstract

Background:

Progress in the field of evolutionary forest ecology has been hampered by the huge challenge of phenotyping trees across their ranges in their natural environments, and the limitation in high-resolution environmental information.

Findings:

The GenTree Platform contains phenotypic and environmental data from 4,959 trees from twelve ecologically and economically important European forest tree species: *Abies alba* Mill. (silver fir), *Betula pendula* Roth. (silver birch), *Fagus sylvatica* L. (European beech), *Picea abies* (L.) H. Karst (Norway spruce), *Pinus cembra* L. (Swiss stone pine), *Pinus halepensis* Mill. (Aleppo pine), *Pinus nigra* Arnold (European black pine), *Pinus pinaster* Aiton (maritime pine), *Pinus sylvestris* L. (Scots pine), *Populus nigra* L. (European black poplar), *Taxus baccata* L. (English yew), and *Quercus petraea* (Matt.) Liebl. (sessile oak). Phenotypic (height, diameter at breast height, crown-size, bark-thickness, biomass, straightness, forking, branch angle, fructification), regeneration, environmental in-situ measurements (soil depth, vegetation cover, competition indices), and environmental modeling data extracted by using bilinear interpolation accounting for surrounding conditions of each tree (precipitation, temperature, insolation, drought indices) were obtained from trees in 194 sites covering the species' geographic ranges and reflecting local environmental gradients.

Conclusion:

The GenTree Platform is a new resource for investigating ecological and evolutionary processes in forest trees. The coherent phenotyping and environmental characterization across 12 species in their European ranges allows for a wide range of analyses from forest ecologists, conservationists and macro-ecologists. In addition, the data here presented can be linked to the GenTree Dendroecological collection, the GenTree Leaf Trait collection, and the GenTree Genomic collection presented elsewhere, that together build the largest evolutionary forest ecology data collection available.

Measurements	vegetation cover • rock cover • soil depth • competition index • regeneration • diameter at breast height • height • crown size • bark thickness • number of fruits • stem straightness • branch angle • forking index
Technology Type(s)	bark gauges • calculations • caliper • clinometer • GPS device • increment corer • laser distance measurement • telescopic measuring pole
Factor Type(s)	tree species
Sample Characteristic Organism	*Abies alba* • *Betula pendula* • *Fagus sylvatica* • *Picea abies* • *Pinus cembra* • *Pinus halepensis* • *Pinus nigra* • *Pinus pinaster* • *Pinus sylvestris* • *Populus nigra* • *Taxus baccata* • *Quercus petraea*
Sample Characteristic Location	Europe

Machine-accessible metadata file describing the reported data:

Keywords

Regeneration, DBH, height, crown-size, bark-thickness, fruit-number, stem-straightness, branch-angle, forking-index, soil-depth

Context

The impacts of climate change and land-use change on forests are already severe, as observed, for example, following the extreme summer drought of 2018 that triggered a massive increase in mortality in Central European forests[1]. Furthermore, changes are expected to be acute in the future, altering distribution ranges and ecosystem functioning, as well as the interactions among species [2]. Forecasts indicate that near-surface temperature will shift poleward at mean rates of 80-430 m yr^{-1} for temperate forests during the 21st century [3]. This translates into northward shifts of trees' bioclimatic envelopes from 300 to 800 km within one century [3]. More importantly, the frequency and intensity of drought events, heat waves, forest fires and pest outbreaks [4] are expected to increase.

In the light of these changes, species and forest ecosystem resilience will depend on the extent and structure of phenotypic plasticity, genetic variation and adaptive potential, as well

as dispersal ability. From results of extensive networks of field experiments (provenance trials), it has long been shown that tree species are locally adapted at multiple spatial scales. In Europe, where most tree populations have established following post-glacial recolonization, such patterns of local adaptation must have developed rapidly and despite long generation time and extensive gene flow [5], a process enabled by high levels of within-population plasticity, genetic and epigenetic variation, and large population sizes [6]. Recent work has shown that genetic variation for stress response may be strongly structured along environmental gradients, such as water availability [7], temperature [8], or photoperiod [9]. However, the spatial patterns of current adaptation in particular phenotypic traits are only partly informative regarding the potential for future adaptation under a changing climate. To advance our understanding of the adaptive potential of trees, it is crucial to evaluate multiple traits in parallel to be able to model their putative response to new environmental conditions.

Recently, substantial effort has been made to identify specific genes and gene combinations that have undergone selection, by associating mutations at candidate loci with phenotypes related to stress events [10,11] or with environmental variables [12]. This latter example by Yeaman and co-workers [12] is one of the first association studies in forest tree species on a large genomic scale and the first to investigate convergent local adaptation in distantly related tree species. However, progress in this field has been hampered by limited genomic resources, the lack of small-scale, individual tree-level environmental information [13], and the huge challenge of phenotyping trees in their natural environments [14,15].

The GenTree Platform aims to address these challenges by providing individual level, high-resolution phenotypic and environmental data for a set of up to 20 sampling sites for each of twelve ecologically and economically important forest tree species across Europe. For a subset of seven species (*B. pendula*, *F. sylvatica*, *P. abies, P. pinaster*, *P. sylvestris*, *Populus nigra*, and *Q.petraea*), the sampling of sites was carried out in pairs, i.e. contained

two stands that were close enough to be connected by gene flow but situated in contrasting environments.

The sampling design described here was used for collecting phenotypic traits and ecological data. In addition, tree ring and wood density measurements for the same trees were assessed [16], and datasets on leaf traits, including specific leaf area (SLA) and isotopic content [17], and high-density single nucleotide polymorphism (SNP) data for each tree, were established, that will be published in GeneBank. All data and metadata information are gathered in the GnpIS repository (https://urgi.versailles.inra.fr/ephesis/ephesis/viewer.do#dataResults/trialSetIds=27) which makes updates possible[18].

We investigated the extent to which other datasets comparable to the data presented here exist by screening our twelve species in the TRY Plant Trait Data Base, the ITDRB, and the biomass and allometry data base for woody plants (BAAD). While this is a systematic approach, it leaves out a large number of tree species and therefore we cannot claim to have a comprehensive overview of the existing data. However, all three databases are large collections that include at least some of the tree measurements we present. Even though these are tremendous resources, the major difference is that based on their nature as collecting points of numerous independent datasets, there is no coherent sampling scheme in these collections as such, meaning that the number of trees per site, the way of tree selection, measured phenotypes, and provided environmental information vary greatly and therefore do not allow for coherent comparative analyses such as those of the GenTree Platform. For example, BAAD reports DBH data for only four of the species presented here, namely *Betula pendula* with three populations, *Fagus sylvatica* with two populations, *Picea abies* with four populations, and *Pinus sylvestris* with ten populations. In the larger TRY database, all of our species are represented, but the variability of sampling schemes is much more heterogenous in relation to traits, number of populations per species, and metadata.

For example, DBH measurements are being reported 232 times from a total of 12 *Betula pendula* populations. Of these the vast majority of the 170 measurements are from one population while from many other populations only one or up to five measurements are reported. Also, the measurements stem from five different original studies and thus having very different levels of additional information. We conclude that the core value of our reported data lies in the coherent sampling design and yet large number of sampled populations and individuals per species.

Methods

All recorded parameters are listed in Table 1.

Sampling strategy

To optimize the sampling design for genome scans and association studies, we followed the recent theoretical work by Lotterhos and Whitlock [19,20], which indicates that a paired sampling design has more power to detect the genomic signatures of local adaptation. Using this framework, populations from across the natural range of a species are sampled in pairs, with the two sites in each pair situated geographically close enough to be genetically similar at neutral genes due to a common evolutionary history and ongoing gene flow, but in distinct selective niches such that the local fitness optimum differs between the two sites. This sampling confers more power to detect evidence of selection in the genome through either association with environmental or phenotypic variables or the detection of outliers (e.g. for genetic differentiation, F_{ST}) (ibid.). Trees are very amenable to a pairwise approach since they are known to be locally adapted, often at fine spatial scales [21,22] and irrespective of gene flow distances [6]. This strategy was followed for a subset of seven species (see above) for which genomic resources were available (i.e. full or draft genome).

Such local niche contrasts are neither easy to identify nor readily available when environments are very homogenous. Therefore, a second principle of the sampling design

was to cover a large part of each species' natural geographic range (Fig. 1) and environmental space (Fig. 2) to capture selective niche variation. Finally, sites with a history of intensive management or any other intense and obvious anthropogenic or natural disturbances were avoided. This strategy was followed for all the 12 species.

Selection of trees on sites

A minimum of 25 trees were sampled per site to capture the natural phenotypic and genetic variability. Trees had to be mature but not senescent, dominant or codominant, and had to show no signs of significant damage due to pests and diseases or generally low vigor. Sampled trees were at least 30 meters apart and, where possible, were chosen along several parallel linear transects across each site, typically resulting in 2-4 transects per sampling site to keep the overall sampling area below 3 hectares.

Site and tree metadata

Sites were labeled by a two-letter country code (ISO 3166-1 alpha-2) followed by a two-letter species code and a two-digit site number (Table 1). Individual tree labels added another two-digit tree number. Every tree was permanently labeled so that future studies can resample subsets or the entire GenTree collection for gaining time-series data of individual traits or to add new phenotypes to the analyses. Be aware, that permission of the respective landowners must be obtained prior to sampling. Handheld GPS devices were used to record the position of each individual tree. The precision of GPS measurements in forests is notoriously challenging: regular commercial devices achieve an accuracy of about 8-15 m with good satellite coverage. Given that trees were selected with a minimum distance of 30 m this accuracy was sufficient for the correct positioning of trees relative to each other. An overall population position was defined by taking the mean value across all the individual tree measurements. Coordinates were in decimal degrees with 4 decimal units to reflect the general measurement accuracy (~11.1 m) and were stored in the WGS 84 reference system. GPS devices were also used to record the tree's elevation, either directly or through

post-hoc positioning in digital elevation models. The local aspect at the site of the tree was measured by compass in five-degree steps in the direction of the steepest slope.

The Metadata for each site consists of an ID code (see above), sampling date, location (GPS coordinates, see above) and elevation in meters above sea level (m a.s.l). Each stand was also characterized as being monospecific or mixed (in the latter case the most common co-occurring species was noted), stand structure was noted as single or multiple layered, and the age distribution as even or uneven (categorical variables).

Competition index at tree level

Competition indices were calculated following Canham et al. [23] and Lorimer et al. [24]. Specifically, the first index following Lorimer [24] was calculated as $NCI = \sum_{i=1}^{5}({DBH_j}/{DBH_i})/dist_i$ that follows the same notation as above, and where *DBH* is the *DBH* of the subject trees *j* and *i*.

Second, the distance-dependent competition index (NCI) following Canham et al. [23] was computed as $NCI = \sum_{i=1}^{5}({DBH_i}/{dist_i})$ where DBH_i is the diameter at breast height of competitor tree *i*, and $dist_i$ is the distance between the subject tree and competitor tree *i*. This index assumes that the net effects of neighboring trees vary as a direct function of the size of the neighbors and as an inverse function of the distance. For this purpose, the distance to the five nearest neighbors of each target tree was measured and their respective diameter at breast height was measured.

Moreover, it was noted whether competitor trees were conspecific to the target tree or not. Each multi-stemmed tree was considered as a single competitor where each stem larger than 15 cm DBH was measured and added to the sum of means.

Environmental characteristics within subplots around each tree

Surrounding each target tree, slope, vegetation cover (without tree cover), and stone content

were assessed in a 10 m x 10 m plot. The slope was assessed using a clinometer. Vegetation and rock cover were estimated in the classes <5%, 5-20%, 20-40%, 40-60%, 60-80%, 80-95%. Soil depth was estimated at three random points in the quadrat to a maximum of 60 cm with a pike and was averaged across these three values.

Regeneration

In the same 10 m x 10 m plots, natural regeneration of the target species was assessed according to the following four classes: absent (no recruit visible), scattered (few/scattered individuals), grouped (presence of scattered groups within the plot), and abundant (regularly spread all over the plot) and is indicated in the data base with values from 1-4. As this method cannot resolve maternity, the results indicate realized fecundity at the stand level.

Growth traits

DBH (cm)

DBH was measured at a stem height of 1.3 m using either a caliper by measuring two perpendicular diameters and subsequently taking the average of these two measurements or by measuring the circumference of the tree using a tape and computing the diameter from that value. Each measurement was performed to the nearest 0.1 cm. If a tree had more than one trunk, all of them were measured and the average was recorded.

Height (m)

Height from ground to the top of the crown was measured using a hypsometer (Nikon forestry Pro Laser), a laser vertex (Haglof Vertex III, Langsele, Sweden) or a Laser Range Meter (Bosch GLM 50 C, Leinfelden-Echterdingen, Germany). For short trees a telescopic measuring pole was used. Height was noted to the nearest 0.1 m. To forego errors introduced by measuring height on sloping ground, height measurements on slopes were conducted from the same elevation as the tree’s base by approaching the tree sideways. Where this was not possible, a slope correction factor was used.

Crown size (m²)

Crown size was measured as the circular and ellipsoid plane area of the crown. For this we measured two perpendicular crown diameters (canopy 1 and 2) by using a measurement tape, with the first measurement being made along the longest axis of the crown, from one edge to the other, and by visually projecting the crown margin onto the ground to the nearest decimeter. For the ellipse area we calculated $\left({}^{d_i}/_2\right) * \left({}^{d_j}/_2\right) * \pi$ and for the circular area $(d_i+d_j)/2*\pi$.

Bark thickness (mm)

For measuring bark thickness, we used bark gauges (Haglof Barktax) or a tape after extracting the bark with a small caliper (if bark could be detached without tree damage) or increment borers (Haglof increment borer) in case of strong and thick bark. Five measurements were taken for each tree at breast height and the average was calculated. For tree species with a clear dichotomy of bark thickness (e.g. old *Populus nigra*, *T. baccata*), we included at least two measurements from the thinner and thicker bark areas each.

Number of fruits (units)

In conifers, cones were counted by providing the average of three rounds of counting, made by an observer on the ground using binoculars. Only mature (brown) and closed cones were counted, i.e., those containing seeds, and not immature (green) or open cones, whose seeds had already been dispersed (open cones often stay on the branch for several years after seeds are dispersed). In broadleaves, the number of fruits was counted for 30 seconds, repeating the procedure three times to then average the three counts.

In case of species with very small fruits that are hard to see individually and in locations with a very limited view to the canopy, each tree was assigned to one of five categories, namely 0 (no fruits), 1 (a few fruits in a small section of the crown), 2 (a few fruits in two or more

sections of the crown), 3 (a lot of fruits in a small section of the crown), and 4 (a lot of fruits in two or more sections of the crown).

Straightness

Straightness of the stem was classified according to five levels: (1) No straight stem, (2) moderate or strong bends, (3) slight to moderate bend in different directions, (4) fairly straight (in one direction slightly crooked), (5) absolutely straight. This was performed on the lower 15 m of the tree beginning from the ground with the crown not taken into account. In the case of forked stems, only the trunk below the deepest forking point was evaluated.

Branch angle

Branch angle was classified at two successive whorls according to a five-scale scheme in conifers with (1) <23°, (2) 23-45°, (3) 45-67°, (4) 67°-90°, (5) > 90°, and a four-scale scheme in broadleaves omitting the >90° class. In black poplar, only the top two meters of the crown were considered.

Forking index

The branching of a tree in two (fork) or more (ramiform) equally thick and long stems was assessed with a forking index. The index took into account two parameters. First a score for the relative position of the fork: (4) no forking, (3) forking in the upper third of the tree, (2) forking in the middle third of the tree, (1) forking in the lower third of the tree; and second the number of axes (stems). The score of the relative position was then multiplied by ten and divided by the number of axes.

Modelled environmental data extracted for GenTree sites

Topography, soil, and climate data were compiled to characterize environmental conditions in each GenTree sampling site as follows.

Topography

We used the European digital elevation model to describe topographic conditions at 25 m spatial resolution with a vertical accuracy of about ± 7 meters (EU-DEM v. 1.1 from the Copernicus program; https://land.copernicus.eu/). We derived 14 variables (Table 2) based on biological hypotheses and their informative power at the local scale [25]. We calculated morphometric, hydrologic, and radiation grids for each GenTree site and visually inspected data integrity using SAGA 6.2 [26] (details in Table 2).

Soil

We collected available data on water capacity at seven soil depths using SoilGrids250m [27]. We estimated Pearson's correlation coefficients, *r*, between soil layers and then averaged the four first superficial (0, 5, 15, and 30 cm) and the three deeper (60, 100, and 200 cm) layers that were highly correlated, respectively.

Climate

We extracted climate data with high spatial resolution (30 arcsec) using CHELSA v. 1.2 [28]. CHELSA is based on a quasi-mechanistic statistical downscaling global reanalysis and global circulation model that, in particular, reliably interpolates the amount of precipitation using an orographic rainfall and wind effect. The dataset consisted of 48 climatic, 19 bioclimatic, four drought- and two frost-related variables for the reference period 1979-2013 (Table 2; http://chelsa-climate.org/bioclim/). We extracted all modeled environmental values for each individually geo-referenced tree using the *extract* function of the R package RASTER (Hijmans and van Etten 2016[29]). The surrounding conditions (i.e. adjacent pixels) of each individual tree were incorporated by the bilinear interpolation method when extracting the data.

Data Records

The data presented are structured in four independent csv files that can be merged using the site identifier (m04.site.id) or tree identifier (m06.tree.id). The same codes can be used to merge additional data namely from the GenTree Dendroecological Collection [16], the GenTree Leaf Trait Collection, and the GenTree Genomic Collection (personal communication). The first file contains the metadata descriptions, the second the individual phenotypic traits, the third the environmental data, and the fourth the modelled environmental data. These data files are available at figshare data repository open access under a CC0 license (https://figshare.com/s/4d57474fd63864a6dfd8). All the data are indexed in Table 2.

Table 1 Variables names, explanations, and specifications measured for all 4,959 trees and all 194 GenTree sites.

Variable name	Variable explanation	Specification
**GenTree Platform metadata**		
m01.spec	Species abbreviations	*Abies Alba* [AA], *Betula pendula* [BP], *Fagus sylvatica* [FS], *Picea abies* [PA], *Pinus cembra* [PC], *Pinus halepensis* [PH], *Pinus nigra* [PN], *Populus nigra* [PO], *Pinus pinaster* [PP], *Pinus sylvestris* [PS], *Quercus petraea* [QP], *Taxus baccata* [TB]
m02.country	Country abbreviations	Isocode 6133-2; Austria [AT], Switzerland [CH], Germany [DE], Spain [ES], Finland [FI], France [FR], Great Britain [GB], Greece [GR], Italy [IT], Lithuania [LT], Norway [NO], Sweden [SE]
m03.site.num	Site numbers	Running numbers of sites per species 01-24
m04.site.id	Complete site-ID per species	Merger of m01-m03
m05.tree.num	Tree numbers	Running numbers within sites 01-25
m06.tree.id	Complete tree ID	Merger of m01-m03, m05
m07.trial.name	Site name	
m08.lat	Latitude	Decimal degrees, WGS84
m09.lon	Longitude	Decimal degrees, WGS84
**GenTree Platform phenotypes**		
p01.height	Height	Tree height, m
p02.dbh	DBH	Diameter at breast height, cm
p03.bark	Bark thickness mean	Mean value of bark thickness, cm
p04.trunk	Trunk straightness/flexuosity	5: absolutely straight, 4: fairly straight (in one direction slightly crooked), 3: slight to moderate bend in different directions, 2: moderate or strong bends, 1: no straight stem
p05.branch	Branch angle	1: <23° (steep), 2: 23° - 45°, 3: 45º-67º, 4: 67º-90º (plain), 5: >90º
p06.fork	Forking index	1: fork at the lower third of tree height, 2: fork at middle third, 3: fork at upper third, 4: no fork – multiplied by 10 and then divided by the number of stems
p07.canopy.1	Canopy projection REP 1	Crown diameter projection, m
p08.canopy.2	Canopy projection REP 2	Crown diameter projection, m
p09.crown.ellipse	Crown ellipse	Area of an ellipse $(d1/2)*(d2/2)*\pi$, m^2
p10.crown.round	Crown size	As some only have one diameter, round areas with the mean diameter $((D1+D2/2)/2)^2*\pi$, m^2
p11.regeneration	Natural regeneration	1: absent, 2: scattered, 3: groups, 4: abundant
p12.fruit.mean	Fruit/cone number	Number of fruits.
p13.basal.area		

GenTree Platform in-situ environmental measurements		
e01.plant.cover	Total plant cover	1: none, 2: little (5-20%), 3: low (20-40%), 4: medium (40-60%), 5: high (60-80%), 6: very high (80-95%), 7: full cover (>95%)
e02.comp.index.a	Competition index A	CI assessed following Canham et al 2004, and multi-stems as the sum
e03.comp.index.b	Competition index B	CI assessed following Canham et al 2004, and multi-stems assessing the sum of basal areas and then the DBH
e04.comp.index.c	Competition index C	CI assessed following Lorimer 1983, and multi-stems as the sum
e05.comp.index.d	Competition index D	CI assessed following Lorimer 1983, multi-stems assessing the sum of basal areas and then the DBH
e06.status		Dominant, co-dominant
e07.elevation	Elevation of the tree	Meters above sea level
e08.slope	Slope at the tree level	Slope in degrees
e09.aspect	Aspect at the tree level	0-360°
e10.soil.depth	Mean soil depth	Mean of 3 measures (measurement to a max. depth of 60 cm
e11.stone.content	Mean stone content	Mean of 3 measures (1: none, 2: little (5-20%), 3: low (20-40%), 4: medium (40-60%), 5: high (60-80%), 6: very high (80-95%), 7: full cover (>95%)
e12.rock.cover	Total rock cover	1: none, 2: little (5-20%), 3: low (20-40%), 4: medium (40-60%), 5: high (60-80%), 6: very high (80-95%), 7: full cover (>95%)

Table 2 Environmental variable names, explanations, and specifications modellled for all 4959 trees and 194 GenTree sites.

Variable name	Variable explanation	Specification
**GenTree Platform modelled environmental parameters**		
sample	Sample identification	unit: none; resolution [m]: none
country	Country code	unit: none; resolution [m]: none
countryspecies	Country and species code	unit: none; resolution [m]: none
species	Species code	unit: none; resolution [m]: none
population	Population identification	unit: none; resolution [m]: none
latwgs84	Latitude in WGS84	unit: degree; resolution [m]: 25
lonwgs84	Longitude in WGS84	unit: degree; resolution [m]: 25
latetrs89	Latitude in ETRS89	unit: degree; resolution [m]: 25
lonetrs89	Longitude in ETRS89	unit: degree; resolution [m]: 25
t01alt	Altitude	unit: m; resolution [m]: 25
t02slp	Slope	unit: degree; resolution [m]: 25
t03asp	Eastness	unit: degree; resolution [m]: 25
t04vcu	Profile curvature	unit: degree/m; resolution [m]: 25
t05hcu	Horizontal curvature	unit: degree/m; resolution [m]: 25
t06ddg	Downslope distance gradient	unit: degree; resolution [m]: 25
t07mpi	Morphometric protection index	unit: none; resolution [m]: 25
t08tpi	Topographic position index	unit: none; resolution [m]: 25
t09vrm	Vector ruggedness measure	unit: none; resolution [m]: 25
t10twi	Topographic wetness index	unit: none; resolution [m]: 25
t11svf	Sky-view factor	unit: none; resolution [m]: 25
t12sdir	Potential direct solar radiation	unit: kJ/m^2; resolution [m]: 25
t13sdif	Potential diffuse solar radiation	unit: kJ/m^2; resolution [m]: 25
t14stot	Potential total solar radiation	unit: kJ/m^2; resolution [m]: 25
awc15	Available water capacity (0-30cm)	unit: %; resolution [m]: 250
awc140	Available water capacity (60-200cm)	unit: %; resolution [m]: 250
bio01	Yearly mean temperature	unit: °C/10; resolution [m]: 1000
bio02	Mean diurnal range	unit: °C/10; resolution [m]: 1000
bio03	Isothermality	unit: °C/10; resolution [m]: 1000
bio04	Temperature seasonality	unit: °C/10; resolution [m]: 1000
bio05	Max temperature of warmest month	unit: °C/10; resolution [m]: 1000
bio06	Min temperature of coldest month	unit: °C/10; resolution [m]: 1000
bio07	Temperature annual range	unit: °C/10; resolution [m]: 1000
bio08	Mean temperature of wettest quarter	unit: °C/10; resolution [m]: 1000
bio09	Mean temperature of driest quarter	unit: °C/10; resolution [m]: 1000
bio10	Mean temperature of warmest quarter	unit: °C/10; resolution [m]: 1000
bio11	Mean temperature of coldest quarter	unit: °C/10; resolution [m]: 1000
bio12	Yearly precipitation sum	unit: kg m^{-2}; resolution [m]: 1000
bio13	Precipitation of wettest month	unit: kg m^{-2}; resolution [m]: 1000
bio14	Precipitation of driest month	unit: kg m^{-2}; resolution [m]: 1000
bio15	Precipitation seasonality	unit: kg m^{-2}; resolution [m]: 1000
bio16	Precipitation of wettest quarter	unit: kg m^{-2}; resolution [m]: 1000
bio17	Precipitation of driest quarter	unit: kg m^{-2}; resolution [m]: 1000
bio18	Precipitation of warmest quarter	unit: kg m^{-2}; resolution [m]: 1000
bio19	Precipitation of coldest quarter	unit: kg m^{-2}; resolution [m]: 1000
gdd	Growing degree days	unit: °C; resolution [m]: 1000
gsp	Accumulated precipitation	unit: kg m^{-2}; resolution [m]: 1000
shc	Hydrothermic coefficient	unit: (kg m^{-2}/10)/°C; resolution [m]: 1000
rh410	Relative humidity	unit: %; resolution [m]: 1000
fcf	Frost change frequency	unit: number of events; resolution [m]: 1000
nfd	Number of frost days	unit: number of days; resolution [m]: 1000
prec01	Precipitation sum in January	unit: kg m^{-2}; resolution [m]: 1000
prec02	Precipitation sum in February	unit: kg m^{-2}; resolution [m]: 1000
prec03	Precipitation sum in March	unit: kg m^{-2}; resolution [m]: 1000
prec04	Precipitation sum in April	unit: kg m^{-2}; resolution [m]: 1000
prec05	Precipitation sum in May	unit: kg m^{-2}; resolution [m]: 1000
prec06	Precipitation sum in June	unit: kg m^{-2}; resolution [m]: 1000
prec07	Precipitation sum in July	unit: kg m^{-2}; resolution [m]: 1000
prec08	Precipitation sum in August	unit: kg m^{-2}; resolution [m]: 1000
prec09	Precipitation sum in September	unit: kg m^{-2}; resolution [m]: 1000
prec10	Precipitation sum in October	unit: kg m^{-2}; resolution [m]: 1000
prec11	Precipitation sum in November	unit: kg m^{-2}; resolution [m]: 1000
prec12	Precipitation sum in December	unit: kg m^{-2}; resolution [m]: 1000
tmean01	Mean temperature in January	unit: °C/10; resolution [m]: 1000

tmean02	Mean temperature in February	unit: °C/10; resolution [m]: 1000
tmean03	Mean temperature in March	unit: °C/10; resolution [m]: 1000
tmean04	Mean temperature in April	unit: °C/10; resolution [m]: 1000
tmean05	Mean temperature in May	unit: °C/10; resolution [m]: 1000
tmean06	Mean temperature in June	unit: °C/10; resolution [m]: 1000
tmean07	Mean temperature in July	unit: °C/10; resolution [m]: 1000
tmean08	Mean temperature in August	unit: °C/10; resolution [m]: 1000
tmean09	Mean temperature in September	unit: °C/10; resolution [m]: 1000
tmean10	Mean temperature in October	unit: °C/10; resolution [m]: 1000
tmean11	Mean temperature in November	unit: °C/10; resolution [m]: 1000
tmean12	Mean temperature in December	unit: °C/10; resolution [m]: 1000
tmin01	Minimum temperature in January	unit: °C/10; resolution [m]: 1000
tmin02	Minimum temperature in February	unit: °C/10; resolution [m]: 1000
tmin03	Minimum temperature in March	unit: °C/10; resolution [m]: 1000
tmin04	Minimum temperature in April	unit: °C/10; resolution [m]: 1000
tmin05	Minimum temperature in May	unit: °C/10; resolution [m]: 1000
tmin06	Minimum temperature in June	unit: °C/10; resolution [m]: 1000
tmin07	Minimum temperature in July	unit: °C/10; resolution [m]: 1000
tmin08	Minimum temperature in August	unit: °C/10; resolution [m]: 1000
tmin09	Minimum temperature in September	unit: °C/10; resolution [m]: 1000
tmin10	Minimum temperature in October	unit: °C/10; resolution [m]: 1000
tmin11	Minimum temperature in November	unit: °C/10; resolution [m]: 1000
tmin12	Minimum temperature in December	unit: °C/10; resolution [m]: 1000
tmax01	Maximum temperature in January	unit: °C/10; resolution [m]: 1000
tmax02	Maximum temperature in February	unit: °C/10; resolution [m]: 1000
tmax03	Maximum temperature in March	unit: °C/10; resolution [m]: 1000
tmax04	Maximum temperature in April	unit: °C/10; resolution [m]: 1000
tmax05	Maximum temperature in May	unit: °C/10; resolution [m]: 1000
tmax06	Maximum temperature in June	unit: °C/10; resolution [m]: 1000
tmax07	Maximum temperature in July	unit: °C/10; resolution [m]: 1000
tmax08	Maximum temperature in August	unit: °C/10; resolution [m]: 1000
tmax09	Maximum temperature in September	unit: °C/10; resolution [m]: 1000
tmax10	Maximum temperature in October	unit: °C/10; resolution [m]: 1000
tmax11	Maximum temperature in November	unit: °C/10; resolution [m]: 1000
tmax12	Maximum temperature in December	unit: °C/10; resolution [m]: 1000

The local environmental contrasts varied among species and population pairs, most of which exhibited variability with respect to elevation, temperature, precipitation, and water availability. Other local contrasts were based on radiation, soil water capacity, and topographic wetness index (among others). One special case is *Populus nigra*, a heliophilous pioneer species found naturally in riverine areas. Given this specific habitat, local contrasts were largely bound to the distance of the individual trees from the riverbed and thus for example to ground water access or exposure to variation in the intensity and frequency of floods.

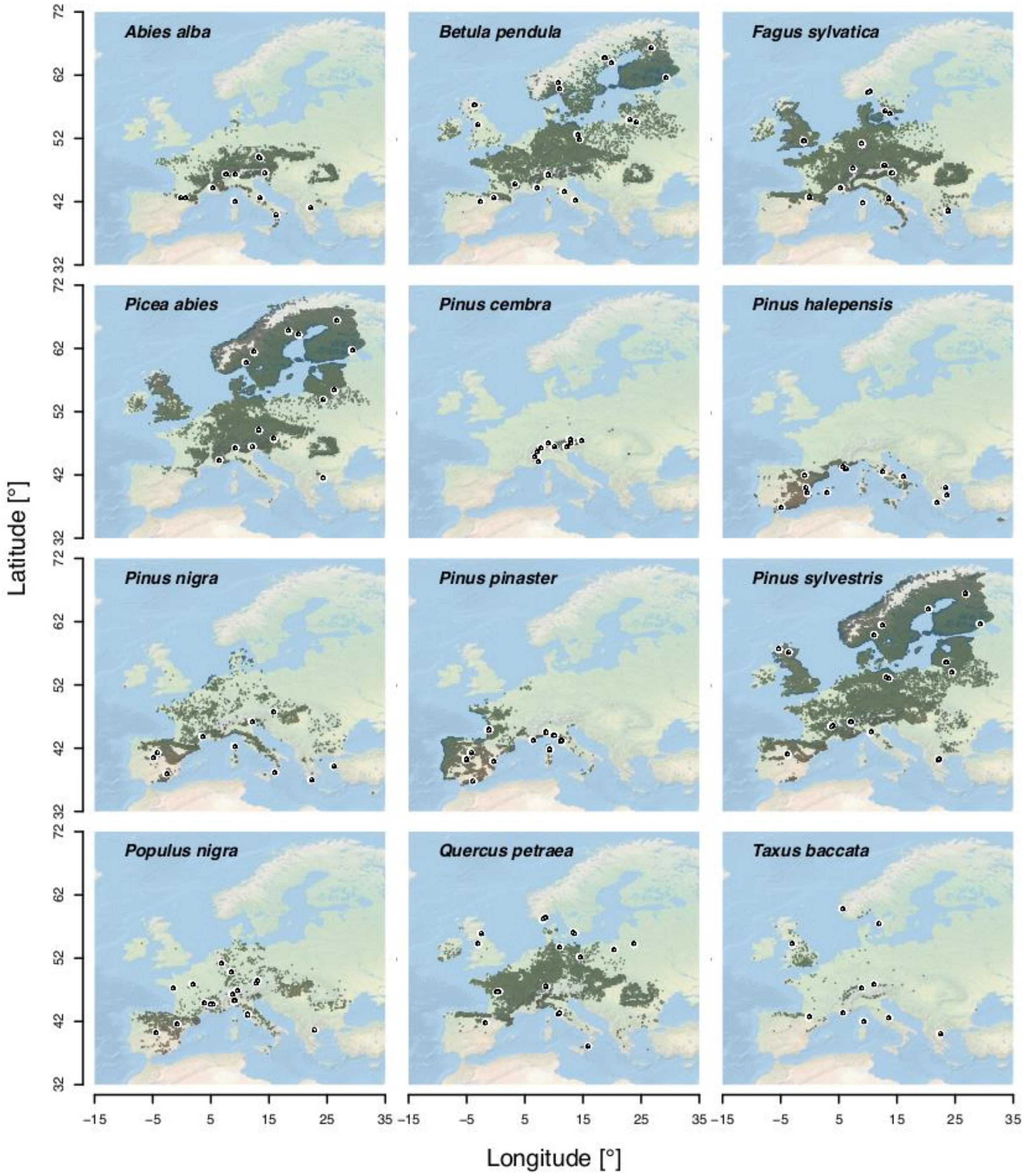

Fig. 1 Sampling sites (black dots) and distributions of the twelve selected tree species (dark green shading) for *in-situ* phenotype measurements. Distribution maps are based on a comprehensive high-resolution tree occurrence dataset from the European Union [30].

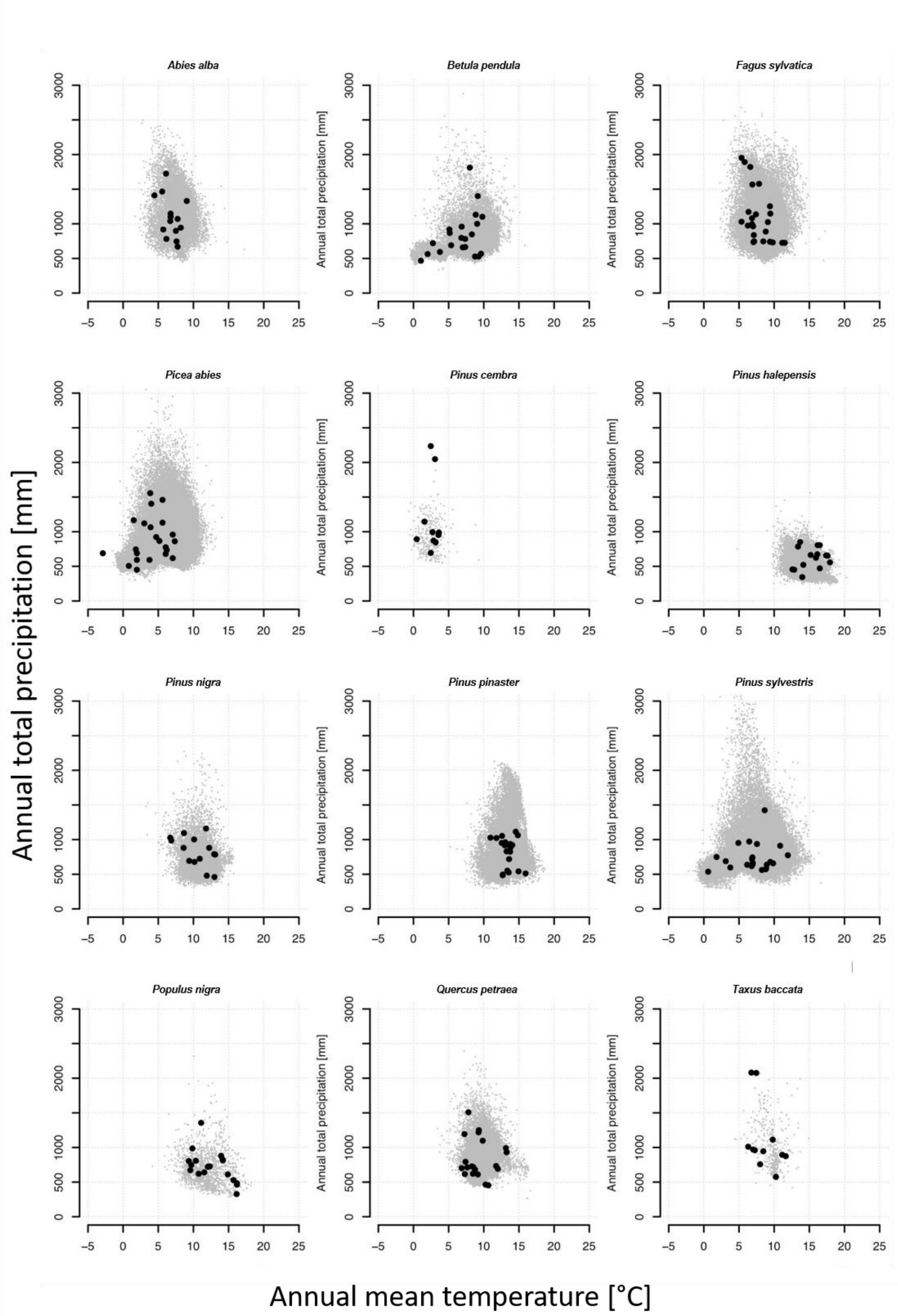

Fig. 2 Climate-space diagrams for the 12 selected European tree species with annual mean temperature on the x-axis and annual total precipitation on the y-axis. Grey points represent species occurrences based on a comprehensive high-resolution tree occurrence dataset for Europe [30] and black dots indicate the GenTree sites.

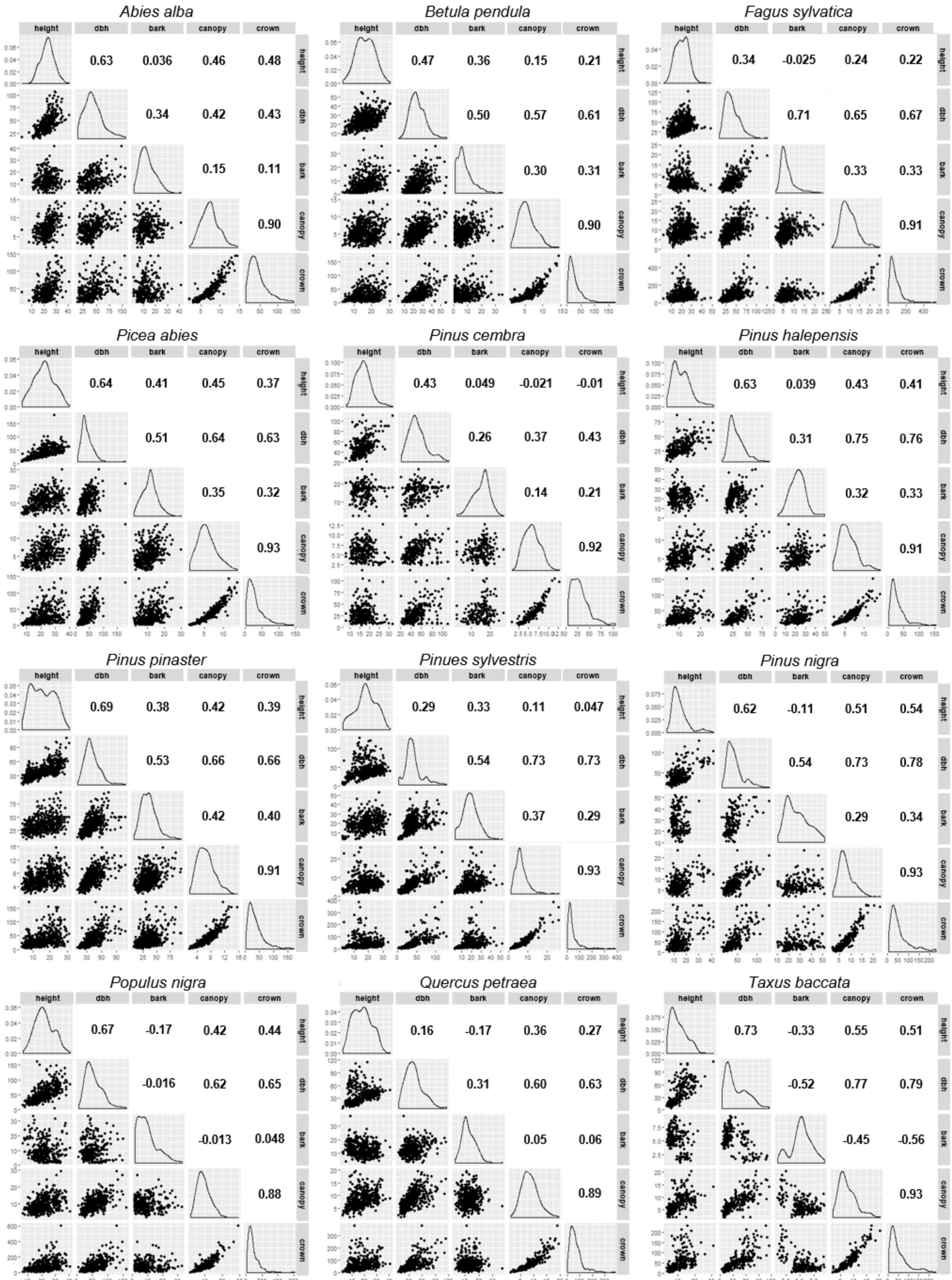

Fig. 3 Scatterplots, distributions and Pearson's correlation coefficients, *r*, of GenTree phenotype measurements in the 12 selected European tree species.

Data validation and quality control

The database has been checked for consistency at different stages by various researchers between 2018-2020. Raw data were submitted by all partners to the GnpIS multispecies integrative information system (https://urgi.versailles.inra.fr/Tools/GnpIS) using preformatted Microsoft Excel templates. Data files were harmonized, merged and subsequently verified following several steps:

1. Missing data and dubious entries were checked manually by examining the original data files obtained from the partners and by cross-checking cases with field books.
2. Descriptive statistics were calculated and plotted for all variables including minima, maxima, means, and variances. Outliers were checked against original data records, and corrected when necessary.
3. Covariables were plotted determining whether relationships were reasonable and following the most complete set of similar relationships (Fig. 3).

Acknowledgements¶

We would like to thank Juri Nievergelt, Anne Verstege, Marina Fonti, Kevin Kleeb, Frederick Reinig, Enrica Zalloni, Claudio de Sassi, Giacomo Poli for their field assistance. This work was performed using GnpIS, one of the facilities of the URGI platform (https://urgi.versailles.inra.fr/). We are also grateful to all the forest owners and national administrations for providing sampling permissions.

Author contributions¶

LO, RB, KH, BF, TM, FV, FAA, SC coordinated sampling design.

All authors contributed to sampling design

LO, RB, KH, BF, TM, FV, FAA, SC coordinated field sampling.¶

All authors contributed to the field sampling.

CM, MB compiled and assembled in-situ measurements in the GnpIS database.¶

BD extracted climatic and topographic data and derived environmental indices for all the sampling sites.¶

RB, LO, BD, PAA, CM curated data, checked quality, and prepared the data sets with metadata descriptions for sharing and potential reuse.¶

LO, KH, BD, SC, BF wrote the manuscript.¶

BF coordinated GenTree

All authors commented on an earlier version and approved the current version of the manuscript.

Competing interests¶

The authors declare no competing interest.

Funding

This publication is part of the GenTree project which was funded by the European Union's Horizon 2020 research and innovation program under grant agreement No. 676876 (GenTree). This work was also supported by the Swiss Secretariat for Education, Research and Innovation (SERI) under contract No. 6.0032.

References

1. Schuldt, B. *et al.* A first assessment of the impact of the extreme 2018 summer drought on Central European forests. *Basic and Applied Ecology* **45,** 86–103; 10.1016/j.baae.2020.04.003 (2020).
2. Sitch, S. *et al.* Evaluation of the terrestrial carbon cycle, future plant geography and climate-carbon cycle feedbacks using five Dynamic Global Vegetation Models (DGVMs). *Global Change Biology* **14,** 2015–2039; 10.1111/j.1365-2486.2008.01626.x (2008).
3. Loarie, S. R. *et al.* The velocity of climate change. *Nature* **462,** 1052–1055; 10.1038/nature08649 (2009).
4. Pachauri, R. K. & Mayer, L. (eds.). *Climate change 2014. Synthesis report* (Intergovernmental Panel on Climate Change, Geneva, Switzerland, 2015).
5. Savolainen, O., Pyhäjärvi, T. & Knürr, T. Gene Flow and Local Adaptation in Trees. *Annu. Rev. Ecol. Evol. Syst.* **38,** 595–619; 10.1146/annurev.ecolsys.38.091206.095646 (2007).
6. Kremer, A. *et al.* Long-distance gene flow and adaptation of forest trees to rapid climate change. *Ecology Letters* **15,** 378–392; 10.1111/j.1461-0248.2012.01746.x (2012).
7. Eveno, E. *et al.* Contrasting patterns of selection at *Pinus pinaster* Ait. Drought stress candidate genes as revealed by genetic differentiation analyses. *Molecular Biology and Evolution* **25,** 417–437; 10.1093/molbev/msm272 (2008).
8. Holliday, J. A., Ritland, K. & Aitken, S. N. Widespread, ecologically relevant genetic markers developed from association mapping of climate-related traits in Sitka spruce (Picea sitchensis). *New Phytologist* **188,** 501–514; 10.1111/j.1469-8137.2010.03380.x (2010).
9. Chen, J. *et al.* Clinal variation at phenology-related genes in spruce: parallel evolution in FTL2 and Gigantea? *Genetics* **197,** 1025–1038; 10.1534/genetics.114.163063 (2014).
10. González-Martínez, S. C., Ersoz, E., Brown, G. R., Wheeler, N. C. & Neale, D. B. DNA sequence variation and selection of tag single-nucleotide polymorphisms at candidate genes for drought-stress response in *Pinus taeda* L. *Genetics* **172,** 1915–1926; 10.1534/genetics.105.047126 (2006).
11. González-Martínez, S. C., Krutovsky, K. V. & Neale, D. B. Forest-tree population genomics and adaptive evolution. *New Phytologist* **170,** 227–238; 10.1111/j.1469-8137.2006.01686.x (2006).
12. Yeaman, S. *et al.* Convergent local adaptation to climate in distantly related conifers. *Science (New York, N.Y.)* **353,** 1431–1433; 10.1126/science.aaf7812 (2016).
13. Scotti, I., González-Martínez, S. C., Budde, K. B. & Lalagüe, H. Fifty years of genetic studies: what to make of the large amounts of variation found within populations? *Annals of Forest Science* **73,** 69–75; 10.1007/s13595-015-0471-z (2016).
14. Alexandre, H. *et al.* In situ estimation of genetic variation of functional and ecological traits in *Quercus petraea* and *Q.robur* (2018).
15. Dungey, H. S. *et al.* Phenotyping Whole Forests Will Help to Track Genetic Performance. *Trends in Plant Science* **23,** 854–864; 10.1016/j.tplants.2018.08.005 (2018).

16. Martínez-Sancho, E. *et al.* The GenTree Dendroecological Collection, tree-ring and wood density data from seven tree species across Europe. *Scientific Data* **7,** 1; 10.1038/s41597-019-0340-y (2020).

17. Raquel Benavides *et al.* The GenTree Leaf Collection: inter- and intraspecific leaf variation in seven forest tree species in Europe. *Global Ecology and Biogeography*; 10.1111/geb.13239.

18. Steinbach, D. *et al.* GnpIS: an information system to integrate genetic and genomic data from plants and fungi. *Database: the journal of biological databases and curation* **2013,** bat058; 10.1093/database/bat058 (2013).

19. Lotterhos, K. E. & Whitlock, M. C. Evaluation of demographic history and neutral parameterization on the performance of FST outlier tests. *Molecular Ecology* **23,** 2178–2192; 10.1111/mec.12725 (2014).

20. Lotterhos, K. E. & Whitlock, M. C. The relative power of genome scans to detect local adaptation depends on sampling design and statistical method. *Molecular Ecology* **24,** 1031–1046; 10.1111/mec.13100 (2015).

21. Eckert, A. J. *et al.* Back to nature: ecological genomics of loblolly pine (*Pinus taeda*, Pinaceae). *Molecular Ecology* **19,** 3789–3805; 10.1111/j.1365-294X.2010.04698.x (2010).

22. Lind, B. M. *et al.* Water availability drives signatures of local adaptation in whitebark pine (*Pinus albicaulis* Engelm.) across fine spatial scales of the Lake Tahoe Basin, USA. *Molecular Ecology* **26,** 3168–3185; 10.1111/mec.14106 (2017).

23. Canham, C. D., LePage, P. T. & Coates, K. D. A neighborhood analysis of canopy tree competition: effects of shading versus crowding. *Can. J. For. Res.* **34,** 778–787; 10.1139/x03-232 (2004).

24. Lorimer, C. G. Tests of age-independent competition indices for individual trees in natural hardwood stands. *Forest Ecology and Management* **6,** 343–360 (1983).

25. Leempoel, K. *et al.* Very high-resolution digital elevation models: are multi-scale derived variables ecologically relevant? *Methods Ecol Evol* **6,** 1373–1383; 10.1111/2041-210X.12427 (2015).

26. Conrad, O. *et al.* System for Automated Geoscientific Analyses (SAGA) v. 2.1.4. *Geosci. Model Dev. Discuss.* **8,** 2271–2312; 10.5194/gmdd-8-2271-2015 (2015).

27. Hengl, T. *et al.* Mapping Soil Properties of Africa at 250 m Resolution: Random Forests Significantly Improve Current Predictions. *PLoS ONE* **10,** e0125814; 10.1371/journal.pone.0125814 (2015).

28. Karger, D. N. *et al.* Climatologies at high resolution for the earth's land surface areas. *Scientific Data* **4,** 170122; 10.1038/sdata.2017.122 (2017).

29. Hijmans, R. & van Etten, J. *raster: Geographic data analysis and modeling. R package* (2016).

30. Mauri, A., Strona, G. & San-Miguel-Ayanz, J. EU-Forest, a high-resolution tree occurrence dataset for Europe. *Scientific Data* **4,** 160123; 10.1038/sdata.2016.123 (2017).

Click here to access/download
Supplementary Material
Opgenoorth et al
2020_resubmission_changes_marked.docx

Personal Cover
Click here to access/download;Personal Cover;Opgenoorth et al 2020 - Letter to the Editor.pdf

Plant Ecology and Geobotany
Prof. Dr. Lars Opgenoorth
Karl-von-Frisch Strasse 8 | D-35043 Marburg
opgenoorth@uni-marburg.de | +49 6421 2822080

Philipps Universität Marburg

Dear Editor,

Thank you very much for giving us the chance to submit a revision of our manuscript “The GenTree Platform: growth traits and tree-level environmental data in twelve European forest tree species.

We found the reviewer comments to be very helpful and have changed the text, data and data presentation accordingly.

As you requested, we payed particular attention to reviewer 1’s points regarding sampling methodology and data structure. Most notably, we added the sampling dates for the respective measurements in the data frame as requested by reviewer 1 and we have added a paragraph where we describe how we looked into three important databases namely the TRY plant trait database, the International Tree Ring Data Bank (ITRDB), and the biomass and allometry data base for woody plants (BAAD) to support our claim that we provide a unique dataset also as requested by Reviewer 1. Also, we explained how repeated measurements are not part of this data paper but are enabled by the permanent marking of trees and are planned already for a subset in a European H2020 follow-up project.

We hope that you and the reviewers will find our manuscript and data worthy for publication.

Thank you very much

Opgenoorth

Lars Opgenoorth
