# Supplementary material for: The GenTree Platform: growth traits and tree-level environmental data in 12 European forest tree species
Source: Gigascience. 2021 Mar 18;10(3):giab010. doi: 10.1093/gigascience/giab010 (PMC7970660; doi:10.1093/gigascience/giab010)
Supplement: giab010_GIGA-D-20-00189_Revision_2 [file giab010_giga-d-20-00189_revision_2.pdf]

GigaScience

The GenTree Platform: growth traits and tree-level environmental data in twelve European forest tree species

--Manuscript Draft--

**Manuscript Number:**	GIGA-D-20-00189R2
**Full Title:**	The GenTree Platform: growth traits and tree-level environmental data in twelve European forest tree species
**Article Type:**	Data Note
**Funding Information:**	Horizon 2020 Framework Programme (676876) — Mr. Bruno Fady<br>Staatssekretariat für Bildung, Forschung und Innovation (6.0032) — Mr. Felix Gugerli
**Abstract:**	Background:<br>Progress in the field of evolutionary forest ecology has been hampered by the huge challenge of phenotyping trees across their ranges in their natural environments, and the limitation in high-resolution environmental information.<br>Findings:<br>The GenTree Platform contains phenotypic and environmental data from 4,959 trees from twelve ecologically and economically important European forest tree species: Abies alba Mill. (silver fir), Betula pendula Roth. (silver birch), Fagus sylvatica L. (European beech), Picea abies (L.) H. Karst (Norway spruce), Pinus cembra L. (Swiss stone pine), Pinus halepensis Mill. (Aleppo pine), Pinus nigra Arnold (European black pine), Pinus pinaster Aiton (maritime pine), Pinus sylvestris L. (Scots pine), Populus nigra L. (European black poplar), Taxus baccata L. (English yew), and Quercus petraea (Matt.) Liebl. (sessile oak). Phenotypic (height, diameter at breast height, crown-size, bark-thickness, biomass, straightness, forking, branch angle, fructification), regeneration, environmental in-situ measurements (soil depth, vegetation cover, competition indices), and environmental modeling data extracted by using bilinear interpolation accounting for surrounding conditions of each tree (precipitation, temperature, insolation, drought indices) were obtained from trees in 194 sites covering the species' geographic ranges and reflecting local environmental gradients.<br>Conclusion:<br>The GenTree Platform is a new resource for investigating ecological and evolutionary processes in forest trees. The coherent phenotyping and environmental characterization across 12 species in their European ranges allows for a wide range of analyses from forest ecologists, conservationists and macro-ecologists. In addition, the data here presented can be linked to the GenTree Dendroecological collection, the GenTree Leaf Trait collection, and the GenTree Genomic collection presented elsewhere, that together build the largest evolutionary forest ecology data collection available.
**Corresponding Author:**	Lars Opgenoorth<br>Philipps-Universität Marburg<br>Marburg, Hessen GERMANY
**Corresponding Author Secondary Information:**	
**Corresponding Author's Institution:**	Philipps-Universität Marburg
**Corresponding Author's Secondary Institution:**	
**First Author:**	Lars Opgenoorth
**First Author Secondary Information:**	
**Order of Authors:**	Lars Opgenoorth<br>Benjamin Dauphin

Powered by Editorial Manager® and ProduXion Manager® from Aries Systems Corporation

	Raquel Benavides
	Katrin Heer
	Paraskevi Alizoti
	Elisabet Martínez-Sancho
	Ricardo Alía
	Olivier Ambrosio
	Albet Audrey
	Camilla Avanzi
	Evangelia Avramidou
	Francesca Bagnoli
	Evangelos Barbas
	Cristina C. Bastias
	Catherine Bastien
	Eduardo Ballesteros
	Giorgia Beffa
	Frédéric Bernier
	Henri Bignalet
	Guillaume Bodineau
	Damien Bouic
	Sabine Brodbeck
	William Brunetto
	Jurata Buchovska
	Melanie Buy
	Ana M. Cabanillas-Saldaña
	Bárbara Carvalho
	Nicolas Cheval
	José M. Climent
	Marianne Correard
	Eva Cremer
	Darius Danusevičius
	Fernando Del Caño
	Jean-Luc Denou
	Nicolas di Gerardi
	Bernard Dokhelar
	Alexis Ducousso
	Eskild Nilsen
	Anna-Maria Farsakoglou
	Patrick Fonti
	Ioannis Ganopoulos
	José M. García del Barrio
	Olivier Gilg

Powered by Editorial Manager® and ProduXion Manager® from Aries Systems Corporation

	Santiago C. González-Martínez
	René Graf
	Alan Gray
	Delphine Grivet
	Felix Gugerli
	Christoph Hartleitner
	Enja Hollenbach
	Agathe Hurel
	Bernard Issehut
	Florence Jean
	Veronique Jorge
	Arnaud Jouineau
	Jan-Philipp Kappner
	Katri Kärkkäinen
	Robert Kesälahti
	Florian Knutzen
	Sonja Kujala
	Timo Kumpula
	Mariaceleste Labriola
	Celine Lalanne
	Johannes Lambertz
	Martin Lascoux
	Vincent Lejeune
	Gregoire Le-Provost
	Joseph Levillain
	Mirko Liesebach
	David López-Quiroga
	Benjamin Meier
	Ermioni Malliarou
	Jérémy Marchon
	Nicolas Mariotte
	Antonio Mas
	Silvia Matesanz
	Helge Meischner
	Célia Michotey
	Pascal Milesi
	Sandro Morganti
	Daniel Nievergelt
	Eduardo Notivol
	Geir Ostreng
	Birte Pakull

Powered by Editorial Manager® and ProduXion Manager® from Aries Systems Corporation

	Annika Perry
	Andrea Piotti
	Christophe Plomion
	Nicolas Poinot
	Mehdi Pringarbe
	Luc Puzos
	Tanja Pyhäjärvi
	Annie Raffin
	José A. Ramírez-Valiente
	Christian Rellstab
	Dourthe Remi
	Sebastian Richter
	Juan J. Robledo-Arnuncio
	Sergio San Segundo
	Outi Savolainen
	Volker Schneck
	Ivan Scotti
	Vladimir Semerikov
	Lenka Slámová
	Jørn Henrik Sønstebø
	Ilaria Spanu
	Jean Thevenet
	Mari Mette Tollefsrud
	Norbert Turion
	Giovanni Giuseppe Vendramin
	Marc Villar
	Georg Von Arx
	Johan Westin
	Bruno Fady
	Tor Myking
	Fernando Valladares
	Filippos A. Aravanopoulos
	Stephen Cavers
**Order of Authors Secondary Information:**	
**Response to Reviewers:**	1. I have included the corrected author list<br>2. I citing the GigaDB repository. However, since the doi is not yet availabl, I followed this advise by Chris Hunter: "Thank you, I will process the dataset as soon as possible. If that is the only bit of information you require to complete the manuscript submission then there is no need for you to wait, the handling editor (CC'd) can insert the dataset citation on your behalf during the copy editing stage."<br>3. I send a brief explanation via email regarding the additional author.<br>4. The additional author fits under the contribution listed as was in place.

Powered by Editorial Manager® and ProduXion Manager® from Aries Systems Corporation

Additional Information:	
Question	Response
Are you submitting this manuscript to a special series or article collection?	No
**Experimental design and statistics**<br><br>Full details of the experimental design and statistical methods used should be given in the Methods section, as detailed in our Minimum Standards Reporting Checklist. Information essential to interpreting the data presented should be made available in the figure legends.<br><br>Have you included all the information requested in your manuscript?	Yes
**Resources**<br><br>A description of all resources used, including antibodies, cell lines, animals and software tools, with enough information to allow them to be uniquely identified, should be included in the Methods section. Authors are strongly encouraged to cite Research Resource Identifiers (RRIDs) for antibodies, model organisms and tools, where possible.<br><br>Have you included the information requested as detailed in our Minimum Standards Reporting Checklist?	Yes
**Availability of data and materials**<br><br>All datasets and code on which the conclusions of the paper rely must be either included in your submission or deposited in publicly available repositories (where available and ethically appropriate), referencing such data using a unique identifier in the references and in the "Availability of Data and Materials" section of your manuscript.	Yes

Powered by Editorial Manager® and ProduXion Manager® from Aries Systems Corporation

Have you have met the above requirement as detailed in our Minimum Standards Reporting Checklist?	

Manuscript Click here to access/download;Manuscript;Opgenoorth_for_production.docx

The GenTree Platform: growth traits and tree-level environmental data in twelve European forest tree species

Opgenoorth, Lars[1,2], Dauphin, Benjamin[2], Benavides, Raquel[3], Heer, Katrin[1], Alizoti, Paraskevi[4], Martínez-Sancho, Elisabet[2], Alía, Ricardo[5], Ambrosio, Olivier[6], Audrey, Albet[7], Auñón, Francisco[5], Avanzi, Camilla[8], Avramidou, Evangelia[4], Bagnoli, Francesca[8], Barbas, Evangelos[4], Bastias, Cristina C.[9], Bastien, Catherine[10], Ballesteros, Eduardo[5], Beffa, Giorgia[2], Bernier, Frédéric[7], Bignalet, Henri[7], Bodineau, Guillaume[11], Bouic, Damien[7], Brodbeck, Sabine[2], Brunetto, William[6], Buchovska, Jurata[12], Buy, Melanie[6], Cabanillas-Saldaña, Ana M.[13], Carvalho, Bárbara[3], Cheval, Nicolas[7], Climent, José M.[5], Correard, Marianne[6], Cremer, Eva[14], Danusevičius, Darius[12], Del Caño, Fernando[5], Denou, Jean-Luc[7], di Gerardi, Nicolas[2], Dokhelar, Bernard[7], Ducousso, Alexis[15], Eskild Nilsen, Anne[16], Farsakoglou, Anna-Maria[4], Fonti, Patrick[2], Ganopoulos, Ioannis[17], García del Barrio, José M.[5], Gilg, Olivier[6], González-Martínez, Santiago C.[7], Graf, René[2], Gray, Alan[18], Grivet, Delphine[5], Gugerli, Felix[2], Hartleitner, Christoph[19], Hollenbach, Enja[1], Hurel, Agathe[7], Issehut, Bernard[7], Jean, Florence[6], Jorge, Veronique[20], Jouineau, Arnaud[6], Kappner, Jan-Philipp[1], Kärkkäinen, Katri[21], Kesälahti, Robert[22], Knutzen, Florian[14], Kujala, Sonja T[21], Kumpula, Timo A[22], Labriola, Mariaceleste[8], Lalanne, Celine[15], Lambertz, Johannes[1], Lascoux, Martin[23], Lejeune, Vincent[11], Le-Provost, Gregoire[15], Levillain, Joseph[24], Liesebach, Mirko[25], López-Quiroga, David[3], Meier, Benjamin[2], Malliarou, Ermioni[4], Marchon, Jérémy[2], Mariotte, Nicolas[6], Mas, Antonio[3], Matesanz, Silvia[26], Meischner, Helge[1], Michotey, Célia[27], Milesi, Pascal[28], Morganti, Sandro[2], Nievergelt, Daniel[2], Notivol, Eduardo[29], Ostreng, Geir[16], Pakull, Birte[25], Perry, Annika[18], Piotti, Andrea[8], Plomion, Christophe[15], Poinot, Nicolas[7], Pringarbe, Mehdi[6], Puzos, Luc[7], Pyhäjärvi, Tanja[22], Raffin, Annie[7], Ramírez-Valiente, José A.[5], Rellstab, Christian[2], Remi, Dourthe[7], Richter, Sebastian[1], Robledo-Arnuncio, Juan J.[5], San Segundo, Sergio[5], Savolainen,Outi[22], Schueler, Silvio[30], Schneck,Volker[31], Scotti, Ivan[6], Semerikov, Vladimir[32], Slámová, Lenka[2], Sønstebø, Jørn Henrik[16], Spanu, Ilaria[8], Thevenet, Jean[6], Tollefsrud, Mari Mette[16], Turion, Norbert[6], Vendramin, Giovanni Giuseppe[8], Villar, Marc[20], von Arx, Georg[2], Westin, Johan[33], Fady,Bruno[6], Myking, Tor[16], Valladares, Fernando[3], Aravanopoulos, Filippos A.[4], Cavers, Stephen[18]

[1]Philipps University Marburg, Faculty of Biology, Karl-von-Frisch Strasse 8, 35043 Marburg, Germany

[2]Swiss Federal Research Institute WSL, Zǘrcherstrasse 111, 8903, Birmensdorf, Switzerland

[3]LINCGlobal, Department of Biogeography and Global Change, Museo Nacional de Ciencias Naturales, CSIC, Serrano 115 dpdo, 28006, Madrid, Spain

[4]Aristotle University of Thessaloniki, School of Forestry and Natural Environment, Laboratory of Forest Genetics and Tree Improvement, 54124 Thessaloniki, Greece

[5]Instituto Nacional de Investigación y Tecnología Agraria y Alimentaria - Centro de Investigación Forestal (INIA-CIFOR), Ctra. de la Coruña km 7.5, 28040, Madrid, Spain

[6]Institut National de Recherche en Agriculture, Alimentation et Environment (INRAE), Domaine Saint Paul, Site Agroparc, 84914, Avignon, France

[7]Institut National de Recherche en Agriculture, Alimentation et Environment (INRAE), UEFP, 33610 Cestas, France

[8]Institute of Biosciences and BioResources, National Research Council (CNR), via Madonna del Piano 10, 50019, Sesto, Fiorentino, Italy

[9]Centre d'Ecologie Fonctionnelle et Evolutive (CEFE), CNRS, UMR 5175, Montpellier, France

[10]Institut National de Recherche en Agriculture, Alimentation et Environment (INRAE), Dept ECOFA, F-45075 Orléans, France

[11]Institut National de Recherche en Agriculture, Alimentation et Environment (INRAE), GBFOR, 45075 Orléans, France

[12]Vytautas Magnus University, Studentu Street 11, 53361, Akademija, Lithuania.

[13]Departamento de Agricultura, Ganadería y Medio Ambiente, Gobierno de Aragón, P. Mª Agustín 36, 50071 Zaragoza, Spain

[14]Bavarian Institute for Forest Genetics , Forstamtsplatz 1, 83317 Teisendorf, Germany
[15]INRAE, Univ. Bordeaux, BIOGECO, F-33770 Cestas, France
[16]Division of Forestry and Forest Resources, Norwegian Institute of Bioeconomy Research (NIBIO), P.O. Box 115, 1431, Ås, Norway
[17]Institute of Plant Breeding and Genetic Resources, Hellenic Agricultural Organization DEMETER (ex NAGREF), Thermi, 57001, Greece
[18]UK Centre for Ecology and Hydrology, Bush Estate Penicuik, EH26 0QB, United Kingdom
[19]LIECO GmbH & Co KG
[20]Institut National de Recherche en Agriculture, Alimentation et Environment (INRAE), ONF, BIOFORA, 45075 Orléans, France
[21]Natural Resources Institute Finland, Paavo Havaksentie 3, 90014, University of Oulu, Finland
[22]University of Oulu, Pentti Kaiteran katu 1, 90014, University of Oulu, Finland
[23]Department of Ecology & Genetics, EBC, Uppsala University, Norbyvägen 18D, 75236, Uppsala, Sweden
[24]Université de Lorraine, AgroParisTech, INRAE, SILVA, 54000 Nancy, France
[25]Thünen Institute of Forest Genetics, Sieker Landstr. 2, Grosshansdorf, 22927, Germany
[26]Área de Biodiversidad y Conservación, Universidad Rey Juan Carlos, Calle Tulipán s/n, 28933, Móstoles, Spain
[27]Institut National de Recherche en Agriculture, Alimentation et Environment (INRAE), URGI, Versailles, France
[28]Department of Ecology & Genetics, EBC, Science for Life Laboratory, Uppsala University, Uppsala, Sweden
[29]Centro de Investigación y Tecnología Agroalimentaria de Aragón - Unidad de Recursos Forestales (CITA),Avda. Montañana 930, 50059, Zaragoza, Spain
[30]Austrian Research Centre for Forests (BFW), Seckendorff-Gudent-Weg 8, 1131, Wien, Austria
[31]Thünen Institute of Forest Genetics, Eberswalder Chaussee 3a, Waldsieversdorf, 15377, Germany
[32]Institute of Plant and Animal Ecology, Ural branch of RAS, 8 Marta St. 202, 620144, Ekaterinburg, Russia
[33]Skogforsk, Tomterna 1, 918 21 Sävar, Sweden

opgenoorth@uni-marburg.de; lars.opgenoorth@wsl.ch
benjamin.dauphin@wsl.ch
rbenavidescalvo@gmail.com
katrin.heer@uni-marburg.de
alizotp@for.auth.gr
elisabet.martinez@wsl.ch
alia@inia.es
olivier.ambrosio@inrae.fr
audrey.albet.1@inrae.fr
aunon.francisco@inia.es
camilla.avanzi@ibbr.cnr.it
aevaggelia@yahoo.com
francesca.bagnoli@ibbr.cnr.it
vbarbas@for.auth.gr
crbasc@gmail.com
catherine.bastien@inrae.fr
eduardo.ballesteros@inia.es
giorgia.beffa@gmail.com
frederic.bernier@inrae.fr
henri.bignalet@inrae.fr
guillaume.bodineau@inrae.fr
damien.bouic@inrae.fr

103 sabine.brodbeck@wsl.ch
104 william.brunetto@inrae.fr
105 jurata.buchovska@vdu.lt
106 Melanie.Buy@inrae.fr
107 acabanillas@aragon.es
108 babic.barbosa@gmail.com
109 nicolas.cheval@inrae.fr
110 climent@inia.es
111 marianne.correard@inrae.fr
112 Eva.Cremer@awg.bayern.de
113 darius.danusevicius@vdu.lt
114 umgf@inia.es
115 jean-luc.denou@inrae.fr
116 nicolas.degirardi@gmail.com
117 bernard.dokhelar@inrae.fr
118 alexis.ducousso@inrae.fr
119 anne.eskild.nilsen@nibio.no
120 amfarsakoglou@gmail.com
121 patrick.fonti@wsl.ch
122 giannis.ganopoulos@gmail.com
123 jmgarcia@inia.es
124 olivier.gilg@inrae.fr
125 santiago.gonzalez-martinez@inrae.fr
126 rene.graf@wsl.ch
127 alangray@ceh.ac.uk
128 dgrivet@inia.es
129 felix.gugerli@wsl.ch
130 hartleitner@sfl.at
131 Hollenb4@students.uni-marburg.de
132 agathe.hurel@inrae.fr
133 bernard.issenhuth@inrae.fr
134 florence.jean@inrae.fr
135 veronique.jorge@inrae.fr
136 arnaud.jouineau@inrae.fr
137 kappnerj@students.uni-marburg.de
138 katri.karkkainen@luke.fi
139 robert.kesalahti@oulu.fi
140 FAKnutzen@gmail.com
141 sonja.kujala@luke.fi
142 timo.kumpula@oulu.fi
143 mclabriola@gmail.com
144 celine.lalanne@inrae.fr
145 Lambertj@students.uni-marburg.de
146 martin.lascoux@ebc.uu.se
147 vincent.lejeune@inrae.fr
148 gregoire.le-provost@inrae.fr
149 joseph.levillain@inrae.fr
150 mirko.liesebach@thuenen.de
151 david.quiroga@csic.es
152 meier.benjamin@outlook.com
153 ermionimalliarou@yahoo.gr
154 jeremy.marchon@romandie.ch

nicolas.mariotte@inrae.fr
antonio.mas@mncn.csic.es
silvia.matesanzgarcia@gmail.com
helgem@posteo.de
celia.michotey@inrae.fr
pascal.milesi@scilifelab.uu.se
msandro@posteo.ch
daniel.nievergelt@wsl.ch
enotivol@cita-aragon.es
geir.ostreng@nibio.no
birte.pakull@thuenen.de
annt@ceh.ac.uk
andrea.piotti@ibbr.cnr.it
christophe.plomion@inrae.fr
nicolas.poinot@inrae.fr
mehdi.pringarbe@inrae.fr
luc.puzos@inrae.fr
tanja.pyhajarvi@oulu.fi
annie.raffin@inrae.fr
josealberto.ramirezvaliente@gmail.com
christian.rellstab@wsl.ch
remi.dourthe@inrae.fr
richte3d@staff.uni-marburg.de
jjrobledo@gmail.com
sergio.sansegundo@inia.es
outi.savolainen@oulu.fi
silvio.schueler@bfw.gv.at
volker.schneck@thuenen.de
ivan.scotti@inrae.fr
semerikov@ipae.uran.ru
lenka.mateju@wsl.ch
jorn.sonstebo@nmbu.no
ilaria.spanu@ibbr.cnr.it
jean.thevenet@inrae.fr
Mari.Mette.Tollefsrud@nibio.no
Norbert.Turion@inrae.fr
giovanni.vendramin@ibbr.cnr.it
marc.villar@inrae.fr
georg.vonarx@wsl.ch
johan.westin@skogforsk.se
bruno.fady@inrae.fr
tor.myking@nibio.no
valladares@ccma.csic.es
aravanop@auth.gr
scav@ceh.ac.uk

Corresponding authors: opgenoorth@uni-marburg.de, benjamin.dauphin@wsl.ch, scav@ceh.ac.uk

Abstract

Background:

Progress in the field of evolutionary forest ecology has been hampered by the huge challenge of phenotyping trees across their ranges in their natural environments, and the limitation in high-resolution environmental information.

Findings:

The GenTree Platform contains phenotypic and environmental data from 4,959 trees from twelve ecologically and economically important European forest tree species: *Abies alba* Mill. (silver fir), *Betula pendula* Roth. (silver birch), *Fagus sylvatica* L. (European beech), *Picea abies* (L.) H. Karst (Norway spruce), *Pinus cembra* L. (Swiss stone pine), *Pinus halepensis* Mill. (Aleppo pine), *Pinus nigra* Arnold (European black pine), *Pinus pinaster* Aiton (maritime pine), *Pinus sylvestris* L. (Scots pine), *Populus nigra* L. (European black poplar), *Taxus baccata* L. (English yew), and *Quercus petraea* (Matt.) Liebl. (sessile oak). Phenotypic (height, diameter at breast height, crown-size, bark-thickness, biomass, straightness, forking, branch angle, fructification), regeneration, environmental in-situ measurements (soil depth, vegetation cover, competition indices), and environmental modeling data extracted by using bilinear interpolation accounting for surrounding conditions of each tree (precipitation, temperature, insolation, drought indices) were obtained from trees in 194 sites covering the species' geographic ranges and reflecting local environmental gradients.

Conclusion:

The GenTree Platform is a new resource for investigating ecological and evolutionary processes in forest trees. The coherent phenotyping and environmental characterization across 12 species in their European ranges allow for a wide range of analyses from forest ecologists, conservationists, and macro-ecologists. Also, the data here presented can be linked to the GenTree Dendroecological collection, the GenTree Leaf Trait collection, and the GenTree Genomic collection presented elsewhere, which together build the largest evolutionary forest ecology data collection available.

Measurements	vegetation cover • rock cover • soil depth • competition index • regeneration • diameter at breast height • height • crown size • bark thickness • number of fruits • stem straightness • branch angle • forking index
Technology Type(s)	bark gauges • calculations • caliper • clinometer • GPS device • increment corer • laser distance measurement • telescopic measuring pole
Factor Type(s)	tree species
Sample Characteristic Organism	*Abies alba* • *Betula pendula* • *Fagus sylvatica* • *Picea abies* • *Pinus cembra* • *Pinus halepensis* • *Pinus nigra* • *Pinus pinaster* • *Pinus sylvestris* • *Populus nigra* • *Taxus baccata* • *Quercus petraea*
Sample Characteristic Location	Europe

Machine-accessible metadata file describing the reported data:

Keywords

Regeneration, DBH, height, crown-size, bark-thickness, fruit-number, stem-straightness, branch-angle, forking-index, soil-depth

Context

The impacts of climate change and land-use change on forests are already severe, as observed, for example, following the extreme summer drought of 2018 that triggered a massive increase in mortality in Central European forests[1]. Furthermore, changes are expected to be acute in the future, altering distribution ranges and ecosystem functioning, as well as the interactions among species [2]. Forecasts indicate that near-surface temperature will shift poleward at mean rates of 80-430 m yr^{-1} for temperate forests during the 21st century [3]. This translates into northward shifts of trees' bioclimatic envelopes from 300 to 800 km within one century [3]. More importantly, the frequency and intensity of drought events, heat waves, forest fires, and pest outbreaks [4] are expected to increase.

In the light of these changes, species and forest ecosystem resilience will depend on the extent and structure of phenotypic plasticity, genetic variation, and adaptive potential, as well

as dispersal ability. From the results of extensive networks of field experiments (provenance trials), it has long been shown that tree species are locally adapted at multiple spatial scales. In Europe, where most tree populations have established following post-glacial recolonization, such patterns of local adaptation must have developed rapidly and despite long generation time and extensive gene flow [5], a process enabled by high levels of within-population plasticity, genetic and epigenetic variation, and large population sizes [6]. Recent work has shown that genetic variation for stress response may be strongly structured along environmental gradients, such as water availability [7], temperature [8], or photoperiod [9]. However, the spatial patterns of current adaptation in particular phenotypic traits are only partly informative regarding the potential for future adaptation under a changing climate. To advance our understanding of the adaptive potential of trees, it is crucial to evaluate multiple traits in parallel to be able to model their putative response to new environmental conditions.

Recently, substantial effort has been made to identify specific genes and gene combinations that have undergone selection, by associating mutations at candidate loci with phenotypes related to stress events [10,11] or with environmental variables [12]. This latter example by Yeaman and co-workers [12] is one of the first association studies in forest tree species on a large genomic scale and the first to investigate convergent local adaptation in distantly related tree species. However, progress in this field has been hampered by limited genomic resources, the lack of small-scale, individual tree-level environmental information [13], and the huge challenge of phenotyping trees in their natural environments [14,15].

The GenTree Platform aims to address these challenges by providing individual level, high-resolution phenotypic and environmental data for a set of up to 20 sampling sites for each of twelve ecologically and economically important forest tree species across Europe. For a subset of seven species (*B. pendula*, *F. sylvatica*, *P. abies, P. pinaster*, *P. sylvestris*, *Populus nigra*, and *Q.petraea*), the sampling of sites was carried out in pairs, i.e. contained

two stands that were close enough to be connected by gene flow but situated in contrasting environments.

The sampling design described here was used for collecting phenotypic traits and ecological data. Also, tree ring and wood density measurements for the same trees were assessed [16], and datasets on leaf traits, including specific leaf area (SLA) and isotopic content [17], and high-density single nucleotide polymorphism (SNP) data for each tree, were established, that will be published in GeneBank. All data and metadata information are gathered in the GnpIS repository (https://urgi.versailles.inra.fr/ephesis/ephesis/viewer.do#dataResults/trialSetIds=27) which makes updates possible[18].

We investigated the extent to which other datasets comparable to the data presented here exist by screening our twelve species in the TRY Plant Trait Data Base, the ITDRB, and the biomass and allometry database for woody plants (BAAD). While this is a systematic approach, it leaves out a large number of tree species and therefore we cannot claim to have a comprehensive overview of the existing data. However, all three databases are large collections that include at least some of the tree measurements we present. Even though these are tremendous resources, the major difference is that based on their nature as collecting points of numerous independent datasets, there is no coherent sampling scheme in these collections as such, meaning that the number of trees per site, the way of tree selection, measured phenotypes, and provided environmental information vary greatly and therefore do not allow for coherent comparative analyses such as those of the GenTree Platform. For example, BAAD reports DBH data for only four of the species presented here, namely *Betula pendula* with three populations, *Fagus sylvatica* with two populations, *Picea abies* with four populations, and *Pinus sylvestris* with ten populations. In the larger TRY database, all of our species are represented, but the variability of sampling schemes is much more heterogeneous concerning traits, number of populations per species, and metadata.

For example, DBH measurements are being reported 232 times from a total of 12 *Betula pendula* populations. Of these, the vast majority of the 170 measurements are from one population while from many other populations only one or up to five measurements are reported. Also, the measurements stem from five different original studies and thus having very different levels of additional information. We conclude that the core value of our reported data lies in the coherent sampling design and yet the large number of sampled populations and individuals per species.

Methods

All recorded parameters are listed in Table 1.

Sampling strategy

To optimize the sampling design for genome scans and association studies, we followed the recent theoretical work by Lotterhos and Whitlock [19,20], which indicates that a paired sampling design has more power to detect the genomic signatures of local adaptation. Using this framework, populations from across the natural range of a species are sampled in pairs, with the two sites in each pair situated geographically close enough to be genetically similar at neutral genes due to a common evolutionary history and ongoing gene flow, but in distinct selective niches such that the local fitness optimum differs between the two sites. This sampling confers more power to detect evidence of selection in the genome through either association with environmental or phenotypic variables or the detection of outliers (e.g. for genetic differentiation, F_{ST}) (ibid.). Trees are very amenable to a pairwise approach since they are known to be locally adapted, often at fine spatial scales [21,22] and irrespective of gene flow distances [6]. This strategy was followed for a subset of seven species (see above) for which genomic resources were available (i.e. full or draft genome).

Such local niche contrasts are neither easy to identify nor readily available when environments are very homogenous. Therefore, a second principle of the sampling design

was to cover a large part of each species' natural geographic range (Fig. 1) and environmental space (Fig. 2) to capture selective niche variation. Finally, sites with a history of intensive management or any other intense and obvious anthropogenic or natural disturbances were avoided. This strategy was followed for all the 12 species.

Selection of trees on sites

A minimum of 25 trees was sampled per site to capture the natural phenotypic and genetic variability. Trees had to be mature but not senescent, dominant or codominant and had to show no signs of significant damage due to pests and diseases or generally low vigor. Sampled trees were at least 30 meters apart and, where possible, were chosen along several parallel linear transects across each site, typically resulting in 2-4 transects per sampling site to keep the overall sampling area below 3 hectares.

Site and tree metadata

Sites were labeled by a two-letter country code (ISO 3166-1 alpha-2) followed by a two-letter species code and a two-digit site number (Table 1). Individual tree-labels added another two-digit tree number. Every tree was permanently labeled so that future studies can resample subsets or the entire GenTree collection for gaining time-series data of individual traits or to add new phenotypes to the analyses. Be aware, that permission of the respective landowners must be obtained before sampling. Handheld GPS devices were used to record the position of each tree. The precision of GPS measurements in forests is notoriously challenging: regular commercial devices achieve an accuracy of about 8-15 m with good satellite coverage. Given that trees were selected with a minimum distance of 30 m this accuracy was sufficient for the correct positioning of trees relative to each other. An overall population position was defined by taking the mean value across all the individual tree measurements. Coordinates were in decimal degrees with 4 decimal units to reflect the general measurement accuracy (~11.1 m) and were stored in the WGS 84 reference system. GPS devices were also used to record the tree's elevation, either directly or through

post-hoc positioning in digital elevation models. The local aspect at the site of the tree was measured by a compass in five-degree steps in the direction of the steepest slope.

The Metadata for each site consists of an ID code (see above), sampling date, location (GPS coordinates, see above), and elevation in meters above sea level (m a.s.l). Each stand was also characterized as being monospecific or mixed (in the latter case the most common co-occurring species was noted), stand structure was noted as single or multiple layered, and the age distribution as even or uneven (categorical variables).

Competition index at tree level

Competition indices were calculated following Canham et al. [23] and Lorimer et al. [24]. Specifically, the first index following Lorimer [24] was calculated as $NCI = \sum_{i=1}^{5}({}^{DBH_j}/_{DBH_i})/dist_i$ that follows the same notation as above, and where *DBH* is the *DBH* of the subject trees *j* and *i*.

Second, the distance-dependent competition index (NCI) following Canham et al. [23] was computed as $NCI = \sum_{i=1}^{5}({}^{DBH_i}/_{dist_i})$ where DBH_i is the diameter at breast height of competitor tree *i*, and $dist_i$ is the distance between the subject tree and competitor tree *i*. This index assumes that the net effects of neighboring trees vary as a direct function of the size of the neighbors and as an inverse function of the distance. For this purpose, the distance to the five nearest neighbors of each target tree was measured and their respective diameter at breast height was measured.

Moreover, it was noted whether competitor trees were conspecific to the target tree or not. Each multi-stemmed tree was considered as a single competitor where each stem larger than 15 cm DBH was measured and added to the sum of means.

Environmental characteristics within subplots around each tree

Surrounding each target tree, slope, vegetation cover (without tree cover), and stone content

were assessed in a 10 m x 10 m plot. The slope was assessed using a clinometer. Vegetation and rock cover were estimated in the classes <5%, 5-20%, 20-40%, 40-60%, 60-80%, 80-95%. Soil depth was estimated at three random points in the quadrat to a maximum of 60 cm with a pike and was averaged across these three values.

Regeneration

In the same 10 m x 10 m plots, natural regeneration of the target species was assessed according to the following four classes: absent (no recruit visible), scattered (few/scattered individuals), grouped (presence of scattered groups within the plot), and abundant (regularly spread all over the plot) and is indicated in the database with values from 1-4. As this method cannot resolve maternity, the results indicate realized fecundity at the stand level.

Growth traits

DBH (cm)

DBH was measured at a stem height of 1.3 m using either a caliper by measuring two perpendicular diameters and subsequently taking the average of these two measurements or by measuring the circumference of the tree using a tape and computing the diameter from that value. Each measurement was performed to the nearest 0.1 cm. If a tree had more than one trunk, all of them were measured and the average was recorded.

Height (m)

Height from the ground to the top of the crown was measured using a hypsometer (Nikon forestry Pro Laser), a laser vertex (Haglof Vertex III, Langsele, Sweden), or a Laser Range Meter (Bosch GLM 50 C, Leinfelden-Echterdingen, Germany). For short trees, a telescopic measuring pole was used. Height was noted to the nearest 0.1 m. To forego errors introduced by measuring height on sloping ground, height measurements on slopes were conducted from the same elevation as the tree’s base by approaching the tree sideways. Where this was not possible, a slope correction factor was used.

Crown size (m²)

The crown size was measured as the circular and ellipsoid plane area of the crown. For this, we measured two perpendicular crown diameters (canopy 1 and 2) by using a measurement tape, with the first measurement being made along the longest axis of the crown, from one edge to the other, and by visually projecting the crown margin onto the ground to the nearest decimeter. For the ellipse area, we calculated $\left({}^{d_i}/_2\right) * \left({}^{d_j}/_2\right) * \pi$ and for the circular area $(d_i+d_j)/2*\pi$.

Bark thickness (mm)

For measuring bark thickness, we used bark gauges (Haglof Barktax) or a tape after extracting the bark with a small caliper (if bark could be detached without tree damage) or increment borers (Haglof increment borer) in case of strong and thick bark. Five measurements were taken for each tree at breast height and the average was calculated. For tree species with a clear dichotomy of bark thickness (e.g. old *Populus nigra*, *T. baccata*), we included at least two measurements from the thinner and thicker bark areas each.

Number of fruits (units)

In conifers, cones were counted by providing the average of three rounds of counting, made by an observer on the ground using binoculars. Only mature (brown) and closed cones were counted, i.e., those containing seeds, and not immature (green) or open cones, whose seeds had already been dispersed (open cones often stay on the branch for several years after seeds are dispersed). In broadleaves, the number of fruits was counted for 30 seconds, repeating the procedure three times to then average the three counts.

In the case of species with very small fruits that are hard to see individually and in locations with a very limited view of the canopy, each tree was assigned to one of five categories, namely 0 (no fruits), 1 (a few fruits in a small section of the crown), 2 (a few fruits in two or

more sections of the crown), 3 (a lot of fruits in a small section of the crown), and 4 (a lot of fruits in two or more sections of the crown).

Straightness

Straightness of the stem was classified according to five levels: (1) No straight stem, (2) moderate or strong bends, (3) slight to moderate bend in different directions, (4) fairly straight (in one direction slightly crooked), (5) absolutely straight. This was performed on the lower 15 m of the tree beginning from the ground with the crown not taken into account. In the case of forked stems, only the trunk below the deepest forking point was evaluated.

Branch angle

Branch angle was classified at two successive whorls according to a five-scale scheme in conifers with (1) <23°, (2) 23-45°, (3) 45-67°, (4) 67°-90°, (5) > 90°, and a four-scale scheme in broadleaves omitting the >90° class. In black poplar, only the top two meters of the crown were considered.

Forking index

The branching of a tree in two (fork) or more (ramiform) equally thick and long stems was assessed with a forking index. The index took into account two parameters. First a score for the relative position of the fork: (4) no forking, (3) forking in the upper third of the tree, (2) forking in the middle third of the tree, (1) forking in the lower third of the tree; and second the number of axes (stems). The score of the relative position was then multiplied by ten and divided by the number of axes.

Modeled environmental data extracted for GenTree sites

Topography, soil, and climate data were compiled to characterize environmental conditions in each GenTree sampling site as follows.

Topography

We used the European digital elevation model to describe topographic conditions at 25 m spatial resolution with a vertical accuracy of about ± 7 meters (EU-DEM v. 1.1 from the Copernicus program; https://land.copernicus.eu/). We derived 14 variables (Table 2) based on biological hypotheses and their informative power at the local scale [25]. We calculated morphometric, hydrologic, and radiation grids for each GenTree site and visually inspected data integrity using SAGA 6.2 [26] (details in Table 2).

Soil

We collected available data on water capacity at seven soil depths using SoilGrids250m [27]. We estimated Pearson's correlation coefficients, *r*, between soil layers and then averaged the four first superficial (0, 5, 15, and 30 cm) and the three deeper (60, 100, and 200 cm) layers that were highly correlated, respectively.

Climate

We extracted climate data with a high spatial resolution (30 arcsec) using CHELSA v. 1.2 [28]. CHELSA is based on a quasi-mechanistic statistical downscaling global reanalysis and global circulation model that, in particular, reliably interpolates the amount of precipitation using an orographic rainfall and wind effect. The dataset consisted of 48 climatic, 19 bioclimatic, four drought- and two frost-related variables for the reference period 1979-2013 (Table 2; http://chelsa-climate.org/bioclim/). We extracted all modeled environmental values for each individually geo-referenced tree using the *extract* function of the R package RASTER (Hijmans and van Etten 2016[29]). The surrounding conditions (i.e. adjacent pixels) of each tree were incorporated by the bilinear interpolation method when extracting the data.

Table 1 Variables names, explanations, and specifications measured for all 4,959 trees and all 194 GenTree sites.

Variable name	Variable explanation	Specification
**GenTree Platform metadata**		

m01.spec	Species abbreviations	*Abies Alba* [AA], *Betula pendula* [BP], *Fagus sylvatica* [FS], *Picea abies* [PA], *Pinus cembra* [PC], *Pinus halepensis* [PH], *Pinus nigra* [PN], *Populus nigra* [PO], *Pinus pinaster* [PP], *Pinus sylvestris* [PS], *Quercus petraea* [QP], *Taxus baccata* [TB]
m02.country	Country abbreviations	Isocode 6133-2; Austria [AT], Switzerland [CH], Germany [DE], Spain [ES], Finland [FI], France [FR], Great Britain [GB], Greece [GR], Italy [IT], Lithuania [LT], Norway [NO], Sweden [SE]
m03.site.num	Site numbers	Running numbers of sites per species 01-24
m04.site.id	Complete site-ID per species	Merger of m01-m03
m05.tree.num	Tree numbers	Running numbers within sites 01-25
m06.tree.id	Complete tree ID	Merger of m01-m03, m05
m07.trial.name	Site name	
m08.lat	Latitude	Decimal degrees, WGS84
m09.lon	Longitude	Decimal degrees, WGS84
**GenTree Platform phenotypes**		
p01.height	Height	Tree height, m
p02.dbh	DBH	Diameter at breast height, cm
p03.bark	Bark thickness mean	Mean value of bark thickness, cm
p04.trunk	Trunk straightness/flexuosity	5: absolutely straight, 4: fairly straight (in one direction slightly crooked), 3: slight to moderate bend in different directions, 2: moderate or strong bends, 1: no straight stem
p05.branch	Branch angle	1: <23° (steep), 2: 23° - 45°, 3: 45º-67º, 4: 67º-90º (plain), 5: >90º
p06.fork	Forking index	1: fork at the lower third of tree height, 2: fork at middle third, 3: fork at upper third, 4: no fork – multiplied by 10 and then divided by the number of stems
p07.canopy.1	Canopy projection REP 1	Crown diameter projection, m
p08.canopy.2	Canopy projection REP 2	Crown diameter projection, m
p09.crown.ellipse	Crown ellipse	Area of an ellipse (d1/2)*(d2/2)*π, m^2
p10.crown.round	Crown size	As some only have one diameter, round areas with the mean diameter $((D1+D2/2)/2)^2*\pi$, m^2
p11.regeneration	Natural regeneration	1: absent, 2: scattered, 3: groups, 4: abundant
p12.fruit.mean	Fruit/cone number	Number of fruits.
p13.basal.area		
**GenTree Platform in-situ environmental measurements**		
e01.plant.cover	Total plant cover	1: none, 2: little (5-20%), 3: low (20-40%), 4: medium (40-60%), 5: high (60-80%), 6: very high (80-95%), 7: full cover (>95%)
e02.comp.index.a	Competition index A	CI assessed following Canham et al 2004, and multi-stems as the sum
e03.comp.index.b	Competition index B	CI assessed following Canham et al 2004, and multi-stems assessing the sum of basal areas and then the DBH
e04.comp.index.c	Competition index C	CI assessed following Lorimer 1983, and multi-stems as the sum
e05.comp.index.d	Competition index D	CI assessed following Lorimer 1983, multi-stems assessing the sum of basal areas and then the DBH
e06.status		Dominant, co-dominant
e07.elevation	Elevation of the tree	Meters above sea level
e08.slope	Slope at the tree level	Slope in degrees
e09.aspect	Aspect at the tree level	0-360°
e10.soil.depth	Mean soil depth	Mean of 3 measures (measurement to a max. depth of 60 cm
e11.stone.content	Mean stone content	Mean of 3 measures (1: none, 2: little (5-20%), 3: low (20-40%), 4: medium (40-60%), 5: high (60-80%), 6: very high (80-95%), 7: full cover (>95%)
e12.rock.cover	Total rock cover	1: none, 2: little (5-20%), 3: low (20-40%), 4: medium (40-60%), 5: high (60-80%), 6: very high (80-95%), 7: full cover (>95%)

Table 2 Environmental variable names, explanations, and specifications modeled for all 4959 trees and 194 GenTree sites.

Variable name	Variable explanation	Specification
**GenTree Platform modeled environmental parameters**		
sample	Sample identification	unit: none; resolution [m]: none
country	Country code	unit: none; resolution [m]: none
countryspecies	Country and species code	unit: none; resolution [m]: none
species	Species code	unit: none; resolution [m]: none
population	Population identification	unit: none; resolution [m]: none
latwgs84	Latitude in WGS84	unit: degree; resolution [m]: 25
lonwgs84	Longitude in WGS84	unit: degree; resolution [m]: 25
latetrs89	Latitude in ETRS89	unit: degree; resolution [m]: 25
lonetrs89	Longitude in ETRS89	unit: degree; resolution [m]: 25
t01alt	Altitude	unit: m; resolution [m]: 25
t02slp	Slope	unit: degree; resolution [m]: 25
t03asp	Eastness	unit: degree; resolution [m]: 25
t04vcu	Profile curvature	unit: degree/m; resolution [m]: 25
t05hcu	Horizontal curvature	unit: degree/m; resolution [m]: 25
t06ddg	Downslope distance gradient	unit: degree; resolution [m]: 25
t07mpi	Morphometric protection index	unit: none; resolution [m]: 25
t08tpi	Topographic position index	unit: none; resolution [m]: 25
t09vrm	Vector ruggedness measure	unit: none; resolution [m]: 25
t10twi	Topographic wetness index	unit: none; resolution [m]: 25
t11svf	Sky-view factor	unit: none; resolution [m]: 25
t12sdir	Potential direct solar radiation	unit: kJ/m^2; resolution [m]: 25
t13sdif	Potential diffuse solar radiation	unit: kJ/m^2; resolution [m]: 25
t14stot	Potential total solar radiation	unit: kJ/m^2; resolution [m]: 25
awc15	Available water capacity (0-30cm)	unit: %; resolution [m]: 250
awc140	Available water capacity (60-200cm)	unit: %; resolution [m]: 250
bio01	Yearly mean temperature	unit: °C/10; resolution [m]: 1000
bio02	Mean diurnal range	unit: °C/10; resolution [m]: 1000
bio03	Isothermality	unit: °C/10; resolution [m]: 1000
bio04	Temperature seasonality	unit: °C/10; resolution [m]: 1000
bio05	Max temperature of warmest month	unit: °C/10; resolution [m]: 1000
bio06	Min temperature of coldest month	unit: °C/10; resolution [m]: 1000
bio07	Temperature annual range	unit: °C/10; resolution [m]: 1000
bio08	Mean temperature of wettest quarter	unit: °C/10; resolution [m]: 1000
bio09	Mean temperature of driest quarter	unit: °C/10; resolution [m]: 1000
bio10	Mean temperature of warmest quarter	unit: °C/10; resolution [m]: 1000
bio11	Mean temperature of coldest quarter	unit: °C/10; resolution [m]: 1000
bio12	Yearly precipitation sum	unit: kg m^{-2}; resolution [m]: 1000
bio13	Precipitation of wettest month	unit: kg m^{-2}; resolution [m]: 1000
bio14	Precipitation of driest month	unit: kg m^{-2}; resolution [m]: 1000
bio15	Precipitation seasonality	unit: kg m^{-2}; resolution [m]: 1000
bio16	Precipitation of wettest quarter	unit: kg m^{-2}; resolution [m]: 1000
bio17	Precipitation of driest quarter	unit: kg m^{-2}; resolution [m]: 1000
bio18	Precipitation of warmest quarter	unit: kg m^{-2}; resolution [m]: 1000
bio19	Precipitation of coldest quarter	unit: kg m^{-2}; resolution [m]: 1000
gdd	Growing degree days	unit: °C; resolution [m]: 1000
gsp	Accumulated precipitation	unit: kg m^{-2}; resolution [m]: 1000
shc	Hydrothermic coefficient	unit: (kg m^{-2}/10)/°C; resolution [m]: 1000
rh410	Relative humidity	unit: %; resolution [m]: 1000
fcf	Frost change frequency	unit: number of events; resolution [m]: 1000
nfd	Number of frost days	unit: number of days; resolution [m]: 1000
prec01	Precipitation sum in January	unit: kg m^{-2}; resolution [m]: 1000
prec02	Precipitation sum in February	unit: kg m^{-2}; resolution [m]: 1000
prec03	Precipitation sum in March	unit: kg m^{-2}; resolution [m]: 1000
prec04	Precipitation sum in April	unit: kg m^{-2}; resolution [m]: 1000
prec05	Precipitation sum in May	unit: kg m^{-2}; resolution [m]: 1000
prec06	Precipitation sum in June	unit: kg m^{-2}; resolution [m]: 1000
prec07	Precipitation sum in July	unit: kg m^{-2}; resolution [m]: 1000
prec08	Precipitation sum in August	unit: kg m^{-2}; resolution [m]: 1000
prec09	Precipitation sum in September	unit: kg m^{-2}; resolution [m]: 1000
prec10	Precipitation sum in October	unit: kg m^{-2}; resolution [m]: 1000
prec11	Precipitation sum in November	unit: kg m^{-2}; resolution [m]: 1000
prec12	Precipitation sum in December	unit: kg m^{-2}; resolution [m]: 1000
tmean01	Mean temperature in January	unit: °C/10; resolution [m]: 1000

tmean02	Mean temperature in February	unit: °C/10; resolution [m]: 1000
tmean03	Mean temperature in March	unit: °C/10; resolution [m]: 1000
tmean04	Mean temperature in April	unit: °C/10; resolution [m]: 1000
tmean05	Mean temperature in May	unit: °C/10; resolution [m]: 1000
tmean06	Mean temperature in June	unit: °C/10; resolution [m]: 1000
tmean07	Mean temperature in July	unit: °C/10; resolution [m]: 1000
tmean08	Mean temperature in August	unit: °C/10; resolution [m]: 1000
tmean09	Mean temperature in September	unit: °C/10; resolution [m]: 1000
tmean10	Mean temperature in October	unit: °C/10; resolution [m]: 1000
tmean11	Mean temperature in November	unit: °C/10; resolution [m]: 1000
tmean12	Mean temperature in December	unit: °C/10; resolution [m]: 1000
tmin01	Minimum temperature in January	unit: °C/10; resolution [m]: 1000
tmin02	Minimum temperature in February	unit: °C/10; resolution [m]: 1000
tmin03	Minimum temperature in March	unit: °C/10; resolution [m]: 1000
tmin04	Minimum temperature in April	unit: °C/10; resolution [m]: 1000
tmin05	Minimum temperature in May	unit: °C/10; resolution [m]: 1000
tmin06	Minimum temperature in June	unit: °C/10; resolution [m]: 1000
tmin07	Minimum temperature in July	unit: °C/10; resolution [m]: 1000
tmin08	Minimum temperature in August	unit: °C/10; resolution [m]: 1000
tmin09	Minimum temperature in September	unit: °C/10; resolution [m]: 1000
tmin10	Minimum temperature in October	unit: °C/10; resolution [m]: 1000
tmin11	Minimum temperature in November	unit: °C/10; resolution [m]: 1000
tmin12	Minimum temperature in December	unit: °C/10; resolution [m]: 1000
tmax01	Maximum temperature in January	unit: °C/10; resolution [m]: 1000
tmax02	Maximum temperature in February	unit: °C/10; resolution [m]: 1000
tmax03	Maximum temperature in March	unit: °C/10; resolution [m]: 1000
tmax04	Maximum temperature in April	unit: °C/10; resolution [m]: 1000
tmax05	Maximum temperature in May	unit: °C/10; resolution [m]: 1000
tmax06	Maximum temperature in June	unit: °C/10; resolution [m]: 1000
tmax07	Maximum temperature in July	unit: °C/10; resolution [m]: 1000
tmax08	Maximum temperature in August	unit: °C/10; resolution [m]: 1000
tmax09	Maximum temperature in September	unit: °C/10; resolution [m]: 1000
tmax10	Maximum temperature in October	unit: °C/10; resolution [m]: 1000
tmax11	Maximum temperature in November	unit: °C/10; resolution [m]: 1000
tmax12	Maximum temperature in December	unit: °C/10; resolution [m]: 1000

The local environmental contrasts varied among species and population pairs, most of which exhibited variability concerning elevation, temperature, precipitation, and water availability. Other local contrasts were based on radiation, soil water capacity, and topographic wetness index (among others). One special case is *Populus nigra*, a heliophilous pioneer species found naturally in riverine areas. Given this specific habitat, local contrasts were largely bound to the distance of the individual trees from the riverbed and thus for example to groundwater access or exposure to variation in the intensity and frequency of floods.

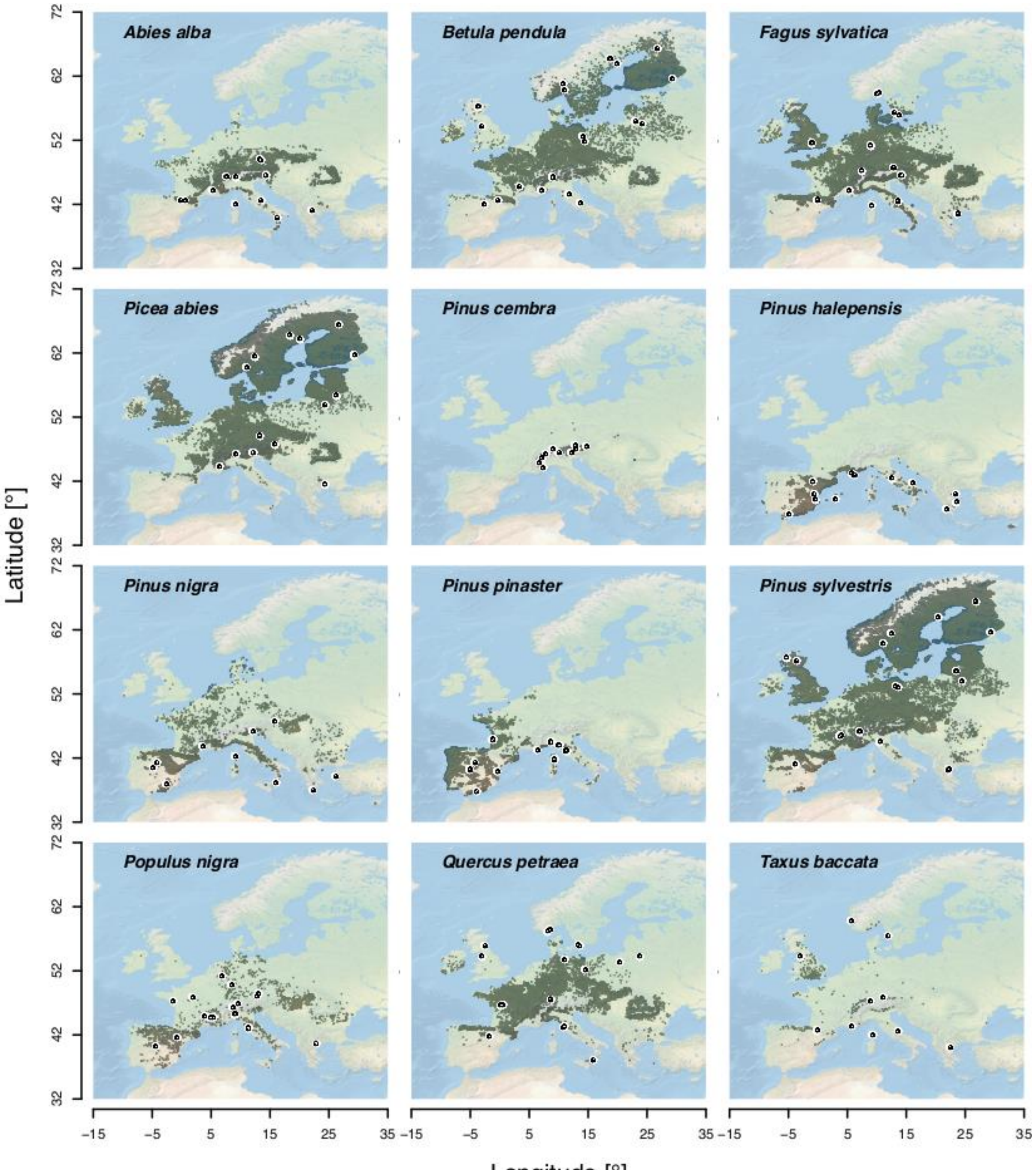

Fig. 1 Sampling sites (black dots) and distributions of the twelve selected tree species (dark-green shading) for *in-situ* phenotype measurements. Distribution maps are based on a comprehensive high-resolution tree occurrence dataset from the European Union [30].

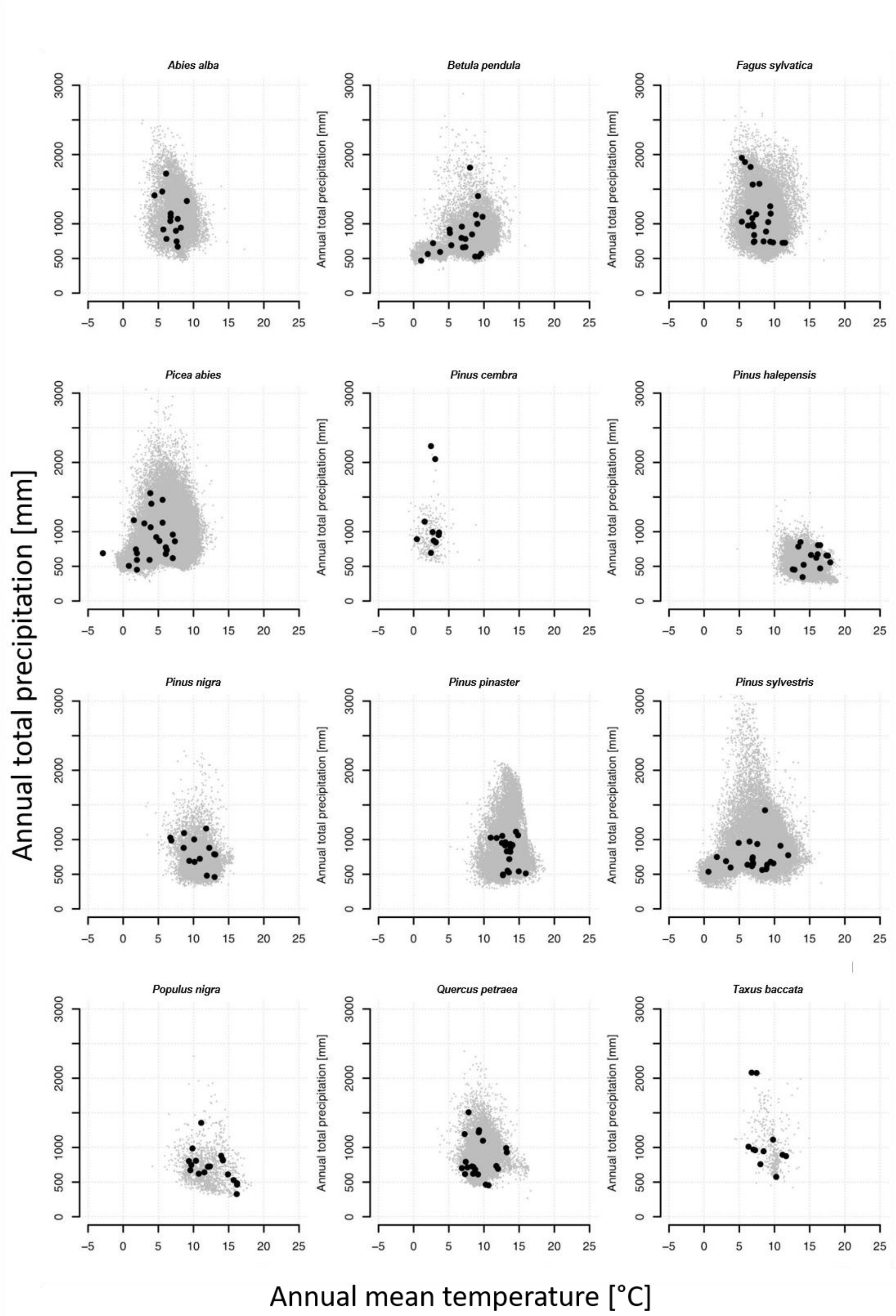

Fig. 2 Climate-space diagrams for the 12 selected European tree species with annual mean temperature on the x-axis and annual total precipitation on the y-axis. Grey points represent species occurrences based on a comprehensive high-resolution tree occurrence dataset for Europe [30] and black dots indicate the GenTree sites.

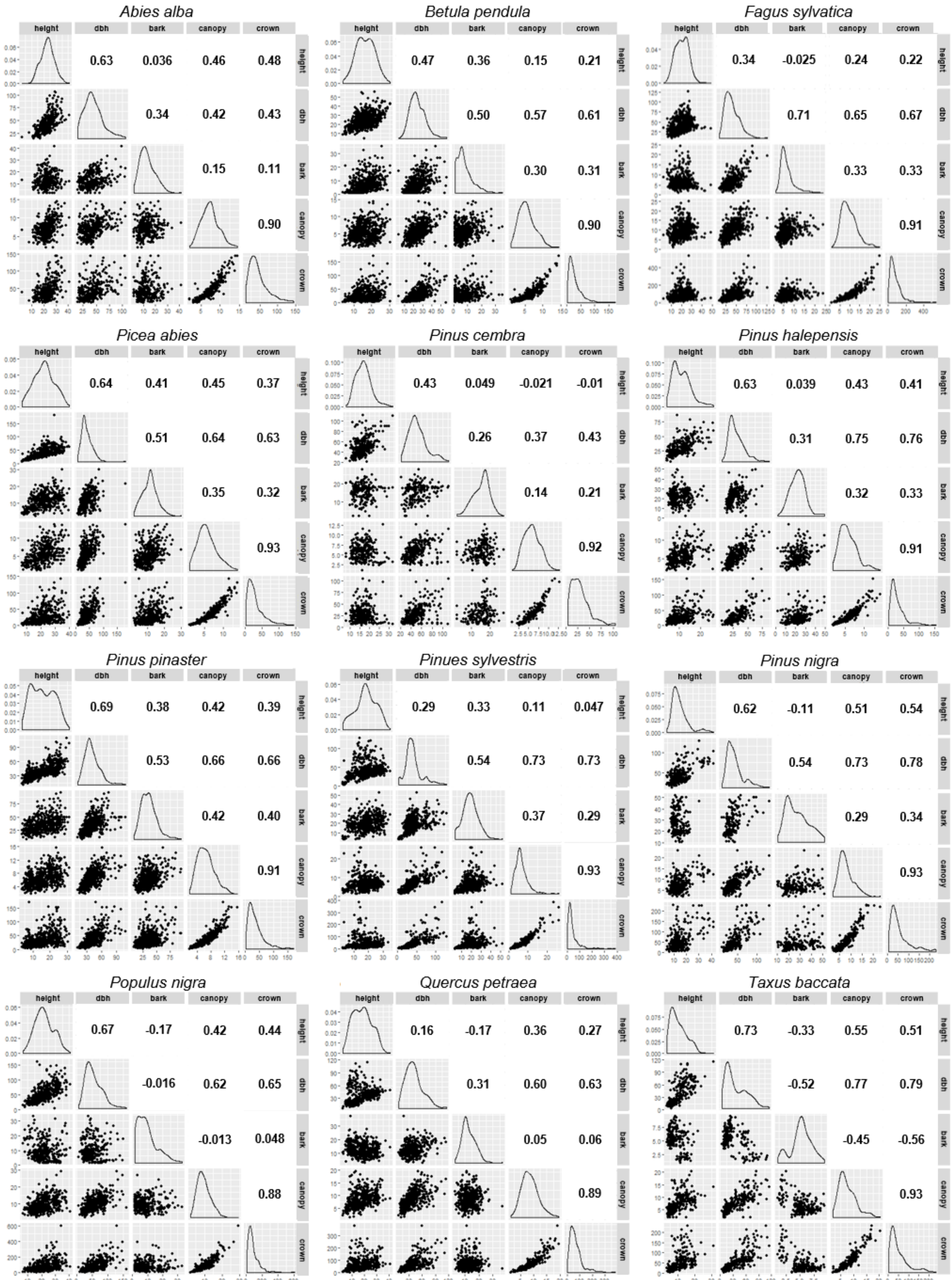

Fig. 3 Scatterplots, distributions, and Pearson's correlation coefficients, *r*, of GenTree phenotype measurements in the 12 selected European tree species.

Data validation and quality control

The database has been checked for consistency at different stages by various researchers between 2018-2020. Raw data were submitted by all partners to the GnpIS multispecies integrative information system (https://urgi.versailles.inra.fr/Tools/GnpIS) using preformatted Microsoft Excel templates. Data files were harmonized, merged, and subsequently verified following several steps:

1. Missing data and dubious entries were checked manually by examining the original data files obtained from the partners and by cross-checking cases with field books.
2. Descriptive statistics were calculated and plotted for all variables including minima, maxima, means, and variances. Outliers were checked against original data records and corrected when necessary.
3. Covariables were plotted determining whether relationships were reasonable and following the most complete set of similar relationships (Fig. 3).

Data Records

The data presented are structured in four independent csv files (GenTree_modelled_environmental_data.csv, GenTree_modelled_environmental_data_metadata.csv, GenTree_phenotypes_and_insitu_environmental_data.csv, and GenTree_phenotypes_and_insitu_environmental_data_metadata.csv) that can be merged using the site identifier (m04.site.id) or tree identifier (m06.tree.id). The same codes can be used to merge additional data namely from the GenTree Dendroecological Collection [16], the GenTree Leaf Trait Collection[17], and the GenTree Genomic Collection (personal communication). The first file contains the modeled environmental data, the second its metadata, the third the individual phenotypic traits and the insitu environmental data, and the fourth the metadata of the latter.

Availability of supporting data

The data files are available at the GigaDB repository [31] under a CC0 license. Excel versions of the data are available from figshare [32]. All the data are indexed in Table 2.

Acknowledgements¶

We would like to thank Juri Nievergelt, Anne Verstege, Marina Fonti, Kevin Kleeb, Frederick Reinig, Enrica Zalloni, Claudio de Sassi, Giacomo Poli for their field assistance. This work was performed using GnpIS, one of the facilities of the URGI platform (https://urgi.versailles.inra.fr/). We are also grateful to all the forest owners and national administrations for providing sampling permissions.

Author contributions¶

LO, RB, KH, BF, TM, FV, FAA, SC coordinated sampling design.

All authors contributed to the sampling design

LO, RB, KH, BF, TM, FV, FAA, SC coordinated field sampling.¶

All authors contributed to the field sampling.

CM, MB compiled and assembled in-situ measurements in the GnpIS database.¶

BD extracted climatic and topographic data and derived environmental indices for all the sampling sites.¶

RB, LO, BD, PAA, CM curated data, checked quality, and prepared the data sets with metadata descriptions for sharing and potential reuse.¶

LO, KH, BD, SC, BF wrote the manuscript.¶

BF coordinated GenTree

All authors commented on an earlier version and approved the current version of the manuscript.

Competing interests¶

The authors declare no competing interest.

Funding

This publication is part of the GenTree project which was funded by the European Union's Horizon 2020 research and innovation program under grant agreement No. 676876 (GenTree). This work was also supported by the Swiss Secretariat for Education, Research and Innovation (SERI) under contract No. 6.0032.

References

1. Schuldt, B. *et al.* A first assessment of the impact of the extreme 2018 summer drought on Central European forests. *Basic and Applied Ecology* **45,** 86–103; 10.1016/j.baae.2020.04.003 (2020).
2. Sitch, S. *et al.* Evaluation of the terrestrial carbon cycle, future plant geography and climate-carbon cycle feedbacks using five Dynamic Global Vegetation Models (DGVMs). *Global Change Biology* **14,** 2015–2039; 10.1111/j.1365-2486.2008.01626.x (2008).
3. Loarie, S. R. *et al.* The velocity of climate change. *Nature* **462,** 1052–1055; 10.1038/nature08649 (2009).
4. Pachauri, R. K. & Mayer, L. (eds.). *Climate change 2014. Synthesis report* (Intergovernmental Panel on Climate Change, Geneva, Switzerland, 2015).
5. Savolainen, O., Pyhäjärvi, T. & Knürr, T. Gene Flow and Local Adaptation in Trees. *Annu. Rev. Ecol. Evol. Syst.* **38,** 595–619; 10.1146/annurev.ecolsys.38.091206.095646 (2007).
6. Kremer, A. *et al.* Long-distance gene flow and adaptation of forest trees to rapid climate change. *Ecology Letters* **15,** 378–392; 10.1111/j.1461-0248.2012.01746.x (2012).
7. Eveno, E. *et al.* Contrasting patterns of selection at Pinus pinaster Ait. Drought stress candidate genes as revealed by genetic differentiation analyses. *Molecular Biology and Evolution* **25,** 417–437; 10.1093/molbev/msm272 (2008).
8. Holliday, J. A., Ritland, K. & Aitken, S. N. Widespread, ecologically relevant genetic markers developed from association mapping of climate-related traits in Sitka spruce (Picea sitchensis). *New Phytologist* **188,** 501–514; 10.1111/j.1469-8137.2010.03380.x (2010).
9. Chen, J. *et al.* Clinal variation at phenology-related genes in spruce: parallel evolution in FTL2 and Gigantea? *Genetics* **197,** 1025–1038; 10.1534/genetics.114.163063 (2014).
10. González-Martínez, S. C., Ersoz, E., Brown, G. R., Wheeler, N. C. & Neale, D. B. DNA sequence variation and selection of tag single-nucleotide polymorphisms at candidate genes for drought-stress response in Pinus taeda L. *Genetics* **172,** 1915–1926; 10.1534/genetics.105.047126 (2006).
11. González-Martínez, S. C., Krutovsky, K. V. & Neale, D. B. Forest-tree population genomics and adaptive evolution. *New Phytologist* **170,** 227–238; 10.1111/j.1469-8137.2006.01686.x (2006).
12. Yeaman, S. *et al.* Convergent local adaptation to climate in distantly related conifers. *Science (New York, N.Y.)* **353,** 1431–1433; 10.1126/science.aaf7812 (2016).
13. Scotti, I., González-Martínez, S. C., Budde, K. B. & Lalagüe, H. Fifty years of genetic studies: what to make of the large amounts of variation found within populations? *Annals of Forest Science* **73,** 69–75; 10.1007/s13595-015-0471-z (2016).
14. Alexandre, H. *et al. In situ estimation of genetic variation of functional and ecological traits in Quercus petraea and Q.robur* (2018).
15. Dungey, H. S. *et al.* Phenotyping Whole Forests Will Help to Track Genetic Performance. *Trends in Plant Science* **23,** 854–864; 10.1016/j.tplants.2018.08.005 (2018).

16. Martínez-Sancho, E. *et al.* The GenTree Dendroecological Collection, tree-ring and wood density data from seven tree species across Europe. *Scientific Data* **7,** 1; 10.1038/s41597-019-0340-y (2020).

17. Raquel Benavides *et al.* The GenTree Leaf Collection: inter- and intraspecific leaf variation in seven forest tree species in Europe. *Global Ecology and Biogeography*; 10.1111/geb.13239.

18. Steinbach, D. *et al.* GnpIS: an information system to integrate genetic and genomic data from plants and fungi. *Database : the journal of biological databases and curation* **2013,** bat058; 10.1093/database/bat058 (2013).

19. Lotterhos, K. E. & Whitlock, M. C. Evaluation of demographic history and neutral parameterization on the performance of FST outlier tests. *Molecular Ecology* **23,** 2178–2192; 10.1111/mec.12725 (2014).

20. Lotterhos, K. E. & Whitlock, M. C. The relative power of genome scans to detect local adaptation depends on sampling design and statistical method. *Molecular Ecology* **24,** 1031–1046; 10.1111/mec.13100 (2015).

21. Eckert, A. J. *et al.* Back to nature: ecological genomics of loblolly pine (Pinus taeda, Pinaceae). *Molecular Ecology* **19,** 3789–3805; 10.1111/j.1365-294X.2010.04698.x (2010).

22. Lind, B. M. *et al.* Water availability drives signatures of local adaptation in whitebark pine (Pinus albicaulis Engelm.) across fine spatial scales of the Lake Tahoe Basin, USA. *Molecular Ecology* **26,** 3168–3185; 10.1111/mec.14106 (2017).

23. Canham, C. D., LePage, P. T. & Coates, K. D. A neighborhood analysis of canopy tree competition: effects of shading versus crowding. *Can. J. For. Res.* **34,** 778–787; 10.1139/x03-232 (2004).

24. Lorimer, C. G. Tests of age-independent competition indices for individual trees in natural hardwood stands. *Forest Ecology and Management* **6,** 343–360 (1983).

25. Leempoel, K. *et al.* Very high-resolution digital elevation models: are multi-scale derived variables ecologically relevant? *Methods Ecol Evol* **6,** 1373–1383; 10.1111/2041-210X.12427 (2015).

26. Conrad, O. *et al.* System for Automated Geoscientific Analyses (SAGA) v. 2.1.4. *Geosci. Model Dev. Discuss.* **8,** 2271–2312; 10.5194/gmdd-8-2271-2015 (2015).

27. Hengl, T. *et al.* Mapping Soil Properties of Africa at 250 m Resolution: Random Forests Significantly Improve Current Predictions. *PLoS ONE* **10,** e0125814; 10.1371/journal.pone.0125814 (2015).

28. Karger, D. N. *et al.* Climatologies at high resolution for the earth's land surface areas. *Scientific Data* **4,** 170122; 10.1038/sdata.2017.122 (2017).

29. Hijmans, R. & van Etten, J. *raster: Geographic data analysis and modeling. R package* (2016).

30. Mauri, A., Strona, G. & San-Miguel-Ayanz, J. EU-Forest, a high-resolution tree occurrence dataset for Europe. *Scientific Data* **4,** 160123; 10.1038/sdata.2016.123 (2017).

31. Opgenoorth L, Dauphin B, Benavides R, Heer K; Alizoti P; Martínez-Sancho E et al. Supporting data for "The GenTree Platform: growth traits and tree-level environmental data in twelve European forest tree species" GigaScience Database. 2021 http://dx.doi.org/10.5524/100855

32. Opgenoorth L, Dauphin B, Benavides R, Heer K; Alizoti P; Martínez-Sancho E et al. Data for “The GenTree Platform”. Figshare database. https://figshare.com/s/4d57474fd63864a6dfd8
