# Supplementary material for: The GenTree Platform: growth traits and tree-level environmental data in 12 European forest tree species
Source: Gigascience. 2021 Mar 18;10(3):giab010. doi: 10.1093/gigascience/giab010 (PMC7970660; doi:10.1093/gigascience/giab010)
Supplement: giab010_Response_to_Reviewer_Comments_Original_Submission [file giab010_response_to_reviewer_comments_original_submission.pdf]

Author's Response To Reviewer Comments

Close

General comments from the Editor
The reviewers' reports are below. Please pay particular attention to reviewer 1's points regarding sampling methodology and data structure (e.g. will it be possible to include time series / repeated measurements?).
Please include a point-by-point within the 'Response to Reviewers' box in the submission system. Please ensure you describe additional experiments that were carried out and include a detailed rebuttal of any criticisms or requested revisions that you disagreed with. Please also ensure that your revised manuscript conforms to the journal style, which can be found in the Instructions for Authors on the journal homepage. If the data and code has been modified in the revision process please be sure to update the public versions of this too.

Reviewer reports:
Reviewer #1
This Data Note introduces the 'GenTree Platform' - consisting of phenotypic (coarse tree-level traits) and environmental data at individual tree level from ~5000 trees of 12 important European species. At each tree, a set of traits are scored and data taken on soil depth, vegetation and other site data at 194 sites (covering ranges of species and environmental gradients). Modelled climate and soil data (i.e., from layers) have been extracted from external datasets and presented as well. Populations (25 trees) were sampled at up to 20 sites per species.
The dataset forms a new fragment of a larger database 'GenTree' with leaf trait, genomic and dendroecological nodes. It is not clear why each of these modules should be treated individually when they appear to deal with the same trees but perhaps that is for historical and practical reasons.
The dataset appears to be well formulated, significant in size and collection/collaboration effort, and is a useful and novel addition to functional ecology data available, in that it is presented in an easily accessible way and provides data not easily otherwise available. The gentree dataset as a whole appears to be significant in its standardised methods and depth of data at tree level on many facets of genetics, traits and environment.
MAJOR COMMENTS:
1. I don't think there is any reference to repeat measurements other than mention of tagging for 'potential subsequent additional ... sampling'? Does this dataset represent a single measurement - presumably it does? Is it intended that these tagged trees and sites be re-measured? If so, with what protocol and frequency (i.e. what needs to be repeated and what doesn't) and how will that data be integrated or presented with these?
The reviewer is right, that there is no resampling scheme in place. Instead, the permanent marking of trees is meant to offer the chance either to individual studies aimed just at a subset of sites/trees or to a later comprehensive project to resample trees and thus make use of time-series data. For example, there is a new EU project called FORGENIUS (http://www.euforgen.org/about-us/news/news-detail/forgenius-a-new-project-to-revolutionise-our-understanding-of-forest-diversity/), that will start January 1st and will make use of a subset of the sites/trees by revisiting them and obtaining additional phenotypic measurements. That said, we have now stated this point explicitly in the text starting in line 338:
"Every tree was permanently labeled so that future studies can resample subsets or the entire GenTree collection for gaining time-series data of individual traits or to add new phenotypes to the analyses. Be aware, that permission of the respective landowners must be obtained prior to sampling."
2. The data are arranged by country/species/population/individual as far as I can tell - how are measurements in a time series (whether it yet exists or not) identified - should there be a visit number, date or ID field as well? I don't get the feeling that future data addition and re-sampling has been incorporated specifically in the protocols or data formatting. This needs to be addressed in the manuscript and the date/year or sampling should appear in the data in any case (a visit or measurement series number field may also be useful as data are added temporally).
We agree with the reviewer and have thus added columns with the respective visiting dates in the data frame.
3. The introductory parts suggest that the dataset addresses limitations in high-resolution environmental

information associated with trees. Certainly, the site-based soil and topography measurements address this, but the additional soil grids and CHELSA climate data do not increase environmental information, only extract and present it alongside the site data in a convenient analysis-ready format. Please revise the text to be clear that this aspect of the dataset is a compilation/merge of data, not the creation of new high-resolution environmental data.
It is correct that only our on-site measurements are really highly resolved environmental data in the given context and that some of the modeled data resolves mainly on site-scale. However, some of the modelled data is downscaled to tree-level which is an added value, a step that does increase the environmental information on top of the noted convenience. However, we agree that we should be more specific here as to not raise wrong expectations in the introduction. The text in line 264 ff now reads:
"However, progress in this field has been hampered by limited genomic resources, the lack of small-scale, individual tree-level-resolution environmental information 13…. In addition to onsite environmental measurements, we provide modelled and downscaled data for our sites and for individual tree level respectively."
4. Line numbers would have been convenient for reference.
We are sorry! We have included them now.
MINOR COMMENTS:
5. Abstract: Please correct inconsistent capitalisation of common names.
I assume you are referring to the spelling of Silver fir – which should be silver fir. Thank you for pointing it out. Just in case you are referring to the fact that some of the names are in capitals and others not – this is intentional – as the correct spelling varies – e.g. when a country name is involved as in Swiss stone pine – then Swiss needs to be capitalized.
6. CONTEXT - I think the context should give brief 'context' of the data type presented in relation to other datasets. There are clear differences compared to species and plot level measurements of tree traits and cover etc. but it could be useful for a potential user to get a feel for this here given there are now a number of databases. What other available datasets include comparable individual tree data? There will be a number for dbh at least but the value of the gentree dataset seems to lie in the depth of other information collected and compiled for each tree, whereas other forest datasets focus more on good distribution/population data for stands in height, dbh, mortality etc. of many trees - so please make this point of difference clear here (individual versus stand level data) if that is the correct focus. What is different about the dataset and how does it fit with others?
We appreciate the need to give some background on other available datasets/databases. We have added this paragraph starting in line 283:
"We investigated the extent to which other datasets comparable to the data presented here exist by screening our twelve species in the TRY Plant Trait Data Base, the ITDRB, and the biomass and allometry data base for woody plants (BAAD). While this is a systematic approach, it leaves out a large number of tree species and therefore we cannot claim to have a comprehensive overview of the existing data. However, all three databases are large collections that include at least some of the tree measurements we present. Even though these are tremendous resources, the major difference is that based on their nature as collecting points of numerous independent datasets, there is no coherent sampling scheme in these collections as such, meaning that the number of trees per site, the way of tree selection, measured phenotypes, and provided environmental information vary greatly and therefore do not allow for coherent comparative analyses such as those of the GenTree Platform. For example, BAAD reports DBH data for only four of the species presented here, namely Betula pendula with three populations, Fagus sylvatica with two populations, Picea abies with four populations, and Pinus sylvestris with ten populations. In the larger TRY database, all of our species are represented, but the variability of sampling schemes is much more heterogenous in relation to traits, number of populations per species, and metadata. For example, DBH measurements are being reported 232 times from a total of 12 Betula pendula populations. Of these the vast majority of the 170 measurements are from one population while from many other populations only one or up to five measurements are reported. Also, the measurements stem from five different original studies and thus having very different levels of additional information. We conclude that the core value of our reported data lies in the coherent sampling design and yet large number of sampled populations and individuals per species."
7. First sentence reads as though climate and land use changes will only have a future and not a present-day impact.
Changed. It now states starting in line 235:
"The impacts of climate change and land-use change on forests are already severe, as observed, for example, following the extreme summer drought of 2018 that triggered a massive increase in mortality in Central European forests1. Furthermore, changes are expected to be acute in the future,"
8. re:'In the light of these changes, species and forest ecosystem resilience will depend on the extent and structure of genetic variation and adaptive potential.' What about physiological resilience/acclimation at individual level as well as dispersal?

Changed. The sentence now states starting in line 244:
“In the light of these changes, species and forest ecosystem resilience will depend on the extent and structure of phenotypic plasticity, genetic variation and adaptive potential, as well as dispersal ability.”
9. re:'extensive gene flow' Could extensive gene flow not work against local adaptation?
It does for sure. The term “extensive gene flow” was misplaced in the flow of the sentence. It now reads starting in line 248:
“In Europe, where most tree populations have established following post-glacial recolonization, such patterns of local adaptation must have developed rapidly and despite long generation time and extensive gene flow , a process enabled by high levels of within-population plasticity, genetic and epigenetic variation, and large population sizes .”
Thank you for pointing it out!
10. METHODS - 'Growth' traits are only mentioned in the title - can you define or refer to this phrase here? It is only called 'tree phenotypes' here. Growth could equally be structural or some other word - from the title I was expecting traits relating to growth rate or mode.
We exchanged the term tree phenotype with growth traits in line 389, as essentially all our tree level phenotypes can be regarded as growth traits.
11. Vegetation cover is estimated visually in coarse % categories. Is a proportion of that estimate is made up by the subject tree, or does that cover estimate exclude the tree itself? Does it include canopy/shrub/ground layers?
It only included ground vegetation – not the tree cover itself. We have now made this clear by adding “without tree cover” in the following sentence in line 377:
“Surrounding each target tree, slope, vegetation cover (without tree cover), and stone content were assessed in a 10 m x 10 m plot.”
12. re:'show no signs of significant damage due to pests and diseases or generally low vigor' what is the justification for this? Can't stress/pest damage be a part of the trees' natural state in a given environment? e.g., how would this affect future repeat sampling of these permanently marked trees if, as you say, climate and land-use changes are expected to significantly impact tree regeneration, growth and health? Or how would one go about sampling additional sites with your method if there were no healthy trees remaining?
You are of course right, that stress/pest damage are part of the trees natural states and could even be extremely interesting in association studies. However, given that each of such states would be additional “treatments” – our number of repeats on the single tree level would just not be sufficient to adequately analyze this. For this reason, we decided to choose only “normal” “healthy” trees.
13. Regeneration: why are seedlings not counted in the plot but scored categorically? Is it to save time?
Yes. Unfortunately – given that the field campaigns were very time and resource consuming we had to make some tough choices. This was one of them.
14. Climate: while CHELSA is a suitable dataset it isn't especially high resolution by today's standards - it is highly likely, for example, that gridded values extracted at the location of individual trees within a population/site will come from the same grid cell on a climate layer. Obviously, truly individual data would be in the realm of microclimatic measurement, which is not in the scope of this dataset (perhaps a consideration for future repeat sampling?) but is expected based on the abstract and introduction. Needs some re-wording to be clear that this is an extraction/compilation exercise rather than data generation per se, i.e., you are making use of high-resolution products that are now available, and packing that data with your own.
We agree with the comments by the reviewer that CHELSA by itself is not capturing microclimatic conditions and indeed, as indicated in another reply, there is already at least one follow-up project that will utilize some of the sites and will install beside other climate sensors. That said, our extraction method using bilinear interpolation accounts for the surrounding conditions of each tree. We have complemented the original text in the abstract in line 216:
“…and environmental modeling data extracted by using bilinear interpolation accounting for surrounding conditions of each tree (precipitation, temperature, insolation, drought indices) were obtained from trees in 194 sites covering the species’ geographic ranges and reflecting local environmental gradients.”
Also we included this information in the Material and Methods section starting in line 470:
“We extracted all modeled environmental values for each individually geo-referenced tree using the extract function of the R package RASTER (Hijmans and van Etten 2016). The surrounding conditions (i.e. adjacent pixels) of each individual tree were incorporated by the bilinear interpolation method when extracting the data.”

15. Figure 1. Perhaps self-evident, but there is no key - could you at least refer to sites (points) and distributions (dark green) or something in the caption? A useful and clear figure.
Thank you for pointing this out. We have rephrased the figure capture starting in line 501:
“Sampling sites (black dots) and distributions of the twelve selected tree species (dark green shading)

for in-situ phenotype measurements. Distribution maps are based on a comprehensive high-resolution tree occurrence dataset from the European Union."
16. Figures 2&3 are very useful information for re-use, thank you.
Thank you!
17. Data: There isn't an obvious reason why these data can't be presented in a single spreadsheet/file instead of three (metadata, site data and extracted environmental data) given they are in xls format (i.e., tabs for metadata) and the data tables both have fields per individual tree. Wouldn't that make it easier to access/store and use? But perhaps data modules are treated separately because there are many more available from other gentree nodes?
Indeed, our rationale is the inter-usability with other GenTree data and products, which is why we would like to retain the structure as is.
18. Unless I have missed it, appears not to have a field referring to survey number/ID/date etc, which means the structure will have to be changed as soon as repeat measures are included. Date/year should be a basic field in any case so that data can be matched to climate seasons/trends over time. The time of sampling appears to be excluded.
See comment above, we have now included this information.
19. Do you mention the open access CC0 licence anywhere in the paper (it appears in the linked figshare dataset)? Users will want to know if it is freely available as some such datasets require permission from the data owners.
Thank you for this note. The sentence now reads in line 482:
"These data files are available at figshare data repository open access under a CC0 license. (https://figshare.com/s/4d57474fd63864a6dfd8)."
Reviewer #2:
1) The manuscript present a relevant dataset for forest scientists. The dataset is broad and valuable. The manuscript is clear and it is well structured and written. Data are stored in figshare where the authors stated that 4 different files are included. However only three files are in the figshare site (https://figshare.com/s/4d57474fd63864a6dfd8) this point should clarify in the manuscript. Additionally , although it is indicated that 4 csv files are available in the repository (https://figshare.com/s/4d57474fd63864a6dfd8) only 3 files are availables and only one it is in csv (the otaher two are xlsx files) I suggest to complete the 4 files and use csv format for all of them.
The reviewer is right in relation to figshare. This was a confusion on our side, since for GigaScience we had to provide all data in csv files to their ftp server, we switched numbers. On the ftp server the env data is in 4 csv files. The way we present the data on figshare is only three files and it is the correct number of files in fact. We thus would like to keep them as they are there with the inclusion of sampling date though.

MINOR COMMENTS
In Methods (competition index at tree level)
1) I would suggest to reorder the author recognition by writing Lorimet et al and Canham et al (and not the reverse as is in the manuscript) because Lorimer et al paper is previous to the Lorimer et al one.
We agree and have changed the order in lines 360 following.
2) when authors note that 'each stem larger than 15 cm..' must be noted that this value is for dbh
Done. We have included the term DBH in line 375.
In Methods (regeneration
1) When talking about 'paternity', should maternity be written instead?
We agree and have changed it accordingly in line 387.
In Methods (tree phenotypes)
1) at the end of DBH (cm) indicate that average is used only when authors dealed with multistem trees.
There are two mentions of average in the DBH section. The first refers to the method when using a caliper. There two perpendicular measurements were made and then the average of the two was taken. We have now included this specification there in line 392:
"DBH was measured at a stem height of 1.3 m using either a caliper by measuring two perpendicular diameters and subsequently taking the average of these two measurements or by measuring the circumference of the tree using a tape and computing the diameter from that value."
In the second sentence, the average was recorded "if a tree had more than one trunk". We think that this usage of average should be clear.
2) In the Heigh section, a clarification about slope correction method used when heigh measurements is needed
We have added the following text in line 400:
"To forego errors introduced by measuring height on sloping ground, height measurements on slopes were conducted from the same elevation as the tree's base by approaching the tree sideways. Where this was not possible, a slope correction factor was used."

3) References - Please check this section some of the referencies are not complete (ie, # 4, 16, 29,...)
We have checked the references and corrected them where necessary. That said, we believe that the specifically mentioned reference #4 was cited correctly according the journals requirements.

Close
