# Supplementary material for: The GenTree Platform: growth traits and tree-level environmental data in 12 European forest tree species
Source: Gigascience. 2021 Mar 18;10(3):giab010. doi: 10.1093/gigascience/giab010 (PMC7970660; doi:10.1093/gigascience/giab010)
Supplement: giab010_Response_to_Reviewer_Comments_Revision_1 [file giab010_response_to_reviewer_comments_revision_1.pdf]

Author's Response To Reviewer Comments

Close

1. I have included the corrected author list
2. I citing the GigaDB repository. However, since the doi is not yet availabl, I followed this advise by Chris Hunter: "Thank you, I will process the dataset as soon as possible. If that is the only bit of information you require to complete the manuscript submission then there is no need for you to wait, the handling editor (CC'd) can insert the dataset citation on your behalf during the copy editing stage."
3. I send a brief explanation via email regarding the additional author.
4. The additional author fits under the contribution listed as was in place.

Close
