# Supplementary material for: The GenTree Platform: growth traits and tree-level environmental data in 12 European forest tree species
Source: Gigascience. 2021 Mar 18;10(3):giab010. doi: 10.1093/gigascience/giab010 (PMC7970660; doi:10.1093/gigascience/giab010)
Supplement: giab010_Reviewer_1_Report_Original_Submission — Greg Guerin -- 8/16/2020 Reviewed [file giab010_reviewer_1_report_original_submission.pdf]

Reviewer Report

Title: The GenTree Platform: growth traits and tree-level environmental data in twelve European forest tree species

Version: Original Submission **Date:** 8/16/2020

Reviewer name: Greg Guerin

Reviewer Comments to Author:

The GenTree Platform: growth traits and tree-level environmental data in twelve European forest tree species
Opgenoorth et al.
This Data Note introduces the 'GenTree Platform' - consisting of phenotypic (coarse tree-level traits) and environmental data at individual tree level from ~5000 trees of 12 important European species. At each tree, a set of traits are scored and data taken on soil depth, vegetation and other site data at 194 sites (covering ranges of species and environmental gradients). Modelled climate and soil data (i.e., from layers) have been extracted from external datasets and presented as well. Populations (25 trees) were sampled at up to 20 sites per species.
The dataset forms a new fragment of a larger database 'GenTree' with leaf trait, genomic and dendroecological nodes. It is not clear why each of these modules should be treated individually when they appear to deal with the same trees but perhaps that is for historical and practical reasons.
The dataset appears to be well formulated, significant in size and collection/collaboration effort, and is a useful and novel addition to functional ecology data available, in that it is presented in an easily accessible way and provides data not easily otherwise available. The gentree dataset as a whole appears to be significant in its standardised methods and depth of data at tree level on many facets of genetics, traits and environment.
Major Comments:
I don't think there is any reference to repeat measurements other than mention of tagging for 'potential subsequent additional ... sampling'? Does this dataset represent a single measurement - presumably it does? Is it intended that these tagged trees and sites be re-measured? If so, with what protocol and frequency (i.e. what needs to be repeated and what doesn't) and how will that data be integrated or presented with these? The data are arranged by country/species/population/individual as far as I can tell - how are measurements in a time series (whether it yet exists or not) identified - should there be a visit number, date or ID field as well? I don't get the feeling that future data addition and re-sampling has been incorporated specifically in the protocols or data formatting. This needs to be addressed in the manuscript and the date/year or sampling should appear in the data in any case (a visit or measurement series number field may also be useful as data are added temporally).
The introductory parts suggest that the dataset addresses limitations in high-resolution environmental information associated with trees. Certainly, the site-based soil and topography measurements address this, but the additional soil grids and CHELSA climate data do not increase environmental information, only extract and present it alongside the site data in a convenient analysis-ready format. Please revise

the text to be clear that this aspect of the dataset is a compilation/merge of data, not the creation of new high-resolution environmental data.

Line numbers would have been convenient for reference.

Minor comments:

Abstract:

Please correct inconsistent capitalisation of common names.

CONTEXT:

I think the context should give brief 'context' of the data type presented in relation to other datasets. There are clear differences compared to species and plot level measurements of tree traits and cover etc but it could be useful for a potential user to get a feel for this here given there are now a number of databases. What other available datasets include comparable individual tree data? There will be a number for dbh at least but the value of the gentree dataset seems to lie in the depth of other information collected and compiled for each tree, whereas other forest datasets focus more on good distribution/population data for stands in height, dbh, mortality etc of many trees - so please make this point of difference clear here (individual versus stand level data) if that is the correct focus. What is different about the dataset and how does it fit with others?

First sentence reads as though climate and land use changes will only have a future and not a present-day impact.

re:'In the light of these changes, species and forest ecosystem resilience will depend on the extent and structure of genetic variation and adaptive potential.' What about physiological resilience/acclimation at individual level as well as dispersal?

re:'extensive gene flow' Could extensive gene flow not work against local adaptation?

METHODS

'Growth' traits are only mentioned in the title - can you define or refer to this phrase here? It is only called 'tree phenotypes' here. Growth could equally be structural or some other word - from the title I was expecting traits relating to growth rate or mode.

Vegetation cover is estimated visually in coarse % categories. Is a proportion of that estimate is made up by the subject tree, or does that cover estimate exclude the tree itself? Does it include canopy/shrub/ground layers?

re:'show no signs of significant damage due to pests and diseases or generally low vigor' what is the justification for this? Can't stress/pest damage be a part of the trees' natural state in a given environment? e.g., how would this affect future repeat sampling of these permanently marked trees if, as you say, climate and land-use changes are expected to significantly impact tree regeneration, growth and health? Or how would one go about sampling additional sites with your method if there were no healthy trees remaining?

Regeneration: why are seedlings not counted in the plot but scored categorically? Is it to save time?

Climate: while CHELSA is a suitable dataset it isn't especially high resolution by today's standards - it is highly likely, for example, that gridded values extracted at the location of individual trees within a population/site will come from the same grid cell on a climate layer. Obviously, truly individual data would be in the realm of microclimatic measurement, which is not in the scope of this dataset (perhaps a consideration for future repeat sampling?) but is expected based on the abstract and introduction. Needs some re-wording to be clear that this is an extraction/compilation exercise rather than data

generation per se, i.e., you are making use of high resolution products that are now available, and packing that data with your own.
Figure 1.
Perhaps self-evident, but there is no key - could you at least refer to sites (points) and distributions (dark green) or something in the caption? A useful and clear figure.
Figures 2&3 are very useful information for re-use, thank you.
Data:
There isn't an obvious reason why these data can't be presented in a single spreadsheet/file instead of three (metadata, site data and extracted environmental data) given they are in xls format (i.e., tabs for metadata) and the data tables both have fields per individual tree. Wouldn't that make it easier to access/store and use? But perhaps data modules are treated separately because there are many more available from other gentree nodes?
Unless I have missed it, appears not to have a field referring to survey number/ID/date etc, which means the structure will have to be changed as soon as repeat measures are included. Date/year should be a basic field in any case so that data can be matched to climate seasons/trends over time. The time of sampling appears to be excluded.
Do you mention the open access CC0 licence anywhere in the paper (it appears in the linked figshare dataset)? Users will want to know if it is freely available as some such datasets require permission from the data owners.

Level of Interest

Please indicate how interesting you found the manuscript: Choose an item.

Quality of Written English

Please indicate the quality of language in the manuscript: Choose an item.

Declaration of Competing Interests

Please complete a declaration of competing interests, considering the following questions:

- Have you in the past five years received reimbursements, fees, funding, or salary from an organisation that may in any way gain or lose financially from the publication of this manuscript, either now or in the future?
- Do you hold any stocks or shares in an organisation that may in any way gain or lose financially from the publication of this manuscript, either now or in the future?
- Do you hold or are you currently applying for any patents relating to the content of the manuscript?
- Have you received reimbursements, fees, funding, or salary from an organization that holds or has applied for patents relating to the content of the manuscript?

- Do you have any other financial competing interests?
- Do you have any non-financial competing interests in relation to this paper?

If you can answer no to all of the above, write 'I declare that I have no competing interests' below. If your reply is yes to any, please give details below.

I declare that I have no competing interests.

I agree to the open peer review policy of the journal. I understand that my name will be included on my report to the authors and, if the manuscript is accepted for publication, my named report including any attachments I upload will be posted on the website along with the authors' responses. I agree for my report to be made available under an Open Access Creative Commons CC-BY license (http://creativecommons.org/licenses/by/4.0/). I understand that any comments which I do not wish to be included in my named report can be included as confidential comments to the editors, which will not be published.

Choose an item.

To further support our reviewers, we have joined with Publons, where you can gain additional credit to further highlight your hard work (see: https://publons.com/journal/530/gigascience). On publication of this paper, your review will be automatically added to Publons, you can then choose whether or not to claim your Publons credit. I understand this statement.

Yes Choose an item.
