# Supplementary material for: The GenTree Platform: growth traits and tree-level environmental data in 12 European forest tree species
Source: Gigascience. 2021 Mar 18;10(3):giab010. doi: 10.1093/gigascience/giab010 (PMC7970660; doi:10.1093/gigascience/giab010)
Supplement: giab010_Reviewer_2_Report_Original_Submission — Felipe Bravo -- 9/6/2020 Reviewed [file giab010_reviewer_2_report_original_submission.pdf]

Reviewer Report

Title: The GenTree Platform: growth traits and tree-level environmental data in twelve European forest tree species

Version: Original Submission **Date:** 9/6/2020

Reviewer name: Felipe Bravo

Reviewer Comments to Author:

The manuscript present a relevant dataset for forest scientists. The dataset is broad and valuable. The manuscript is clear and it is well structured and written. Data are stored in figshare where the authors stated that 4 different files are included. However only three files are in the figshare site (https://figshare.com/s/4d57474fd63864a6dfd8) this point should clarify in the manuscript.
Additionally , although it is indicated that 4 csv files are available in the repository (https://figshare.com/s/4d57474fd63864a6dfd8) only 3 files are availables and only one it is in csv (the otaher two are xlsx files) I suggest to complete the 4 files and use csv format for all of them.
Minor comments:
In Methods (competition index at tree level)
1) I would suggest to reorder the author recognition by writing Lorimet et al and Canham et al (and not the reverse as is in the manuscript) because Lorimer et al paper is previous to the Lorimer et al one.
2) when authors note that 'each stem larger than 15 cm..' must be noted that this value is for dbh
In Methods (regeneration
1) When talking about 'paternity', should maternity be written instead?
In Methods (tree phenotypes)
1) at the end of DBH (cm) indicate that average is used only when authors dealed with multistem trees.
2) In the Heigh section, a clarification about slope correction method used when heigh measurements is needed
References
Please check this section some of the referencies are not complete (ie, # 4, 16, 29,...)

Level of Interest

Please indicate how interesting you found the manuscript: Choose an item.

Quality of Written English

Please indicate the quality of language in the manuscript: Choose an item.

Declaration of Competing Interests

Please complete a declaration of competing interests, considering the following questions:

- Have you in the past five years received reimbursements, fees, funding, or salary from an organisation that may in any way gain or lose financially from the publication of this manuscript, either now or in the future?
- Do you hold any stocks or shares in an organisation that may in any way gain or lose financially from the publication of this manuscript, either now or in the future?
- Do you hold or are you currently applying for any patents relating to the content of the manuscript?
- Have you received reimbursements, fees, funding, or salary from an organization that holds or has applied for patents relating to the content of the manuscript?
- Do you have any other financial competing interests?
- Do you have any non-financial competing interests in relation to this paper?

If you can answer no to all of the above, write 'I declare that I have no competing interests' below. If your reply is yes to any, please give details below.

I declare that I have no competing interests.

I agree to the open peer review policy of the journal. I understand that my name will be included on my report to the authors and, if the manuscript is accepted for publication, my named report including any attachments I upload will be posted on the website along with the authors' responses. I agree for my report to be made available under an Open Access Creative Commons CC-BY license (http://creativecommons.org/licenses/by/4.0/). I understand that any comments which I do not wish to be included in my named report can be included as confidential comments to the editors, which will not be published.

Choose an item.

To further support our reviewers, we have joined with Publons, where you can gain additional credit to further highlight your hard work (see: https://publons.com/journal/530/gigascience). On publication of this paper, your review will be automatically added to Publons, you can then choose whether or not to claim your Publons credit. I understand this statement.

Yes Choose an item.
